# Associations between perceived environmental pollution and health-related quality of life in a Chinese adult population

**DOI:** 10.1186/s12955-020-01442-9

**Published:** 2020-06-23

**Authors:** Bingxue Han

**Affiliations:** 1 International Issues Center, Xuchang University, Xuchang, Henan China; 2 Family Issues Center, Xuchang University, Xuchang, Henan China; 3 Xuchang Urban Water Pollution Control and Ecological Restoration Engineering Technology Research Center, Xuchang University, Xuchang, China; 4 College of Urban and Environmental Sciences, Xuchang University, Xuchang, China

**Keywords:** Perceived environmental pollution, Health-related quality of life, PCS scores, MCS scores, Chinese adult population

## Abstract

**Background:**

Health-related quality of life (HRQoL) measures are being used in increasingly diverse populations. However, there have no known studies to date to examine the associations between perceived environmental pollution and HRQoL in a Chinese population. This study aimed to report the associations between air, water, noise pollution and HRQoL among Chinese adult population.

**Methods:**

A cross-sectional survey data was used from East Asian Social Survey 2010 with a sample of 3866 Chinese populations regarding environmental pollution. HRQoL was computed by SF-12 and reflected by physical and mental component summary score (PCS & MCS). Ordinary least regression analyses were used to examine associations between perceived environmental pollution and PCS and MCS scores. Models in SPSS PROCESS were selected to demonstrate the moderating and mediating effects.

**Results:**

Only considering one pollutant, perceived air pollution and perceived water pollution had significant associations with PCS and MCS scores. Perceived noise pollution had significant associations with PCS scores. Perceived air×noise, air×water, noise×water, and air×noise×water pollution had significant associations with PCS and MCS scores. Conditional (moderated) mediation showed that there were no moderating effects and mediating effects of perceived one pollutant on another pollutant.

**Conclusions:**

Co-occurring perceived environmental pollution were mainly associated with progressive increase in PCS and MCS scores among the Chinese adult population. These results suggested that some effective policies should be carried out to improve environmental quality in Chinese adult population.

## Introduction

A number of studies indicated that air pollution possibly worsened the health status of patients. For example, a study conducted in peri-urban Peru concluded higher traffic air pollution exposure was associated with worse rhinitis QoL among asthmatic children [1]. A small cohort study of patients with fibrotic sarcoidosis indicated that PM2.5 exposure was associated with increased severity of respiratory and quality of life symptoms [2]. A field study suggest that PM, NO_2_, and O_3_ cause respiratory symptoms leading to HRQoL deterioration [3]. Regarding asthma, a study concluded that minimizing exposure to air pollution might improve asthmatic patients’ disease management and HRQoL [4]. The molecular mechanisms of susceptibility were also reported [5]. Clinically, air pollution produced detrimental effects on mental health by various components of air pollution translocated central nervous system [6]. Mentally, PM2.5 was associated with depressive and anxiety symptoms, with associations the strongest among individuals with lower socioeconomic status [7]. A cross-sectional study in two slums of Nairobi indicated that perceived air pollution level was positively associated with perceived health risks [8]. The deleterious effects of air pollution on human health had been consistently documented by many epidemiologic studies worldwide among the general public [9–13]. Exposure to air pollution had been confirmed to reduce enthusiasm of participation in outdoor physical activity [14, 15]. But, the limited information was investigated in China.

A substantial literature showed water pollution impaired the health status substantially. Previous research has established that polluted water caused by economic growth is one the main risks of public health in mainland China [16–18]. There is a growing body of literature that recognizes the role of water pollution in morbidity and mortality in international community [19, 20]. Also, the generalisability of much published research that disease prevalence and mortality were remarkably associated with water pollution is confirmed in China’s context [21]. A number of studies indirectly reflect the association between water pollution and health outcomes, but there was little literature reported the relationship between water pollution and HRQoL. Water pollution was confirmed to be a major risk factor for the past and potential health deterioration. A study in Saudi Arabia highlighted the urgent need for monitoring and controlling wastewater discharge in Wadi Hanifa to ensure public safety [22]. A systematic review concluded consumption of toxicant tainted fishes in deteriorating Ganga water might cause serious illness including cancer [23]. In Bangladesh, water pollution was crucial to evaluate public health risk, particularly among children [24]. A study in The Huai River basin located in eastern China indicated that the current esophageal cancer mortality rate was mainly caused by water pollution from the previous 8 years [25]. But, the truth on the relationship between water pollution and quality of life has not been discovered in Chinese settings.

There was full of literature that addressed the intersection of quality of life and noise pollution worldwide. For instance, a cross-sectional questionnaire-based study shows that individuals with high noise sensitivity (NS) tend to have degraded HRQOL compared to individuals with low NS [26]. A cross-sectional study indicated that noise sensitivity appear to degrade the quality of life of those with a mild traumatic brain injury [27]. A systematic review found there was lack of evidence for noise effects across studies for many of the quality of life [28]. Noise pollution was pervasive even in U.S. protected areas [29]. Workplace health hazard of noise pollution were documented in factories [30], metalwork and woodwork industries [31], and traffic [32]. Methodologically, literature review and empirical studies were adopted to discuss the relationship between noise pollution and quality of life in the international academic circle [33, 34]. In western countries, environmental noise produced by airport caused psychological and physiological disorders [35, 36]. Association between noise pollution and some specific diseases were reported [37, 38]. Strategically, various countries and international organizations were making efforts to eliminate adverse public health due to environmental noise pollution [39]. But to date, there is little knowledge of the relationship revealed in mainland China.

Several prior studies used SF-12 to measure HRQoL due to its scientific and comprehensive nature [40, 41]. In this study, SF-12 was used to evaluate the associations between three pollutions and HRQoL in a Chinese adult population. Regarding the psychometric properties, several studies have reported the SF-12 was translated and confirmed to be a reliable and valid measure of HRQoL in a range of medical conditions [42], as well as in the general population [43]. Also, the standard SF-12 was valid and equivalent to measure HRQoL for the Chinese [44].

Since the first year in the new century, especially from 2008 to 2012, environmental pollution has become the primary problem restricting economic development and impacting health status among the public in China. Thus, it could be speculated that air, water, and noise pollution had become substantial environmental issues affecting health status and HRQoL. Here, this study adopted SF-12 to explore the associations between perceived environmental pollution and HRQoL in a Chinese adult population. Here, HRQoL was expressed by physical and mental component summary score (PCS & MCS) after the calculation of SF-12. The co-occurring perceived environmental pollution was constructed in the main multiple research scenarios. Interactions between perceived environmental pollution would be analyzed in the scenarios.

## Methods

### Data source

This study used cross-sectional survey data (East Asian Social Survey, http://www.eassda.org/) from a three stage PPS of all Chinese aged 18 and above. This cross-sectional survey data were derived from the China Comprehensive Social Survey (CGSS 2010), which was jointly conducted by Renmin University of China and relevant academic institutions. Fieldwork Dates were from July to December, 2010. Its initial sample size was 5370. But, a valid response rate was 71.99%. After the respondents in Japan, South Korea, and Taiwan were deleted, the China sample size was 3866.

The sampling design made in CGSS 2010 was multi-stage stratified design (http://cgss.ruc.edu.cn/index.php?r=index/sample). There were 3 sampling stages: primary sampling units (PSUs) were county-level units, there were 2762 PSUs in the sampling frame; secondary sampling units (SSUs) were community-level units; 25 households in third-level sampling units were sampled with PPS method in selected SSUs; 18 and above adult were sampled with Kish grid in each selected household. The total sample size of 2010 design was 12,000 households and 2000 were in self-representative stratum.

The questionnaire was mainly composed of health status, health behavior, medical care, medical insurance/social security insurance, alternative medicine, social support/ social trust, environment, epidemiology, family care need and care management, worries about ageing, addiction, and body shape. The data were collected by face-to-face interviewing.

### Dependent variable

The Physical Functioning (PF), Role Physical (RP), Bodily Pain (BP), General Health (GH), Vitality (VT), Social Functioning (SF), Role Emotional (RE), Mental Health (MH), Physical and Mental Composite Scores (PCS & MCS) were calculated with the sf12 program (Bruun, 2015; https://ideas.repec.org/c/boc/bocode/s458125.html) [45]. A higher score in the respective summary scales represent a higher level of functioning. Their mean score (SD), Floor (%), and Ceiling (%) could be seen in Table 1. Thus, mean MCS-12 score was lower than the mean PCS-12 score of the sample.

**Table 1:** Mean score (SD), Floor (%), and Ceiling (%) for 8 domains of SF-12 (total = 3866)

	Observations	Mean score	SD	Floor (%)	Ceiling (%)	Completion rates
PF	3847	50.540	9.724	4.86	66.36	99.51
RP	3842	47.770	9.832	2.06	33.13	99.38
BP	3848	47.382	12.471	4.76	49.32	99.53
GH	3861	49.377	12.276	4.14	24.71	99.87
VT	3859	54.673	10.732	2.54	24.64	99.82
SF	3849	46.530	10.663	3.04	40.11	99.56
RE	3859	45.164	9.978	0.73	30.97	99.82
MH	3847	50.877	10.058	0.10	15.34	99.51
PCS	3781	49.415	10.939	0.03	0.03	97.80
MCS	3781	48.914	9.357	0.03	0.03	97.80

### Main variables

The sociodemographic factors that were of interest to this study included age (years), sex (0 = female,1 = male), marital status, religious status, community type (1 = a big city, 2 = the suburbs or outskirts of a big city, 3 = a town or a small city, and 4 = a country village), years of schooling, body mass index, employment relationship, number of household members, total household income (Chinese Yuan), and chronic conditions (1 = with,0 = without). The marital status included married (=1) and unmarried (=0, widowed, divorced, separated (have intention to divorce), never married, and cohabiting). The religious status included “no religion” (=1) and “religion” (=0, Roman Catholic, Protestant, Islam, Buddhism, and other eastern religions). The variable, employment relationship, was categorized into employment (employee, self-employed without employees, self-employed with employees, and working for own family’s business) coded 0 and unemployment (having no current work income) coded 1. Frequencies of alcoholic drinks was divided by yes (1 = daily, several times a week, several times a month, and several times a year or less often) and no (0 = never). Frequencies of smoking was divided by yes (1 = daily, several times a week, several times a month, and several times a year or less often) and no (0 = never).

Perceived air/noise/water pollution was assessed by the questions: “How severe is air pollution in the area of respondents’ local residence”, “How severe is noise pollution in the area of respondents’ local residence”, and “How severe is water pollution in the area of respondents’ local residence”. Their response options were very severe (=1), somewhat severe (=2), not so severe (=3), and not severe at all (=4) and recoded as yes (1 = very severe and somewhat severe) and no (0 = somewhat severe and not so severe).

### Statistical methods

In the dataset, the response options with “tw: not asked”, “dk, refused”, and “undisclosed” were cleaned as missing values. Descriptive statistics for the sample were calculated on the basis of gender difference. Similarly, air, water, and noise pollution were dichotomized into binary values (1 = very severe and somewhat severe, 0 = not so severe and not severe at all). Thus, four binary variables, including air×water, noise×water, air×noise, and air×noise×water were produced in order to reflect co-existing pollution. These binary variables were valued as 1 if co-occurring pollution existed and as 0 if not. For convenience, the main variables, the abbreviated in the parentheses of marital status (*maritaln*), religious status (*religion*), community type (*urbrural*), years of schooling (*educyrs*), body mass index (*bmi*), number of household members (*hhdnum*), total household income (*cn_hinc*), and chronic conditions (*chronic*), frequencies of alcoholic drinks (*alcoholicg*),and frequencies of smoking (*smokingg*) were adopted in the regression analyses.

Considering confounding factors in a stepwise fashion, the associations between potential covariates and PCS and MCS scores were assessed using linear regression models. The chest command in Stata was used to examine confounding effects for perceived air pollution, water pollution, noise pollution, air×water pollution, air×noise pollution, noise×water pollution, and air×noise×water pollution separately. The socioeconomic factors, lifestyle, and health status were considered as potential confounding variables. When screening removed variables, change-in-estimate criterion with a 0.09% cutoff was adopted [46, 47].

Controlling for the covariates in the linear regression models, moderation and mediation analysis would be performed to reflect the association between perceived environmental pollution and HRQoL. The effect of one pollutant on PCS and MCS scores could be moderated another two pollutants. There was no current literature to support one pollutant did a harm through another pollutant. Thus, when air pollutant was conceived as independent variables, noise and water pollutants were considered as hypothesized moderators rather than mediators. Thus, the moderation could be performed by moderation models one by one in AF Hayes Process procedure for SPSS Release 2.13 (www.afhayes.com; www.guilford.com/p/hayes3) and tested in the simple moderation models 1, 2, and 3. If the statistical outcomes could not reach satisfied significance, some moderated mediation models would be explored in the other complex models. One of PCS and MCS scores could be mediator, the other could be outcome variable. If one pollutant could be independent variable, the other two pollutants could be moderators.

The dependent variables were constructed with PCS and MCS scores. All data were analyzed using the weighted survey procedure in Stata 14.0.

## Results

### Descriptive analysis

Table 2 summarized the demographic characteristics of respondents. Respondents were predominantly female (51.58%), aged 36–58 years (51.54%), married (79.57%), religionless (87.18%), urban (62.47%), employed (64.25%), without chronic conditions (66.01%), without alcoholic drinks (69.20%), without smoking (62.20%), without perceived air pollution (70.96%), without perceived noise pollution (70.10%), without perceived water pollution (73.57%), without perceived air×noise pollution (81.40%), without perceived air×water pollution (82.11%), without perceived noise×water pollution (88.83%), and without perceived air×noise×water pollution (87.44%).

**Table 2:** Sample characteristics by gender

Participant characteristics	Female	Male	Total	Ch2	*P* value
Age (Years), Median (IQR)	46 (36–58)	47 (36–59)	46 (36–58)	86.4658	0.152
Marital Status				0.6203	0.431
Unmarried, N (%)	415 (10.79)	371 (9.64)			
Married, N (%)	1568 (40.76)	1493 (38.81)			
Religion				18.1988	0.000***
Religionless, N (%)	1688 (43.81)	1671 (43.37)			
Religious, N (%)	299 (7.76)	195 (5.06)			
Community Type				3.0775	0.079*
Urban, N (%)	1272 (32.90)	1143 (29.57)			
Rural, N (%)	722 (18.68)	729 (18.86)			
Years of schooling, Median (IQR)	9 (6–11)	9 (6–12)	9 (6–12)	127.5275	0.000***
BMI, Median (IQR)	21.64 (19.56–24.34)	22.49 (20.52–24.80)	22.04 (20.03–24.61)	2.2e+ 03	0.000***
Employment				90.2070	0.000***
Employed, N (%)	1129 (29.57)	1324 (34.68)			
Unemployed, N (%)	847 (22.18)	518 (13.57)			
Number of household members, Median (IQR)	2 (2–3)	2 (2–3)	2 (2–3)	13.7062	0.133
Total household income, Median (IQR)	24,000 (12000–45,000)	24,000 (12000–45,600)	24,000 (12000–45,000)	544.1617	0.609
Chronic Condition				14.6145	0.000***
Without, N (%)	1260 (32.59)	1292 (33.42)			
With, N (%)	734 (18.99)	580 (15.00)			
Alcoholic drinks				1.3e+ 03	0.000***
No, N (%)	1891 (49.23)	767 (19.97)			
Yes, N (%)	84 (2.19)	1099 (28.61)			
Smoked				1.0e+ 03	0.000***
No, N (%)	1706 (44.49)	679 (17.71)			
Yes, N (%)	267 (6.96)	1183 (30.85)			
Air pollution				1.0092	0.315
No, N (%)	1395 (36.18)	1341 (34.78)			
Yes, N (%)	591 (15.33)	529 (13.72)			
Noise pollution				0.5241	0.469
No, N (%)	1404 (36.40)	1300 (33.70)			
Yes, N (%)	584 (15.14)	569 (14.75)		0.1518	0.697
Water pollution					
No, N (%)	1465 (38.03)	1369 (35.54)			
Yes, N (%)	519 (13.47)	499 (12.95)			
Air×noise pollution				0.3983	0.528
No, N (%)	1609 (41.74)	1529 (39.66)			
Yes, N (%)	377 (9.78)	340 (8.82)			
Air×water pollution				0.1151	0.734
No, N (%)	1625 (42.20)	1537 (39.91)			
Yes, N (%)	359 (9.32)	330 (8.57)			
Noise×water pollution				0.3881	0.533
No, N (%)	1684 (43.73)	1598 (41.50)			
Yes, N (%)	300 (7.79)	269 (6.99)			
Air×noise×water pollution				0.0663	0.797
No, N (%)	1732 (44.98)	1635 (42.46)			
Yes, N (%)	252 (6.54)	232 (6.02)			

Note: ***, ** and * indicates 1, 5 and 10% significance level, respectively

Considering gender difference, there were significant differences in religion group, community type, years of schooling, body mass index, employment relationship, chronic condition, frequencies of alcoholic drinks, and frequencies of smoking.

### Regression analysis

Associations of PCS and MCS scores with sociodemographic factors, lifestyle, and perceived environmental pollution were conducted with ordinary least regressions.

### Single perceived environmental pollution

Supplementary Table 1 showed that the change-in-estimates of *hhdnum* (=0.02%) and *smokingg* (=0.01%) were lower than the 0.09% cutoff criterion, which indicated they were covariates. However, the change-in-estimates of age (=3.30%), sex (=0.83%), *maritaln* (=1.78%), *religion* (=0.13%), *urbruraln* (=0.70%), *educyrs* (=180.00%), *bmi* (=6.45%), *employment* (=0.80%), *cn_hinc* (=0.31%), *chronic* (=36.13%), and *alcoholicg* (=0.19%) were higher than the 0.13% cutoff criterion, which indicated they were potential confounding variables.

Supplementary Table 2 showed that the change-in-estimates of *smokingg* (=0.05%) was lower than the 0.09% cutoff criterion, which indicated they were covariates. However, the change-in-estimates of age (=0.5098%), sex (=3.2276%), *maritaln* (=1.16%), *religion* (=0.28%), *urbruraln* (=69.33%), *educyrs* (=11.03%), *bmi* (=8.11%), *employment* (=0.4911%), *hhdnum* (=0.68%)*, cn_hinc* (=1.94%), *chronic* (=10.91%), and *alcoholicg* (=0.16%) were higher than the 0.15% cutoff criterion, which indicated they were potential confounding variables.

Supplementary Table 3 showed that the change-in-estimates of *bmi* (=0.00%) and *hhdnum* (=0.02%) were lower than the 0.09% cutoff criterion, which indicated they were covariates. However, the change-in-estimates of age (=1.3050%), sex (=0.6354%), *maritaln* (=0.48%), *religion* (=0.10%), *urbruraln* (=4.3993%), *educyrs* (=362.3407%), *employment* (=4.82%), *cn_hinc* (=0.09%), *chronic* (=47.0650%), *alcoholicg* (=0.4049%), and *smokingg* (=3.6444%) were higher than the 0.09% cutoff criterion, which indicated they were potential confounding variables.

Supplementary Table 4 showed that the change-in-estimates of *bmi* (=0.0692%) and *smokingg* (=0.0508%) was lower than the 0.09% cutoff criterion, which indicated they were covariates. However, the change-in-estimates of age (=1.2981%), sex (=4.3169%), *maritaln* (=0.0974%), *religion* (=0.5219%), *urbruraln* (=25.0116%), *educyrs* (=108.2362%), *employment* (=1.1174%), *hhdnum* (=0.2426%), *cn_hinc* (=0.6419%), *chronic* (=21.1382%), and *alcoholicg* (=0.2316%) were higher than the 0.09% cutoff criterion, which indicated they were potential confounding variables.

Supplementary Table 5 showed that the change-in-estimates of *religion* (=0.05%) was lower than the 0.09% cutoff criterion, which indicated they were covariates. However, the change-in-estimates of age (=2.61%), sex (=1.03%), *maritaln* (=1.99%), *urbruraln* (=32.76%), *educyrs* (=6.03%), *bmi* (=1.99%), *employment* (=6.26%), *hhdnum* (=0.14%), *cn_hinc* (=0.28%), *chronic* (=20.87%), *alcoholicg* (=0.15%), and *smokingg* (=0.99%) were higher than the 0.14% cutoff criterion, which indicated they were potential confounding variables.

Supplementary Table 6 showed that change-in-estimates of *religion* (=0.02%) was lower than the 0.09% cutoff criterion, which indicated they were covariates. However, the change-in-estimates of age (=1.03%), sex (=4.30%), *maritaln* (=2.79%), *urbruraln* (=22.92%), *educyrs* (=1.49%), *bmi* (=1.81%), *employment* (=1.08%), *hhdnum* (=2.8543%), *cn_hinc* (=2.61%), *chronic* (=9.17%), *alcoholicg* (=0.10%), and *smokingg* (=0.24%) were higher than the 0.09% cutoff criterion, which indicated they were potential confounding variables.

Table 3 showed the associations between one perceived environmental pollution and QoL. Only considering air pollution, perceived air pollution had significant associations with PCS scores (β = 11.442, 95%CI: 9.589,13.295) and MCS scores (β = 32.329, 95%CI: 30.924,33.734), perceived noise pollution had significant associations with PCS scores (β = 1.670, 95%CI: 0.649,2.691), perceived water pollution had significant associations with PCS scores (β = 44.099, 95%CI: 42.918,45.281) and MCS scores (β = 42.561, 95%CI: 41.454,43.669).

**Table 3:** Associations between one perceived environmental pollution and PCS and MCS scores, Coefficients (95%CI)

	Air pollution		Noise pollution		Water pollution	
	PCS scores	MCS scores	PCS scores	MCS scores	PCS scores	MCS scores
Air pollution	11.442*** (9.589,13.295)	32.329*** (30.924,33.734)				
Noise pollution			1.670*** (0.649,2.691)	−0.069 (−0.981,0.842)		
Water pollution					44.099*** (42.918,45.281)	42.561*** (41.454,43.669)
Hhdnum	10.738*** (9.951,11.524)		1.719*** (1.234,2.204)			
Smokingg	17.064*** (14.577,19.551)	40.310*** (39.333,41.287)		1.818*** (0.979,2.658)		
BMI			1.945*** (1.882,2.008)	2.115*** (2.088,2.143)		
Religion					35.643*** (33.246,38.041)	35.760*** (33.459,38.061)
R-squared	0.823	0.478	0.942	0.949	0.313	0.317
Number of observation	3750	3751	3771	3748	3763	3763

Note: ***, ** and * indicates 1, 5 and 10% significance level, respectively. Hhdnum = number of household members. Smokingg = frequencies of smoking. Religion = religious status

*Hhdnum* had significant associations with PCS scores (β = 10.738, 95%CI: 9.951, 11.524) in air polluted population and PCS scores (β = 1.719, 95%CI: 1.234, 2.204) in the population with perceived noise pollution. *Smokingg* had significant associations with PCS scores (β = 17.064, 95%CI: 14.577, 19.551) and MCS scores (β = 40.310, 95%CI: 39.333, 41.287) in the population with perceived air pollution, MCS scores (β = 1.818, 95%CI: 0.979, 2.658) in the population with perceived noise pollution. *Bmi* had significant associations with PCS scores (β = 1.945, 95%CI: 1.882, 2.008) and MCS scores (β = 2.115, 95%CI: 2.088, 2.143) in the population with perceived noise pollution. *Religion* had significant associations with PCS scores (β = 35.643, 95%CI: 33.246, 38.041) and MCS scores (β = 35.760, 95%CI: 33.459, 38.061) in the population with perceived water pollution.

### Two co-occurring perceived environmental pollution

Supplementary Table 7 showed that the change-in-estimates of *hhdnum* (=0.04%) were lower than the 0.09% cutoff criterion, which indicated they were covariates. However, the change-in-estimates of age (=0.73%), sex (=0.30%), *maritaln* (=0.47%), *religion* (=0.13%), *urbruraln* (=1.31%), *educyrs* (=286.62%), *bmi* (=7.64%), *employment* (=0.84%), *cn_hinc* (=0.15%), *chronic* (=51.16%), *alcoholicg* (=0.12%), and *smokingg* (=0.93%) were higher than the 0.13% cutoff criterion, which indicated they were potential confounding variables.

Supplementary Table 8 showed that the change-in-estimates of *smokingg* (=0.08%) were lower than the 0.09% cutoff criterion, which indicated they were covariates. However, the change-in-estimates of age (=0.86%), sex (=0.38%), *maritaln* (=0.34%), *religion* (=0.13%), *urbruraln* (=81.15%), *educyrs* (=17.45%), *bmi* (=9.42%), *employment* (=0.29%), *hhdnum* (=1.71%), *cn_hinc* (=0.9726%), *chronic* (=18.24%), and *alcoholicg* (=0.17%) were higher than the 0.12% cutoff criterion, which indicated they were potential confounding variables.

Supplementary Table 9 showed that the change-in-estimates of *religion* (=0.08%), *hhdnum* (=0.08%), and *alcoholicg* (=0.02%) were lower than the 0.09% cutoff criterion, which indicated they were covariates. However, the change-in-estimates of age (=15.87%), sex (=0.65%), *maritaln* (=0.58%), *urbruraln* (=60.37%), *educyrs* (=2.16%), *bmi* (=0.31%), *employment* (=1.96%), *cn_hinc* (=0.38%), *chronic* (=6.87%), and *smokingg* (=2.89%) were higher than the 0.30% cutoff criterion, which indicated they were potential confounding variables.

Supplementary Table 10 showed that the change-in-estimates of *religion* (=0.03%) and *alcoholicg* (=0.01%) were lower than the 0.09% cutoff criterion, which indicated they were covariates. However, the change-in-estimates of age (=2.44%), sex (=1.09%), *maritaln* (=0.61%), *urbruraln* (=43.09%), *educyrs* (=4.14%), *bmi* (=0.26%), *employment* (=0.78%), *hhdnum* (=0.59%), *cn_hinc* (=2.27%), *chronic* (=2.36%), and *smoking* (=0.21%) were higher than the 0.26% cutoff criterion, which indicated they were potential confounding variables.

Supplementary Table 11 showed that the change-in-estimates of *employment* (=0.04%) and *hhdnum* (=0.03%) were lower than the 0.09% cutoff criterion, which indicated they were covariates. However, the change-in-estimates of age (=38.25%), sex (=0.37%), *maritaln* (=2.07%), *religion* (=0.16%), *urbruraln* (=87.79%), *educyrs* (=6.81%), *bmi* (=0.60%), *cn_hinc* (=0.27%), *chronic* (=23.20%), *alcoholicg* (=0.41%), and *smokingg* (=4.23%) were higher than the 0.15% cutoff criterion, which indicated they were potential confounding variables.

Supplementary Table 12 showed that the change-in-estimates of *smokingg* (=0.01%) was lower than the 0.09% cutoff criterion, which indicated they were covariates. However, the change-in-estimates of age (=4.98%), sex (=1.01%), *maritaln* (=1.41%), *religion* (=0.15%), *urbruraln* (=61.63%), *educyrs* (=8.03%)*, bmi* (=0.38%), *employment* (=0.21%), *hhdnum* (=0.30%), *cn_hinc* (=1.8066%), *chronic* (=6.77%), and *alcoholicg* (=0.18%) were higher than the 0.14% cutoff criterion, which indicated they were potential confounding variables.

Table 4 showed the associations between two co-occurring perceived pollution and QoL. The perceived air×noise pollution had significant associations with PCS scores (β = 13.880, 95%CI: 11.037, 16.722) and MCS scores (β = 31.126, 95%CI: 29.218, 33.034). The perceived air×water pollution had significant associations with PCS scores (β = 9.352, 95%CI: 6.529,12.175) and MCS scores (β = 30.913, 95%CI: 28.798,33.028). The perceived noise×water pollution had significant associations with PCS scores (β = 11.051, 95%CI: 7.966,14.135) and MCS scores (β = 30.768, 95%CI: 28.595,32.942).

**Table 4:** Associations between two co-occurring pollution and PCS and MCS scores, Coefficients (95%CI)

	Air×noise		Air×water		Noise×water	
	PCS scores	MCS scores	PCS scores	MCS scores	PCS scores	MCS scores
Air×noise	13.880*** (11.037,16.722)	31.126*** (29.218,33.034)				
Air×water			9.352*** (6.529,12.175)	30.913*** (28.798,33.028)		
Noise×water					11.051*** (7.966,14.135)	30.768*** (28.595,32.942)
Alcoholicg			13.638*** (11.503,15.773)	39.970*** (38.746,41.194)		
Hhdnum	12.566*** (11.821,13.310)		11.505*** (10.692,12.318)		11.951*** (11.116,12.786)	
Religion			3.377* (−0.256,7.009)	32.172*** (29.580,34.765)		
Smokingg		44.011*** (43.210,44.813)				45.298*** (44.551,46.045)
Employment					9.127*** (6.640,11.613)	
R-squared	0.789	0.438	0.801	0.435	0.793	0.426
Number of observation	3774	3751	3745	3746	3726	3747

Note: ***, ** and * indicates 1, 5 and 10% significance level, respectively. Alcoholicg = frequencies of alcoholic drinks. Hhdnum = number of household members. Religion = religious status. Smokingg = frequencies of smoking

Alcoholic had significant associations with PCS scores (β = 13.638, 95%CI: 11.503, 15.773) and MCS scores (β = 39.970, 95%CI: 38.746, 41.194) in the population with perceived air×water pollution. *Hhdnum* had significant associations with PCS scores (β = 12.566, 95%CI: 11.821, 13.310) in the population with perceived air×noise pollution, PCS scores (β = 11.505, 95%CI: 10.692, 12.318) in the population with perceived air×water pollution, and PCS scores (β = 11.951, 95%CI: 11.116, 12.786) in the population with perceived noise×water pollution. *Religion* had significant associations with PCS scores (β = 3.377, 95%CI: − 0.256, 7.009) and MCS scores (β = 32.172, 95%CI: 29.580, 34.765). *Smokingg* had significant associations with MCS scores (β = 44.011, 95%CI: 43.210, 44.813) in the population with perceived air×noise pollution and MCS scores (β = 45.298, 95%CI: 44.551, 46.045) in the population with perceived noise×water pollution. *Employment* had significant associations with PCS scores (β = 9.127, 95%CI: 6.640,11.613) in the population with perceived noise×water pollution.

### Three co-occurring perceived environmental pollution

Supplementary Table 13 showed that the change-in-estimates of *hhdnum* (=0.00%) was lower than the 0.09% cutoff criterion, which indicated they were covariates. However, the change-in-estimates of age (=9.17%), sex (=1.20%), *maritaln* (=2.04%), *religion* (=0.1620%), *urbruraln* (=2.49%), *educyrs* (=196.99%), *bmi* (=0.45%), *employment* (=1.96%), *cn_hinc* (=0.29%), *chronic* (=39.37%), *alcoholicg* (=0.23%), and *smokingg* (=5.99%) were higher than the 0.16% cutoff criterion, which indicated they were potential confounding variables.

Supplementary Table 14 showed that the change-in-estimates of *hhdnum* (=0.03%) was lower than the 0.09% cutoff criterion, which indicated they were covariates. However, the change-in-estimates of age (=3.72%), sex (=1.14%), *maritaln* (=1.50%), *religion* (=0.27%), *urbruraln* (=62.15%), *educyrs* (=12.33%), *bmi* (=0.41%), *employment* (=0.56%), *cn_hinc* (=1.90%), *chronic* (=12.12%), *alcoholicg* (=0.10%), and *smokingg* (=0.40%) were higher than the 0.09% cutoff criterion, which indicated they were potential confounding variables.

Table 5 showed the associations between three co-occurring perceived pollution and QoL. The perceived air×noise×water pollution had significant associations with PCS scores (β = 12.119, 95%CI: 8.578, 15.659) and MCS scores (β = 11.216, 95%CI: 7.768, 14.665). *Hhdnum* had significant associations with PCS scores (β = 12.792, 95%CI: 12.056, 13.528) and MCS scores (β = 12.396, 95%CI: 11.682, 13.109).

**Table 5:** Associations between three co-occurring pollution and PCS and MCS scores, Coefficients (95%CI)

	PCS scores	MCS scores
Air×noise×water	12.119***(8.578,15.659)	11.216***(7.768,14.665)
Hhdnum	12.792***(12.056,13.528)	12.396***(11.682,13.109)
R-squared	0.784	0.780
Number of observation	3770	3770

Note: ***, ** and * indicates 1, 5 and 10% significance level, respectively. Hhdnum = number of household members

### Moderation analysis

The results in the potential models 1, 2, and 3 did not reach statistical significance in the supplementary Figs. 1–36. Thus, the simple moderation relationships were rejected. When mediators were dichotomous, the models 6, 7, and 8 couldn’t be used. Thus, one pollutant could not affect one of both PCS and MCS scores through another pollutant. The other potential models 9, 21, 22, and 70 also had no significant mediating outcomes. The *p*-values of interactions were not more than 0.10, the moderated mediation relationships were established. After bias-corrected 95% confidence interval (CI) with 1000 bootstrapping samples was calculated, Figs. 1 and 2 were identified the statistically according to model 10.

**Fig. 1:**
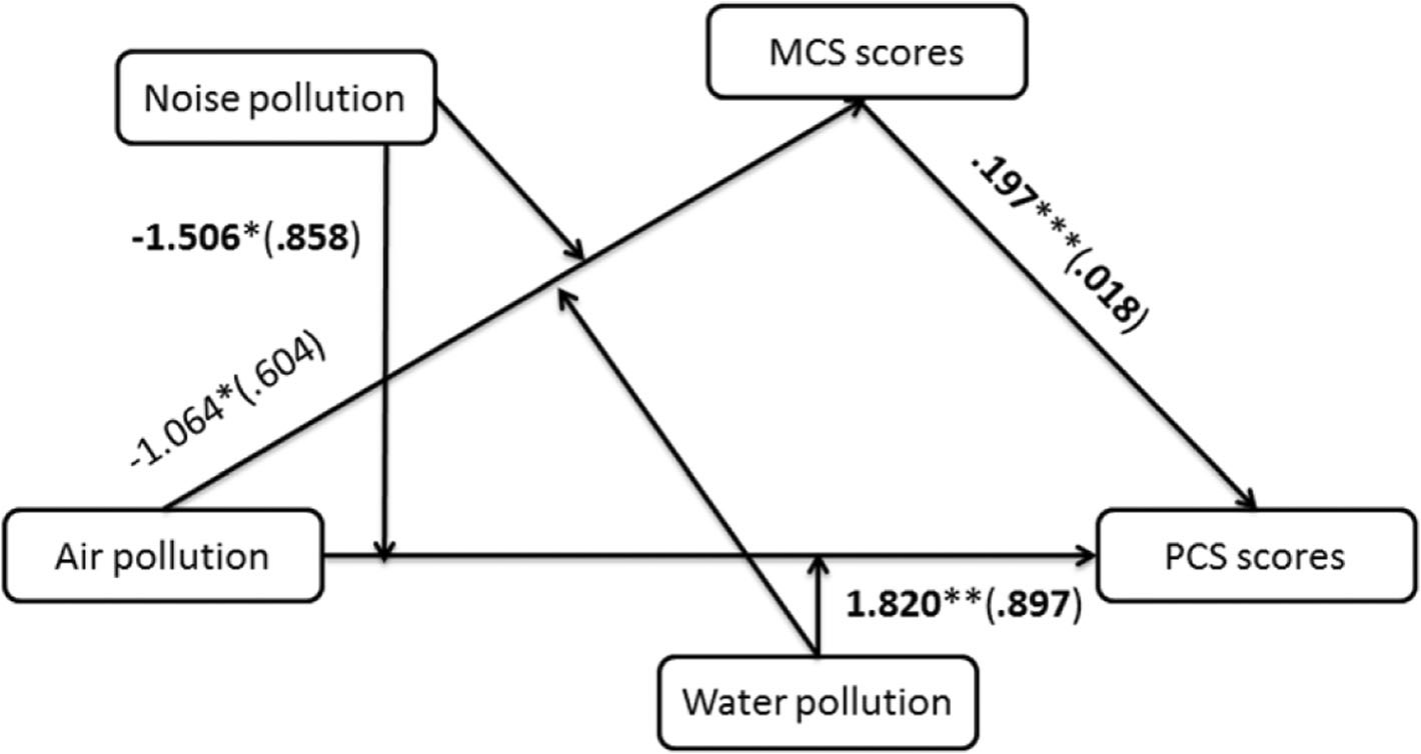
Moderated mediation model controlling for *employment*, *bmi*, *hhdnum*, *religion*, *alcoholicg*, and *smokingg* (*N* = 3697). Statistically significant path coefficients were indicated with asterisks (***, 1%; **, 5%; and *, 10%)

**Fig. 2:**
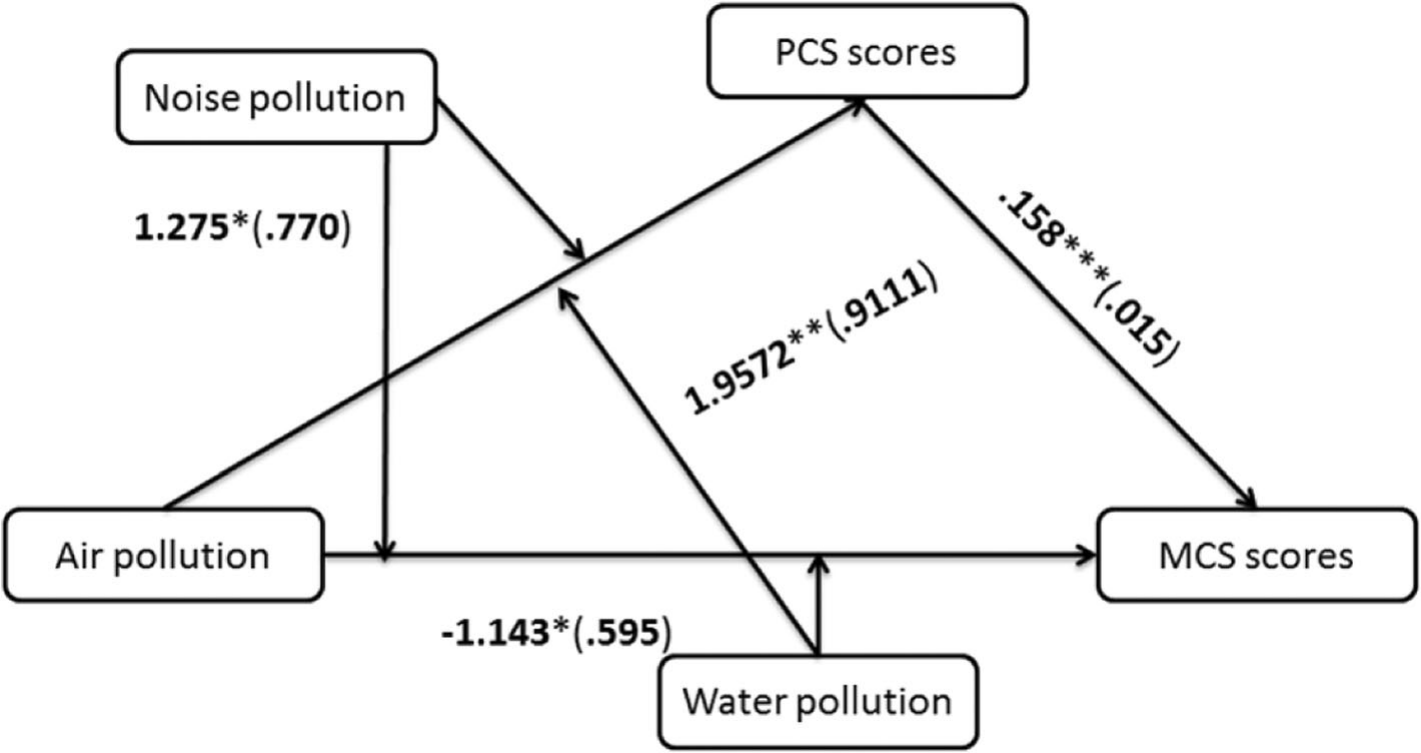
Moderated mediation model controlling for *employment*, *bmi*, *hhdnum*, *religion*, *alcoholicg*, and *smokingg* (*N* = 3697). Statistically significant path coefficients were indicated with asterisks (***, 1%; **, 5%; and *, 10%)

In Fig. 1, perceived air pollution had a negative association with MCS scores (β = − 1.064, *p* = .0783). MCS scores had a positive association with PCS scores (β = .197, *p* = .0000). Perceived water pollution had a negative association with PCS scores (β = − 1.742, *p* = .0048). Perceived air×water pollution had a positive association with PCS scores (β = 1.820, *p* = .0426). Perceived noise pollution had a positive association with PCS scores (β = 1.643, *p* = .0028). Perceived air×noise pollution had a negative association with PCS scores (β = − 1.506, *p* = .0794). Thus, there were no moderating effects and mediating effects of perceived air pollution on PCS scores.

In Fig. 2, perceived water pollution had a negative association with PCS scores (β = − 1.906, *p* = .0024). Perceived air×water pollution had a positive association with PCS scores (β = 1.957, *p* = .0318). Perceived noise pollution had a positive association with PCS scores (β = 1.545, *p* = .0057). PCS scores had a positive association with MCS scores (β = .158, *p* = .0000). Perceived air pollution had a negative association with MCS scores (β = − 1.143, *p* = .0548). Perceived air×noise pollution had a positive association with MCS scores (β = 1.275, *p* = .0978). Thus, there were no moderating effects and mediating effects of perceived air pollution on MCS scores.

## Discussion

On the novel use of the SF-12 in a Chinese adult population, this study reported the associations between perceived environmental pollution and PCS and MCS scores. Only considering air pollution, air pollution and water pollution had significant associations with PCS and MCS scores, respectively. Noise pollution had significant associations with PCS scores. The air×noise pollution, air×water pollution, and noise×water pollution had significant associations with PCS and MCS scores. The air×noise×water pollution had significant associations with PCS and MCS scores. Thus, it could be speculated that number of co-occurring perceived environmental pollution could increase PCS and MCS scores. Furthermore, this study highlighted the importance of socioeconomic factors (number of household members, religion, and employment) and lifestyle variables (body mass index, frequencies of smoking, and frequencies of alcoholic drinks) in the increasing PCS and MCS scores in this population.

It might be surprised that perceived environmental pollution could increase HRQoL on the basis of PCS and MCS scores in the general Chinese population. Especially, the outcomes in this study could be opposite of an early study that air pollution might worsen mental health in China [48]. An early study also reported there might be a link between exposure to air pollution and psychiatric disorders in children and adolescents [49]. In modern China, the common population often was cultivated with respect to cleanness and hygiene and educated the knowledge of environmental prevention. In order to fight against environmental degradation, China government controlled high pollution industries, demolished higher-polluting iron and steel works, and carried on strict policies on the discharge of harmful chemicals and compounds. Simultaneously, health authorities in China made efforts to reduce the long-term threats posed by environmental pollution. Chinese individuals were thus aware of the seriousness of environmental pollution and health risk in the neighborhood. Due to cognitive stress, they possibly overestimate their health risks of environmental pollution and carried out preventive countermeasures.

Prior studies in international circle could construct an evident chain to explain the fact in China’s setting. Several population-based questionnaire studies indicated perceived pollution and health risk perception played important roles in understanding and predicting environmentally induced symptoms and diseases [50, 51]. A mail-based questionnaire study concluded the capacity to process and perceived environmental health threats in a positive manner might be a valuable human ability positively influencing personal satisfaction and well-being [52]. Perceived risk of environmental threats often translated into psychological stress with a wide range of effects on health and well-being.

The findings here were in line with the prior worldwide research that documented the relationship between socioeconomic factors and lifestyle variables and HRQoL globally. Regarding number of household members, several studies reported that living arrangement was associated with higher risk of poor HRQoL [53, 54]. Considering the role of religion, Chen and Williams (2016) argued that the private and subjective dimension of religion mattered for well-being in China [55]. A growing body of peer-reviewed articles found that religious involvement was associated with QoL in old age [56], amongst older Ethiopians [57], and in patients with schizophrenia in Latin America [58].

With respect to employment status, the findings in this study were in line with the results in several early [59–62]. Clinically, employment was associated with QoL in multiple sclerosis patients [63], HRQoL among people with multiple sclerosis [64], HRQoL among people with schizophrenia [65], and HRQoL among Hungarian Hodgkin lymphoma survivors [66]. Thus, the associations could be generalized.

Regarding the association between BMI and HRQoL, the findings in this study were not in agreement with inverse association between BMI and physical HRQoL and positive association between BMI and mental HRQoL [67], inverse U-shaped association scores [68], and complex association [69]*.* Regarding the association between smoking and HRQoL, the findings in this study were not in agreement with inverse association [70–72]. Regarding the association between alcohol drinking and HRQoL, the findings in this study were in line with positive association between alcohol drinking and physical HRQoL [73], positive association between alcohol consumption and higher HRQoL in female [74]. The findings in this study were not in line with negative associations [75] in the early studies.

To my best knowledge, this is the first study to report the concurrent pollution rather than single pollution had associations with poor HRQoL. But, this study was not in agreement with the results from the prior studies due to different research designs. For example, air pollution and noise pollution had impact on health independently in North Island of New Zealand [76]. But, this study integrated two or three pollution into one. Similarly, this study split HRQoL into PCS and MCS instead of eight subdomains. Still, literature [76] was taken as an example. Without interactions between air pollution annoyance and noise annoyance, literature [76] indicated that air pollution annoyance and noise annoyance effectively predicted variability in the different HRQoL domains. This study also discorded with previous population-based studies conducted in other countries, in which HRQoL was considered as a covariant to predict perceptions of environmental pollution. Kamimura, et al. (2017) also used the East Asia data to conclude the integral HRQoL rather than sectional HRQoL was associated with levels of perceived environmental pollution [77]. Thus, this study enriched the knowledge of the relationship between pollution and HRQoL.

Exposure to environmental pollution was associated with the growing morbidity and mortality worldwide. Thus, some academic fruits pointed to the direction of governmental conceiving in China [78–80]. Due to high level of perceived environmental health threats, subjective PCS and MCS scores were high, and more preparedness possibly had occurred at the individual or household levels. This report had potential to inform the development of related policies and risk communication strategies in China. Thus, new immediate solutions should be conceived to curb the pervasive environmental problem.

The present study had several limitations. First, this study was conducted with missing data and without conducting imputation. Although the observations with missing data were a relatively small proportion of the eligible sample, the research outcomes were not reflected correct in the whole. Second, subjective assessments were adopted in a cross-sectional survey. Thus, due to lack of data on availability and accessibility of natural environments in the neighborhood, the role of before-after environmental epidemiology in the HRQoL could not be discovered. The subjective data also had limited abilities to allow causal study to be conducted among variables. Finally, since the some variables were controlled for, the current data framework might not reflect the demographic factors and HRQoL completely. Future analyses should make efforts to fill in the gaps.

## Conclusions

In conclusion, sociodemographic characteristics, lifestyle, and perceived environmental pollution were associated with PCS and MCS scores. The perceived single and co-occurring environmental pollution had significant associations with PCS and MCS scores in Chinese adult population. But, there were no moderating and mediating relationships among the perceived environmental pollutions. Further studies were to explore the causal effects of environmental pollution on PCS and MCS scores.

## Supplementary information

**Additional file 1: Supplementary Table 1.** Change-in-estimate for PCS scores with possible confounding factors. **Supplementary Table 2**. Change-in-estimate for MCS scores with possible confounding factors. **Supplementary Table 3.** Change-in-estimate for PCS scores with possible confounding factors. **Supplementary Table 4**. Change-in-estimate for MCS scores with possible confounding factors. **Supplementary Table 5.** Change-in-estimate for PCS scores with possible confounding factors. **Supplementary Table 6.** Change-in-estimate for MCS scores with possible confounding factors. **Supplementary Table 7**. Change-in-estimate for PCS scores with possible confounding factors. **Supplementary Table 8.** Change-in-estimate for MCS scores with possible confounding factors. **Supplementary Table 9**. Change-in-estimate for PCS scores with possible confounding factors. **Supplementary Table 10**. Change-in-estimate for MCS scores with possible confounding factors. **Supplementary Table 11**. Change-in-estimate for PCS scores with possible confounding factors. **Supplementary Table 12**. Change-in-estimate for MCS scores with possible confounding factors. **Supplementary Table 13**. Change-in-estimate for PCS scores with possible confounding factors. **Supplementary Table 14.** Change-in-estimate for MCS scores with possible confounding factors.

**Additional file 2 **Potential conceptual diagrams.

**Additional file 3 **Statistical outcomes of potential conceptual diagrams.

## Data Availability

http://www.eassda.org/

## Abbreviations

HRQoL: Health-related quality of life
MCS: Mental component summary
PCS: Physical component summary
PF: Physical functioning
RP: Role physical
BP: Bodily pain
GH: General health
VT: Vitality
SF: Functioning
RE: Role Emotional
MH: Mental health
BMI: Body mass index
AOR: Adjusted odds ratio
