# Supplementary material for: Associations between perceived environmental pollution and health-related quality of life in a Chinese adult population
Source: Health Qual Life Outcomes. 2020 Jun 23;18:198. doi: 10.1186/s12955-020-01442-9 (PMC7310336; doi:10.1186/s12955-020-01442-9)
Supplement: Supplementary file 1 — Additional file 1: Supplementary Table 1. Change-in-estimate for PCS scores with possible confounding factors. Supplementary Table 2. Change-in-estimate for MCS scores with possible confounding factors. Supplementary Table 3. Change-in-estimate for PCS scores with possible confounding factors. Supplementary Table 4. Change-in-estimate for MCS scores with possible confounding factors. Supplementary Table 5. Change-in-estimate for PCS scores with possible confounding factors. Supplementary Table 6. Change-in-estimate for MCS scores with possible confounding factors. Supplementary Table 7. Change-in-estimate for PCS scores with possible confounding factors. Supplementary Table 8. Change-in-estimate for MCS scores with possible confounding factors. Supplementary Table 9. Change-in-estimate for PCS scores with possible confounding factors. Supplementary Table 10. Change-in-estimate for MCS scores with possible confounding factors. Supplementary Table 11. Change-in-estimate for PCS scores with possible confounding factors. Supplementary Table 12. Change-in-estimate for MCS scores with possible confounding factors. Supplementary Table 13. Change-in-estimate for PCS scores with possible confounding factors. Supplementary Table 14. Change-in-estimate for MCS scores with possible confounding factors. [file 12955_2020_1442_MOESM1_ESM.doc]

Supplementary Table 1. Change-in-estimate for PCS scores with possible confounding factors.

| Variables removed | Odds ratio | [95% Conf. Interval] | Change, % |
| --- | --- | --- | --- |
| Adj.All | 0.6854 | 0.3273-1.4354 |  |
| **-hhdnum** | **0.6853** | **0.3278-1.4328** | **0.0195** |
| -religion | 0.6844 | 0.3267-1.4335 | 0.1315 |
| -alcoholicg | 0.6831 | 0.3266-1.4288 | 0.1900 |
| -cn_hinc | 0.6810 | 0.3256-1.4242 | 0.3093 |
| -sex | 0.6753 | 0.3243-1.4065 | 0.8285 |
| **-smokingg** | **0.6753** | **0.3226-1.4137** | **0.0099** |
| -employment | 0.6807 | 0.3217-1.4403 | 0.7998 |
| -maritaln | 0.6928 | 0.3271-1.4671 | 1.7795 |
| -age | 0.7157 | 0.3242-1.5800 | 3.3044 |
| -urbruraln | 0.7207 | 0.3363-1.5446 | 0.6997 |
| -bmi | 0.7671 | 0.3585-1.6414 | 6.4469 |
| -chronic | 0.4900 | 0.2029-1.1830 | 36.1305 |
| -educyrs | 1.3719 | 0.5505-3.4187 | 179.9964 |

Supplementary Table 2. Change-in-estimate for MCS scores with possible confounding factors.

| Variables removed | Odds ratio | [95% Conf. Interval] | Change, % |
| --- | --- | --- | --- |
| Adj.All | 0.2722 | 0.1176-0.6301 |  |
| **-smokingg** | **0.2721** | **0.1176-0.6295** | **0.0545** |
| -alcoholicg | 0.2716 | 0.1175-0.6281 | 0.1558 |
| -religion | 0.2724 | 0.1181-0.6285 | 0.2793 |
| -hhdnum | 0.2742 | 0.1183-0.6355 | 0.6782 |
| -age | 0.2728 | 0.1179-0.6315 | 0.5098 |
| -employment | 0.2742 | 0.1183-0.6353 | 0.4911 |
| -maritaln | 0.2773 | 0.1198-0.6421 | 1.1555 |
| -cn_hinc | 0.2720 | 0.1174-0.6301 | 1.9355 |
| -sex | 0.2632 | 0.1139-0.6080 | 3.2276 |
| -bmi | 0.2846 | 0.1227-0.6599 | 8.1111 |
| -educyrs | 0.3159 | 0.1352-0.7385 | 11.0324 |
| -chronic | 0.2815 | 0.1170-0.6768 | 10.9129 |
| -urbruraln | 0.4766 | 0.2052-1.1067 | 69.3269 |

Supplementary Table 3. Change-in-estimate for PCS scores with possible confounding factors.

| Variables removed | Odds ratio | [95% Conf. Interval] | Change, % |
| --- | --- | --- | --- |
| Adj.All | 0.6631 | 0.3297-1.3337 |  |
| **-hhdnum** | **0.6633** | **0.3300-1.3329** | **0.0210** |
| -cn_hinc | 0.6627 | 0.3297-1.3318 | 0.0901 |
| -religion | 0.6620 | 0.3290-1.3321 | 0.1049 |
| **-bmi** | **0.6620** | **0.3281-1.3354** | **0.0000** |
| -alcoholicg | 0.6593 | 0.3268-1.3301 | 0.4049 |
| -maritaln | 0.6625 | 0.3280-1.3378 | 0.4817 |
| -sex | 0.6667 | 0.3304-1.3454 | 0.6354 |
| -age | 0.6754 | 0.3292-1.3855 | 1.3050 |
| -smokingg | 0.7000 | 0.3405-1.4390 | 3.6444 |
| -employment | 0.6662 | 0.3214-1.3809 | 4.8244 |
| -urbruraln | 0.6955 | 0.3360-1.4399 | 4.3993 |
| -chronic | 0.3682 | 0.1545-0.8773 | 47.0650 |
| -educyrs | 1.7022 | 0.6919-4.1878 | 362.3407 |

Supplementary Table 4. Change-in-estimate for MCS scores with possible confounding factors.

| Variables removed | Odds ratio | [95% Conf. Interval] | Change, % |
| --- | --- | --- | --- |
| Adj.All | 0.3867 | 0.1719-0.8697 |  |
| **-smokingg** | **0.3865** | **0.1718-0.8693** | **0.0508** |
| **-bmi** | **0.3862** | **0.1715-0.8699** | **0.0692** |
| -maritaln | 0.3866 | 0.1711-0.8733 | 0.0974 |
| -alcoholicg | 0.3857 | 0.1709-0.8702 | 0.2316 |
| -hhdnum | 0.3848 | 0.1706-0.8677 | 0.2426 |
| -religion | 0.3868 | 0.1715-0.8724 | 0.5219 |
| -cn_hinc | 0.3843 | 0.1702-0.8677 | 0.6419 |
| -age | 0.3793 | 0.1675-0.8589 | 1.2981 |
| -employment | 0.3751 | 0.1658-0.8486 | 1.1174 |
| -sex | 0.3912 | 0.1720-0.8902 | 4.3169 |
| -chronic | 0.3085 | 0.1323-0.7193 | 21.1382 |
| -urbruraln | 0.3857 | 0.1696-0.8772 | 25.0116 |
| -educyrs | 0.8032 | 0.3544-1.8202 | 108.2362 |

Supplementary Table 5. Change-in-estimate for PCS scores with possible confounding factors.

| Variables removed | Odds ratio | [95% Conf. Interval] | Change, % |
| --- | --- | --- | --- |
| Adj.All | 0.3925 | 0.1854-0.8307 |  |
| **-religion** | **0.3927** | **0.1857-0.8306** | **0.0548** |
| -hhdnum | 0.3933 | 0.1853-0.8347 | 0.1426 |
| -alcoholicg | 0.3927 | 0.1854-0.8316 | 0.1472 |
| -cn_hinc | 0.3916 | 0.1850-0.8290 | 0.2759 |
| -sex | 0.3956 | 0.1867-0.8383 | 1.0319 |
| -smokingg | 0.3917 | 0.1844-0.8323 | 0.9890 |
| -bmi | 0.3995 | 0.1873-0.8522 | 1.9895 |
| -age | 0.4100 | 0.1871-0.8982 | 2.6116 |
| -maritaln | 0.4018 | 0.1837-0.8788 | 1.9918 |
| -educyrs | 0.3776 | 0.1718-0.8298 | 6.0323 |
| -employment | 0.4012 | 0.1771-0.9091 | 6.2643 |
| -chronic | 0.3175 | 0.1183-0.8521 | 20.8671 |
| -urbruraln | 0.4215 | 0.1568-1.1334 | 32.7604 |

Supplementary Table 6. Change-in-estimate for MCS scores with possible confounding factors.

| Variables removed | Odds ratio | [95% Conf. Interval] | Change, % |
| --- | --- | --- | --- |
| Adj.All | 0.3268 | 0.1409-0.7581 |  |
| **-religion** | **0.3268** | **0.1409-0.7580** | **0.0163** |
| -alcoholicg | 0.3265 | 0.1409-0.7569 | 0.0954 |
| -smokingg | 0.3273 | 0.1412-0.7586 | 0.2425 |
| -age | 0.3239 | 0.1399-0.7501 | 1.0324 |
| -employment | 0.3275 | 0.1412-0.7594 | 1.0835 |
| -educyrs | 0.3226 | 0.1388-0.7496 | 1.4852 |
| -bmi | 0.3284 | 0.1408-0.7662 | 1.8140 |
| -cn_hinc | 0.3199 | 0.1368-0.7477 | 2.6113 |
| -hhdnum | 0.3107 | 0.1317-0.7330 | 2.8543 |
| -maritaln | 0.3021 | 0.1279-0.7132 | 2.7855 |
| -sex | 0.3151 | 0.1331-0.7460 | 4.3004 |
| -chronic | 0.2862 | 0.1178-0.6955 | 9.1663 |
| -urbruraln | 0.3518 | 0.1454-0.8512 | 22.9221 |

Supplementary Table 7. Change-in-estimate for PCS scores with possible confounding factors.

| Variables removed | Odds ratio | [95% Conf. Interval] | Change, % |
| --- | --- | --- | --- |
| Adj.All | 0.5859 | 0.2604-1.3181 |  |
| **-hhdnum** | **0.5856** | **0.2599-1.3195** | **0.0404** |
| -religion | 0.5849 | 0.2592-1.3196 | 0.1323 |
| -cn_hinc | 0.5839 | 0.2588-1.3176 | 0.1547 |
| -sex | 0.5822 | 0.2580-1.3136 | 0.2971 |
| -alcoholicg | 0.5815 | 0.2578-1.3117 | 0.1164 |
| -age | 0.5773 | 0.2498-1.3339 | 0.7306 |
| -maritaln | 0.5746 | 0.2490-1.3260 | 0.4668 |
| -smokingg | 0.5693 | 0.2457-1.3188 | 0.9255 |
| -employment | 0.5741 | 0.2448-1.3464 | 0.8398 |
| -urbruraln | 0.5816 | 0.2514-1.3456 | 1.3109 |
| -bmi | 0.6260 | 0.2702-1.4506 | 7.6437 |
| -chronic | 0.3057 | 0.1124-0.8317 | 51.1613 |
| -educyrs | 1.1821 | 0.4198-3.3286 | 286.6229 |

Supplementary Table 8. Change-in-estimate for MCS scores with possible confounding factors.

| Variables removed | Odds ratio | [95% Conf. Interval] | Change, % |
| --- | --- | --- | --- |
| Adj.All | 0.4574 | 0.1808-1.1570 |  |
| **-smokingg** | **0.4577** | **0.1810-1.1573** | **0.0762** |
| -religion | 0.4583 | 0.1813-1.1587 | 0.1292 |
| -alcoholicg | 0.4575 | 0.1811-1.1562 | 0.1730 |
| -employment | 0.4589 | 0.1820-1.1568 | 0.2866 |
| -maritaln | 0.4573 | 0.1810-1.1552 | 0.3394 |
| -sex | 0.4555 | 0.1796-1.1558 | 0.3828 |
| -age | 0.4516 | 0.1775-1.1492 | 0.8642 |
| -cn_hinc | 0.4472 | 0.1754-1.1401 | 0.9726 |
| -hhdnum | 0.4549 | 0.1778-1.1634 | 1.7104 |
| -bmi | 0.4977 | 0.1942-1.2756 | 9.4226 |
| -educyrs | 0.5846 | 0.2269-1.5062 | 17.4488 |
| -chronic | 0.4779 | 0.1784-1.2804 | 18.2444 |
| -urbruraln | 0.8658 | 0.3348-2.2389 | 81.1531 |

Supplementary Table 9. Change-in-estimate for PCS scores with possible confounding factors.

| Variables removed | Odds ratio | [95% Conf. Interval] | Change, % |
| --- | --- | --- | --- |
| Adj.All | 0.6029 | 0.2544-1.4292 |  |
| **-alcoholicg** | **0.6028** | **0.2548-1.4261** | **0.0225** |
| **-religion** | **0.6023** | **0.2543-1.4268** | **0.0765** |
| **-hhdnum** | **0.6028** | **0.2540-1.4305** | **0.0764** |
| -bmi | 0.6010 | 0.2510-1.4390 | 0.3067 |
| -cn_hinc | 0.5987 | 0.2502-1.4324 | 0.3814 |
| -maritaln | 0.5952 | 0.2489-1.4232 | 0.5776 |
| -sex | 0.5991 | 0.2510-1.4301 | 0.6509 |
| -employment | 0.6108 | 0.2519-1.4810 | 1.9575 |
| -educyrs | 0.6240 | 0.2586-1.5057 | 2.1569 |
| -smokingg | 0.6059 | 0.2474-1.4843 | 2.8938 |
| -chronic | 0.5643 | 0.2063-1.5436 | 6.8746 |
| -age | 0.6538 | 0.2149-1.9894 | 15.8704 |
| -urbruraln | 1.0485 | 0.3472-3.1665 | 60.3680 |

Supplementary Table 10. Change-in-estimate for MCS scores with possible confounding factors.

| Variables removed | Odds ratio | [95% Conf. Interval] | Change, % |
| --- | --- | --- | --- |
| Adj.All | 0.2500 | 0.0940-0.6651 |  |
| **-alcoholicg** | **0.2500** | **0.0940-0.6647** | **0.0118** |
| **-religion** | **0.2501** | **0.0941-0.6644** | **0.0250** |
| -bmi | 0.2494 | 0.0941-0.6610 | 0.2629 |
| -smokingg | 0.2499 | 0.0943-0.6623 | 0.2069 |
| -maritaln | 0.2484 | 0.0938-0.6582 | 0.6088 |
| -hhdnum | 0.2469 | 0.0924-0.6600 | 0.5928 |
| -employment | 0.2489 | 0.0928-0.6675 | 0.7823 |
| -sex | 0.2516 | 0.0938-0.6749 | 1.0931 |
| -age | 0.2455 | 0.0923-0.6530 | 2.4429 |
| -chronic | 0.2397 | 0.0874-0.6573 | 2.3586 |
| -cn_hinc | 0.2342 | 0.0853-0.6428 | 2.2734 |
| -educyrs | 0.2439 | 0.0878-0.6775 | 4.1385 |
| -urbruraln | 0.3490 | 0.1275-0.9553 | 43.0886 |

Supplementary Table 11. Change-in-estimate for PCS scores with possible confounding factors.

| Variables removed | Odds ratio | [95% Conf. Interval] | Change, % |
| --- | --- | --- | --- |
| Adj.All | 0.5660 | 0.2379-1.3466 |  |
| **-hhdnum** | **0.5662** | **0.2382-1.3457** | **0.0341** |
| -religion | 0.5653 | 0.2376-1.3449 | 0.1558 |
| -cn_hinc | 0.5638 | 0.2370-1.3412 | 0.2681 |
| -sex | 0.5659 | 0.2381-1.3447 | 0.3712 |
| -alcoholicg | 0.5635 | 0.2374-1.3375 | 0.4070 |
| **-employment** | **0.5633** | **0.2360-1.3449** | **0.0413** |
| -bmi | 0.5667 | 0.2366-1.3571 | 0.5955 |
| -maritaln | 0.5549 | 0.2317-1.3293 | 2.0712 |
| -smokingg | 0.5314 | 0.2208-1.2792 | 4.2348 |
| -educyrs | 0.5676 | 0.2344-1.3744 | 6.8051 |
| -chronic | 0.4359 | 0.1541-1.2328 | 23.2020 |
| -age | 0.6026 | 0.1940-1.8722 | 38.2498 |
| -urbruraln | 1.1317 | 0.3661-3.4981 | 87.7915 |

Supplementary Table 12. Change-in-estimate for MCS scores with possible confounding factors.

| Variables removed | Odds ratio | [95% Conf. Interval] | Change, % |
| --- | --- | --- | --- |
| Adj.All | 0.2566 | 0.0910-0.7234 |  |
| -religion | 0.2570 | 0.0912-0.7243 | 0.1464 |
| -employment | 0.2564 | 0.0911-0.7216 | 0.2080 |
| **-smokingg** | **0.2564** | **0.0913-0.7197** | **0.0128** |
| -alcoholicg | 0.2559 | 0.0912-0.7178 | 0.1833 |
| -hhdnum | 0.2552 | 0.0908-0.7168 | 0.2958 |
| -bmi | 0.2561 | 0.0910-0.7211 | 0.3806 |
| -sex | 0.2587 | 0.0911-0.7349 | 1.0086 |
| -maritaln | 0.2551 | 0.0895-0.7270 | 1.4056 |
| -cn_hinc | 0.2505 | 0.0877-0.7154 | 1.8066 |
| -age | 0.2380 | 0.0828-0.6843 | 4.9805 |
| -educyrs | 0.2571 | 0.0888-0.7443 | 8.0327 |
| -chronic | 0.2397 | 0.0796-0.7220 | 6.7739 |
| -urbruraln | 0.3874 | 0.1319-1.1380 | 61.6300 |

Supplementary Table 13.Change-in-estimate for PCS scores with possible confounding factors.

| Variables removed | Odds ratio | [95% Conf. Interval] | Change, % |
| --- | --- | --- | --- |
| Adj.All | 0.6204 | 0.2439-1.5784 |  |
| **-hhdnum** | **0.6204** | **0.2439-1.5784** | **0.0018** |
| -religion | 0.6194 | 0.2433-1.5774 | 0.1620 |
| -alcoholicg | 0.6180 | 0.2431-1.5715 | 0.2269 |
| -cn_hinc | 0.6163 | 0.2424-1.5667 | 0.2871 |
| -bmi | 0.6191 | 0.2423-1.5818 | 0.4549 |
| -sex | 0.6265 | 0.2455-1.5987 | 1.1961 |
| -maritaln | 0.6137 | 0.2399-1.5696 | 2.0434 |
| -employment | 0.6257 | 0.2426-1.6135 | 1.9567 |
| -smokingg | 0.5882 | 0.2266-1.5271 | 5.9863 |
| -age | 0.6422 | 0.2439-1.6908 | 9.1749 |
| -urbruraln | 0.6582 | 0.2521-1.7187 | 2.4899 |
| -chronic | 0.3990 | 0.1222-1.3030 | 39.3724 |
| -educyrs | 1.1851 | 0.3499-4.0134 | 196.9925 |

Supplementary Table 14.Change-in-estimate for MCS scores with possible confounding factors.

| Variables removed | Odds ratio | [95% Conf. Interval] | Change, % |
| --- | --- | --- | --- |
| Adj.All | 0.4650 | 0.1584-1.3648 |  |
| **-hhdnum** | **0.4649** | **0.1580-1.3681** | **0.0284** |
| -alcoholicg | 0.4644 | 0.1579-1.3659 | 0.0962 |
| -religion | 0.4657 | 0.1583-1.3700 | 0.2702 |
| -smokingg | 0.4676 | 0.1593-1.3721 | 0.4029 |
| -bmi | 0.4695 | 0.1610-1.3687 | 0.4072 |
| -employment | 0.4721 | 0.1624-1.3726 | 0.5555 |
| -sex | 0.4774 | 0.1631-1.3976 | 1.1355 |
| -maritaln | 0.4703 | 0.1601-1.3817 | 1.5008 |
| -cn_hinc | 0.4613 | 0.1566-1.3594 | 1.8976 |
| -age | 0.4442 | 0.1503-1.3124 | 3.7187 |
| -educyrs | 0.4990 | 0.1683-1.4796 | 12.3348 |
| -chronic | 0.4385 | 0.1422-1.3526 | 12.1178 |
| -urbruraln | 0.7110 | 0.2367-2.1359 | 62.1451 |
