# Supplementary material for: Associations between perceived environmental pollution and health-related quality of life in a Chinese adult population
Source: Health Qual Life Outcomes. 2020 Jun 23;18:198. doi: 10.1186/s12955-020-01442-9 (PMC7310336; doi:10.1186/s12955-020-01442-9)
Supplement: Supplementary file 2 — Additional file 2 Potential conceptual diagrams. [file 12955_2020_1442_MOESM2_ESM.docx]

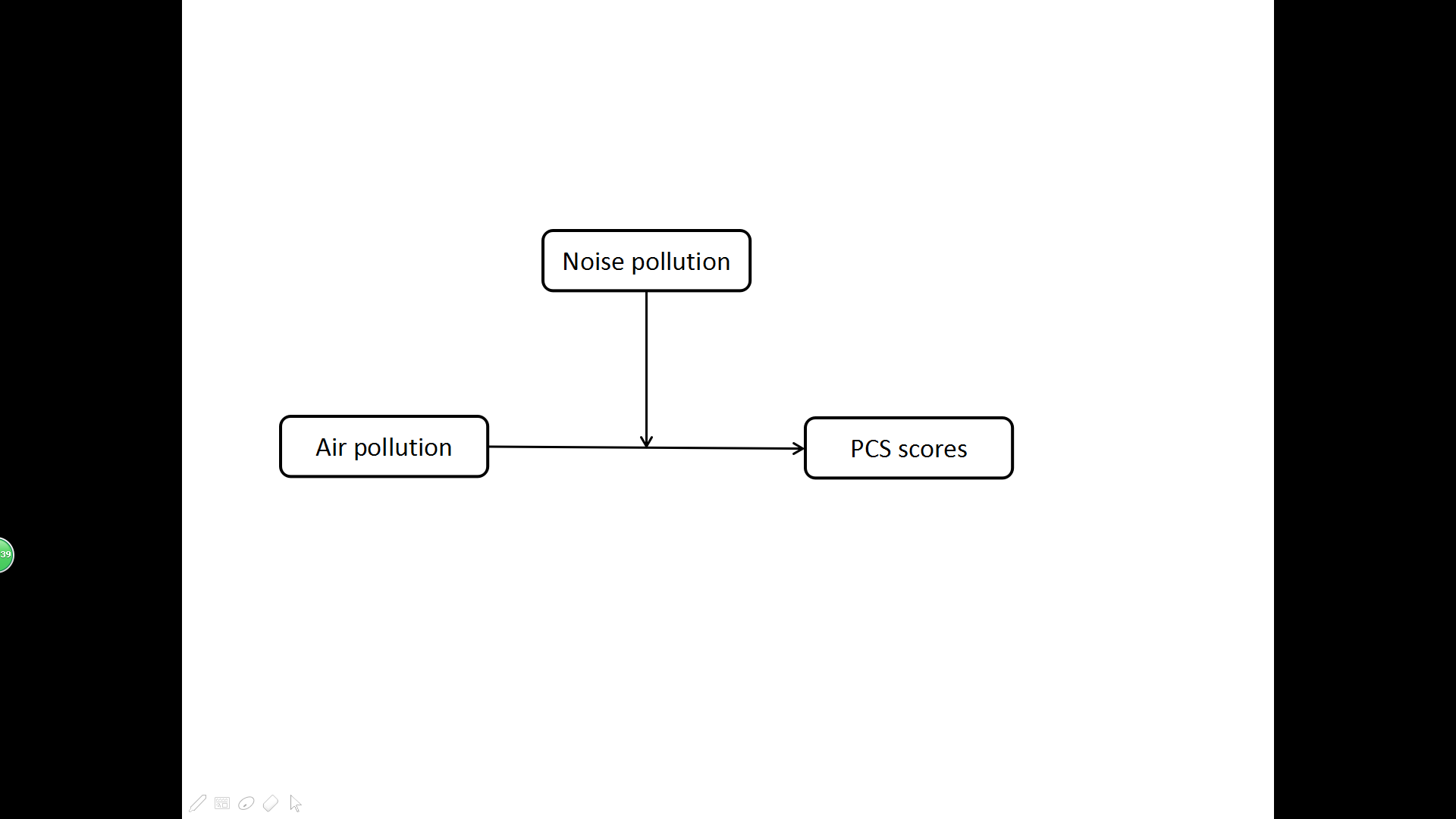

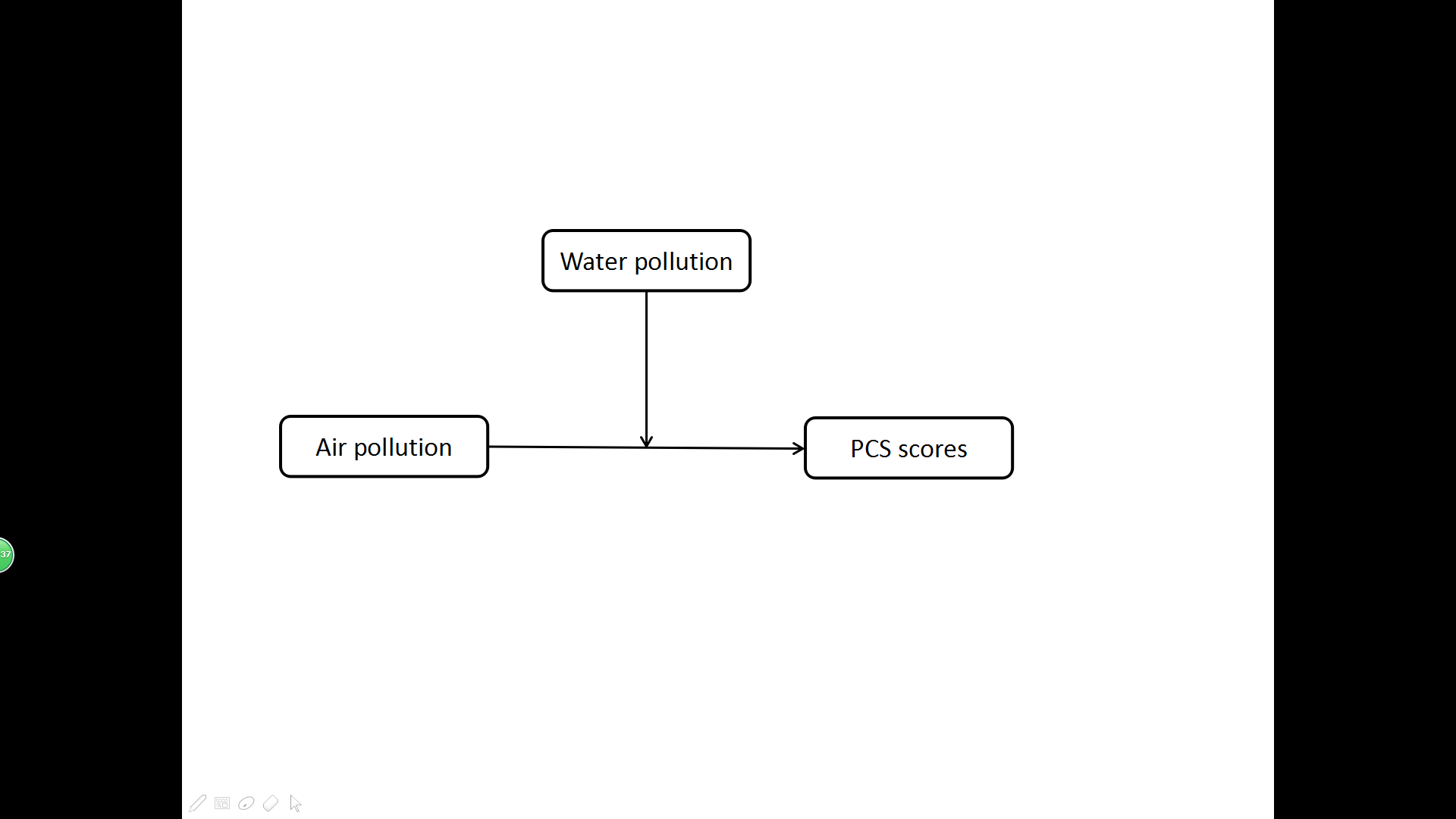


Figure 1. Potential conceptual diagram 1. Figure 2. Potential conceptual diagram 2.


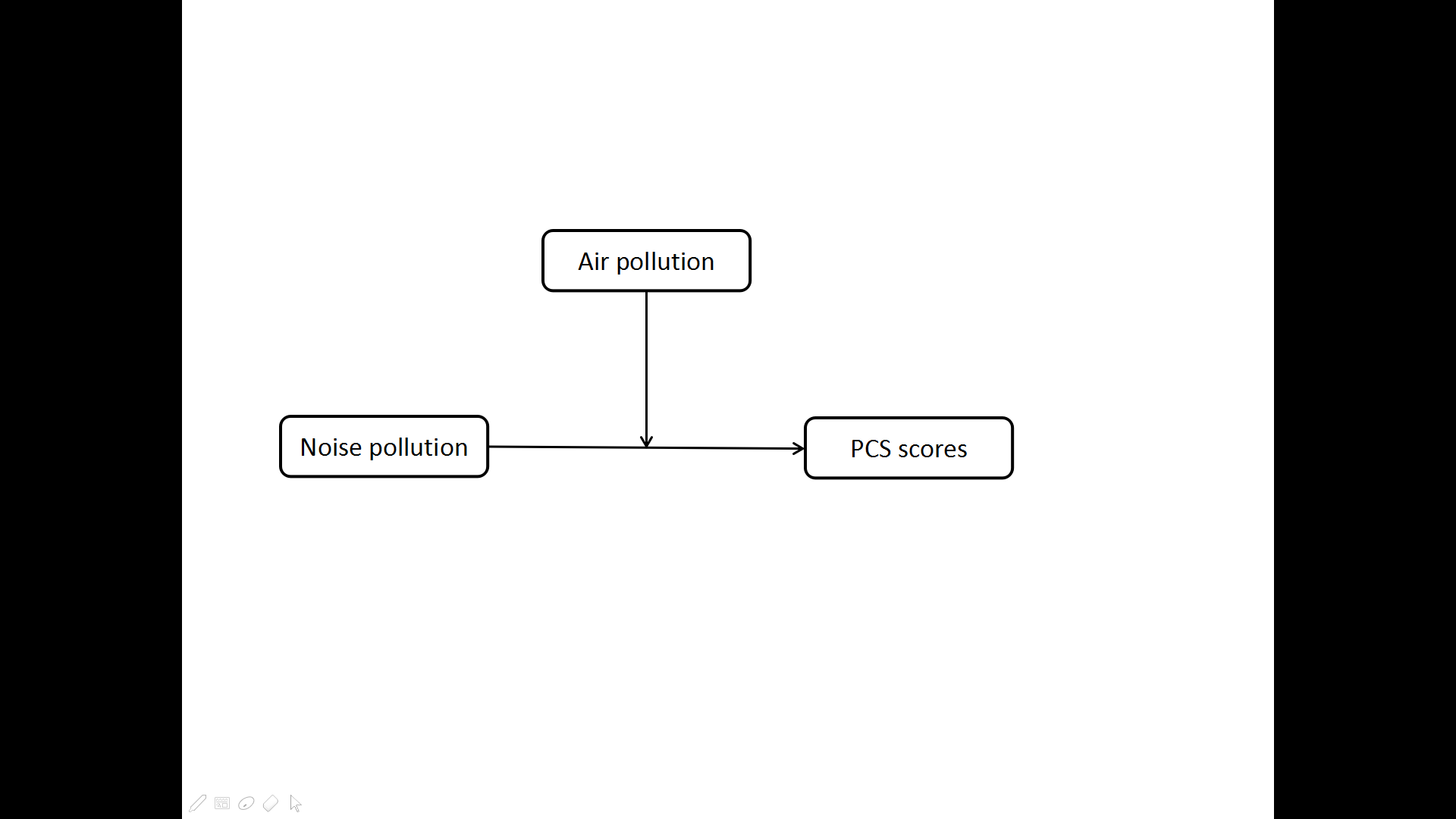

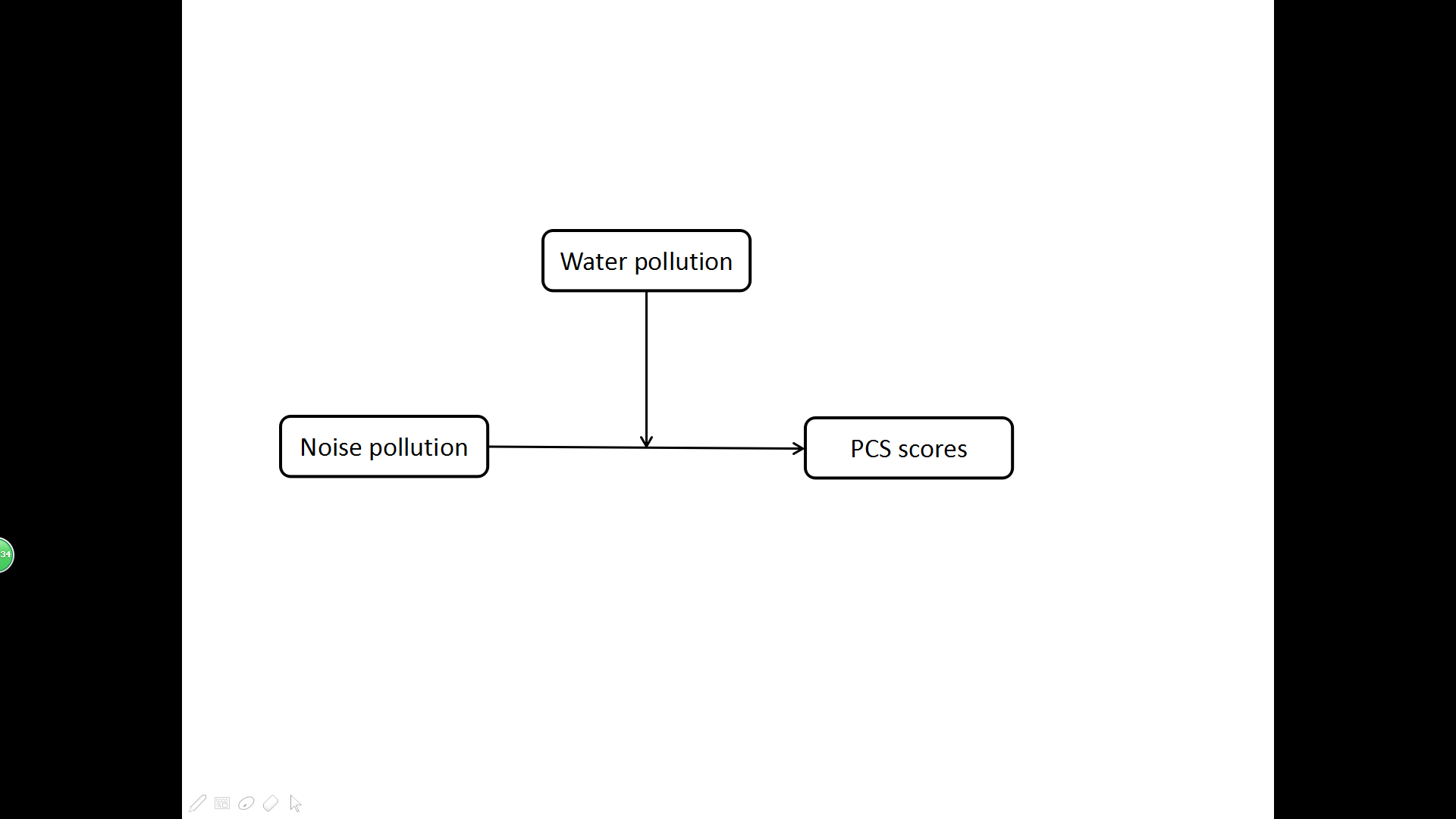


Figure 3. Potential conceptual diagram 3. Figure 4. Potential conceptual diagram 4.


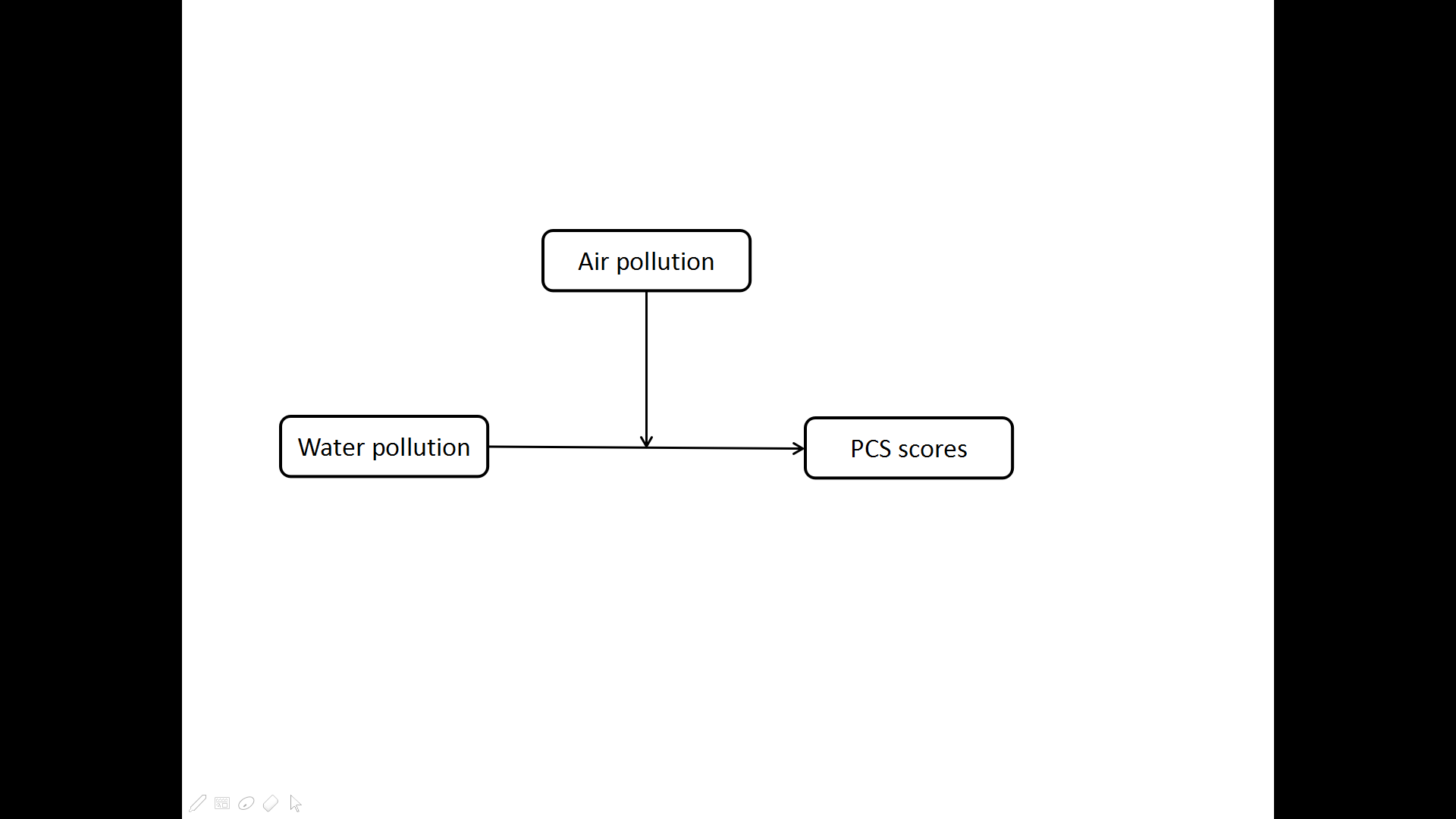

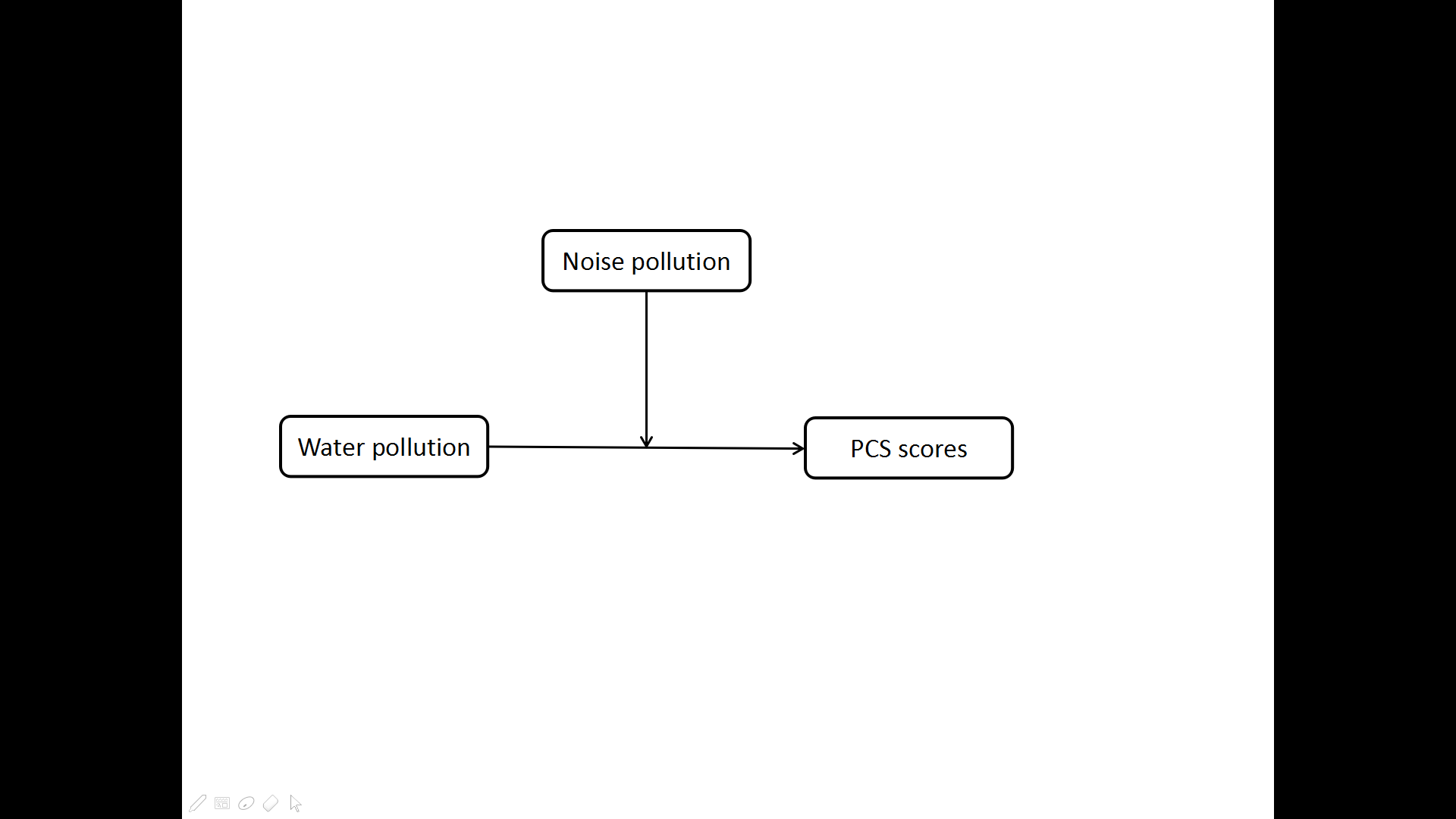


Figure 5. Potential conceptual diagram 5. Figure 6. Potential conceptual diagram 6.


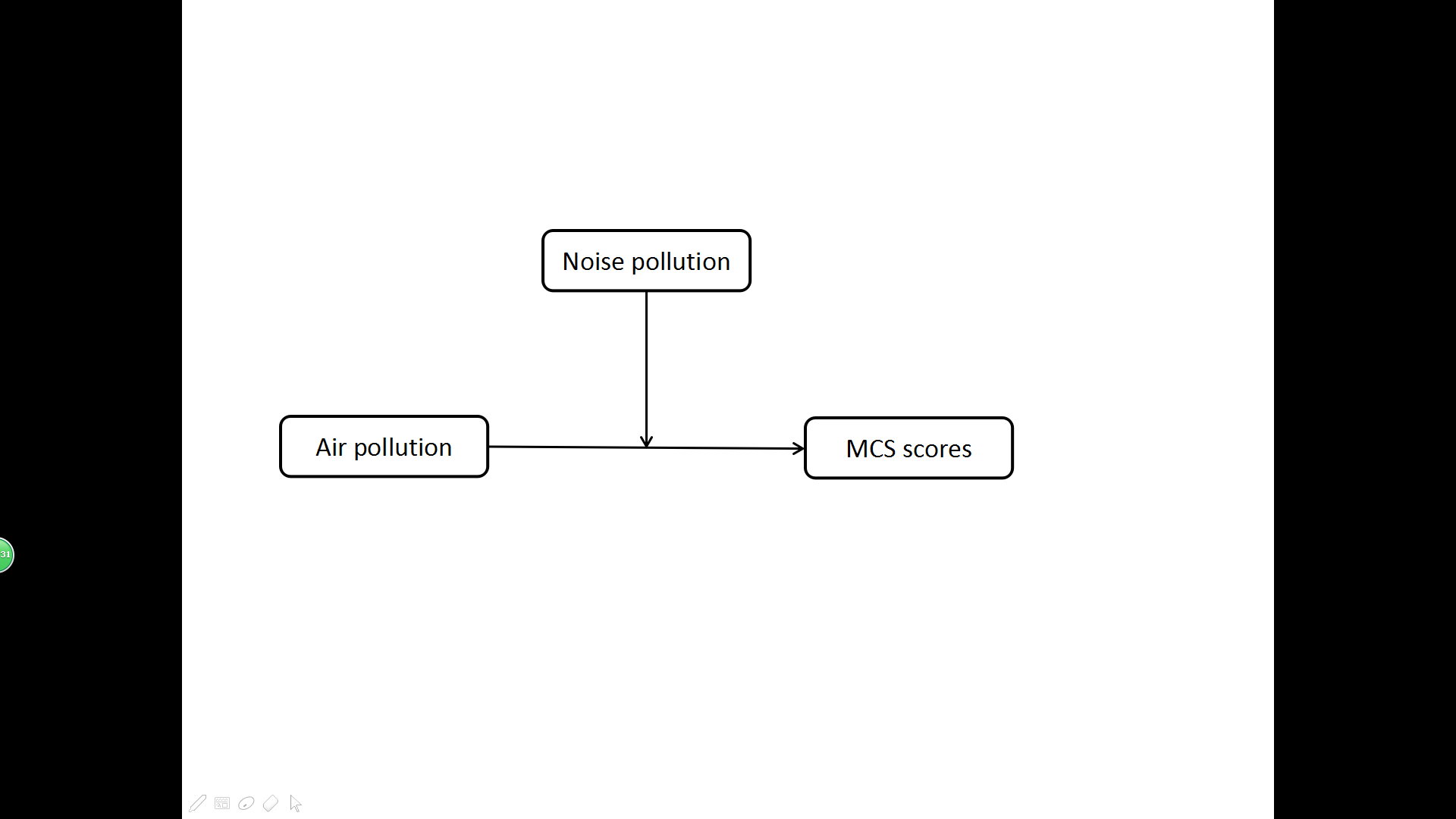

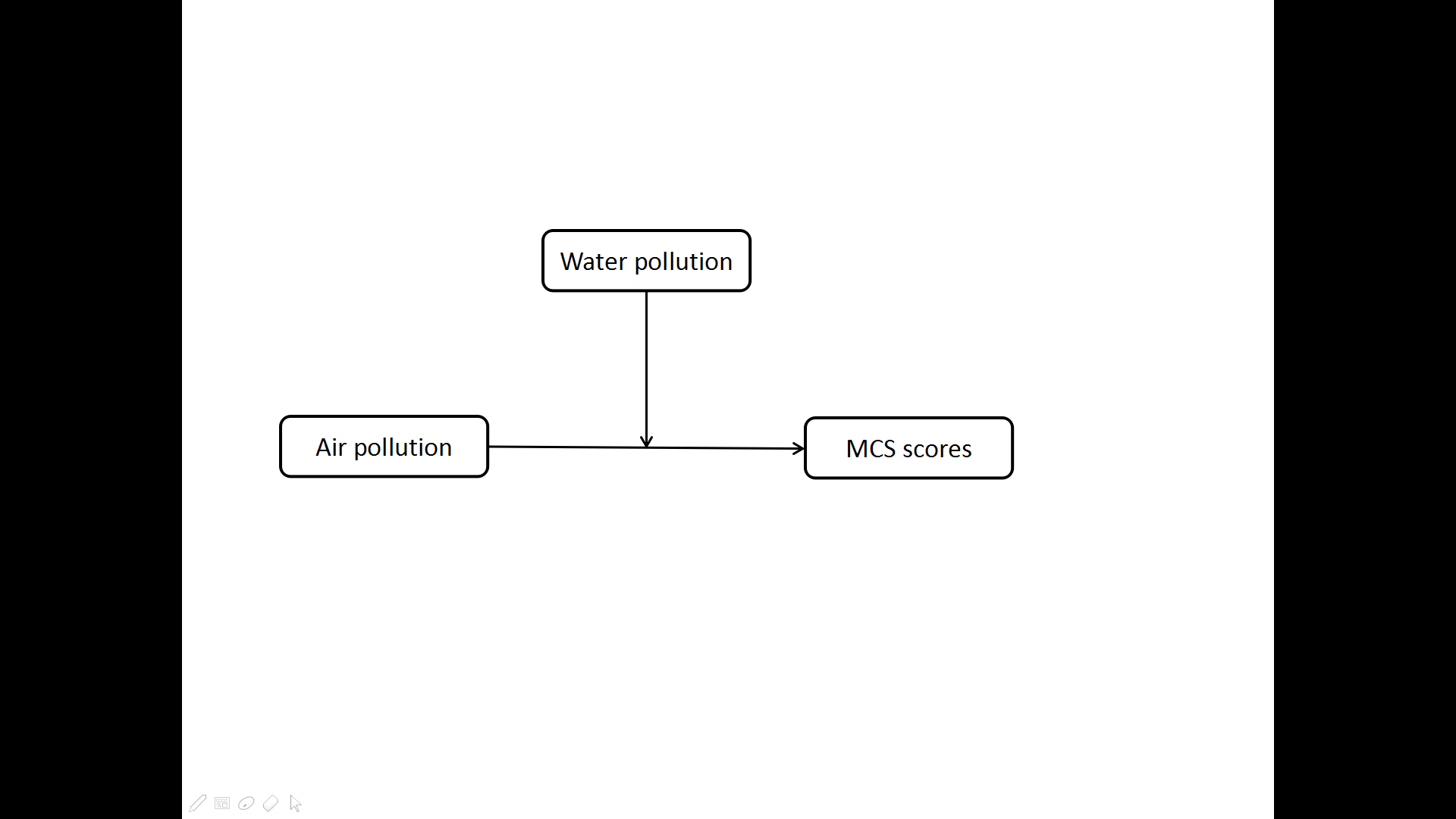


Figure 7. Potential conceptual diagram 7. Figure 8. Potential conceptual diagram 8.


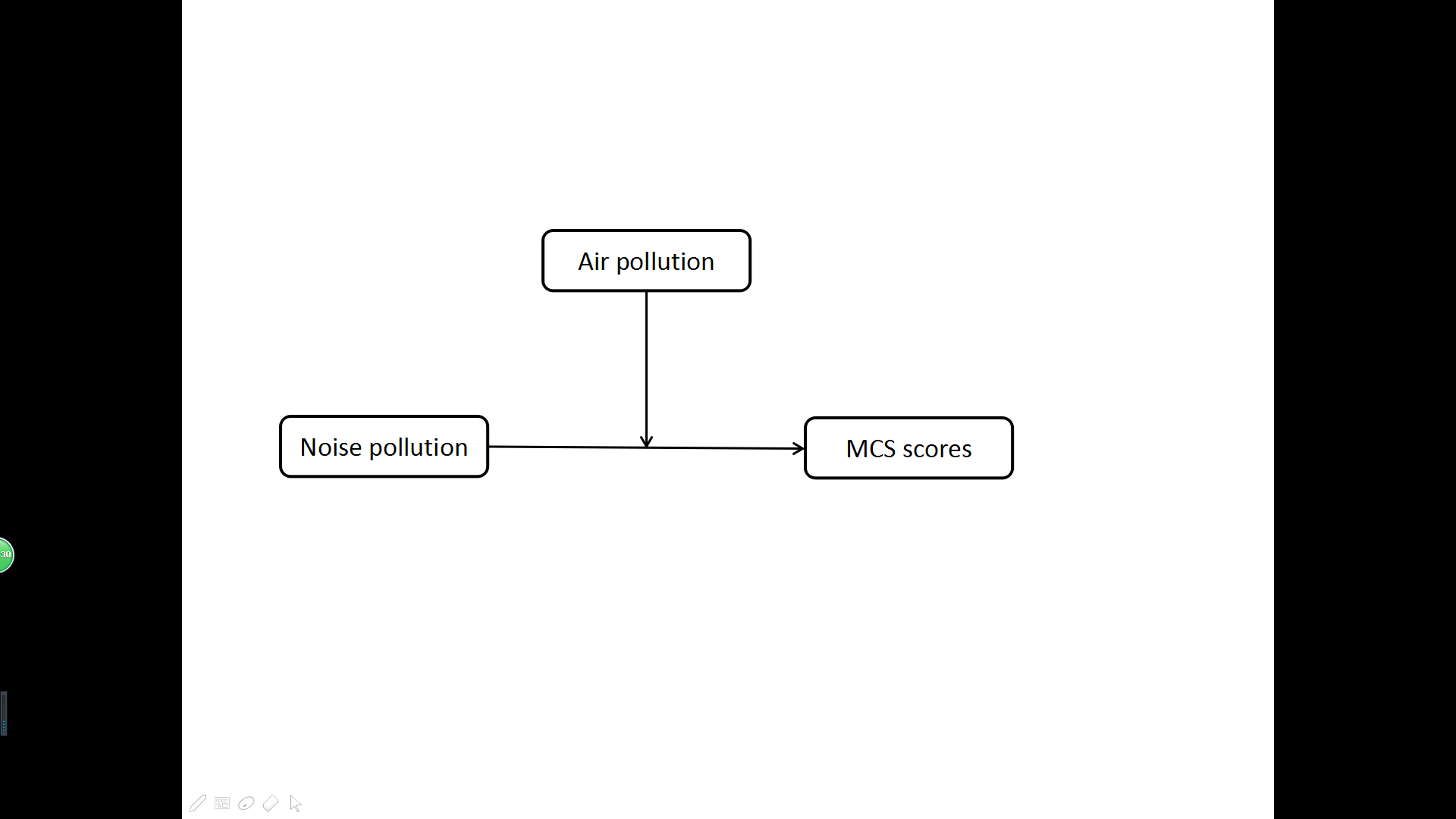

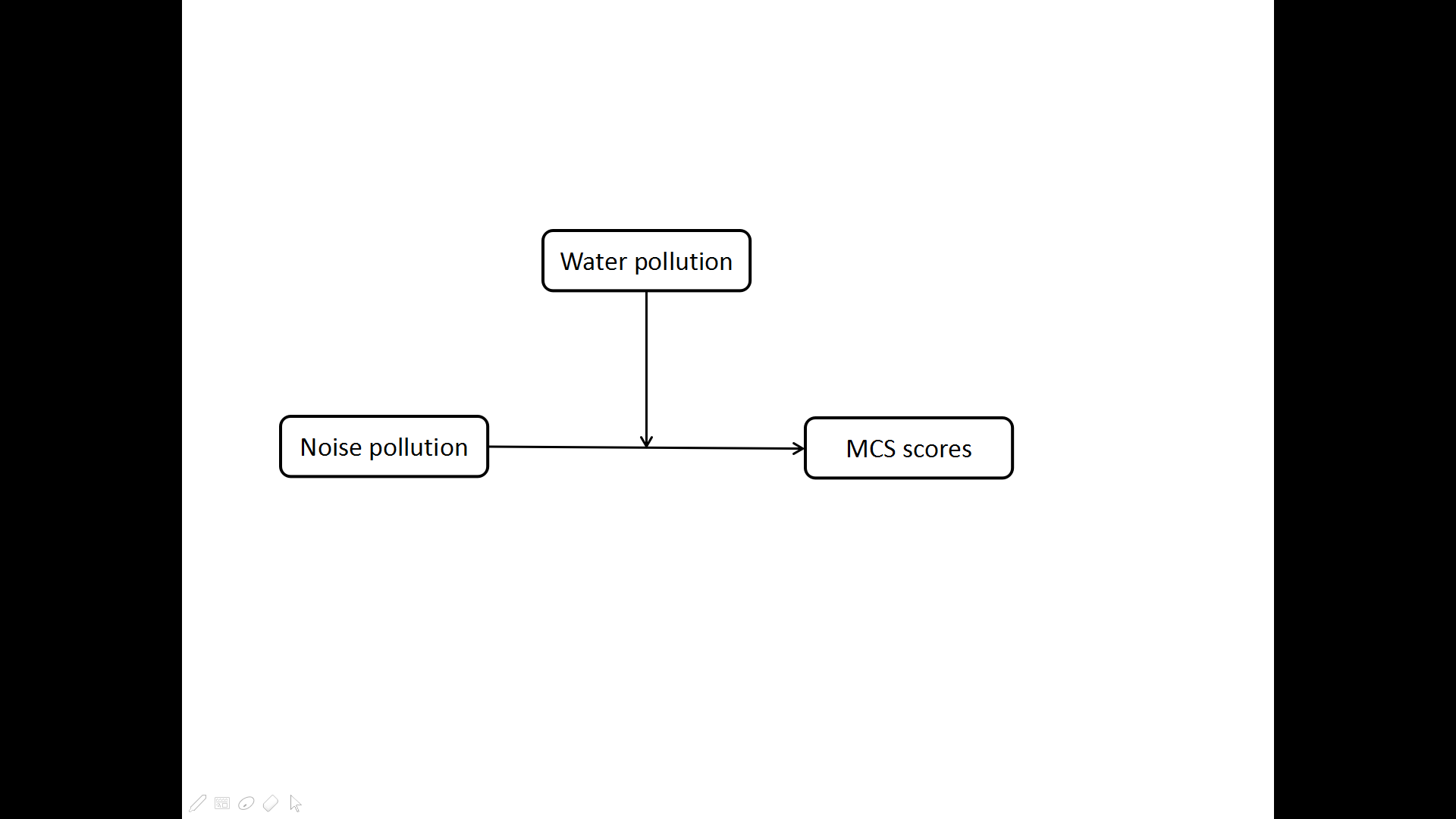


Figure 9. Potential conceptual diagram 9. Figure 10. Potential conceptual diagram 10.


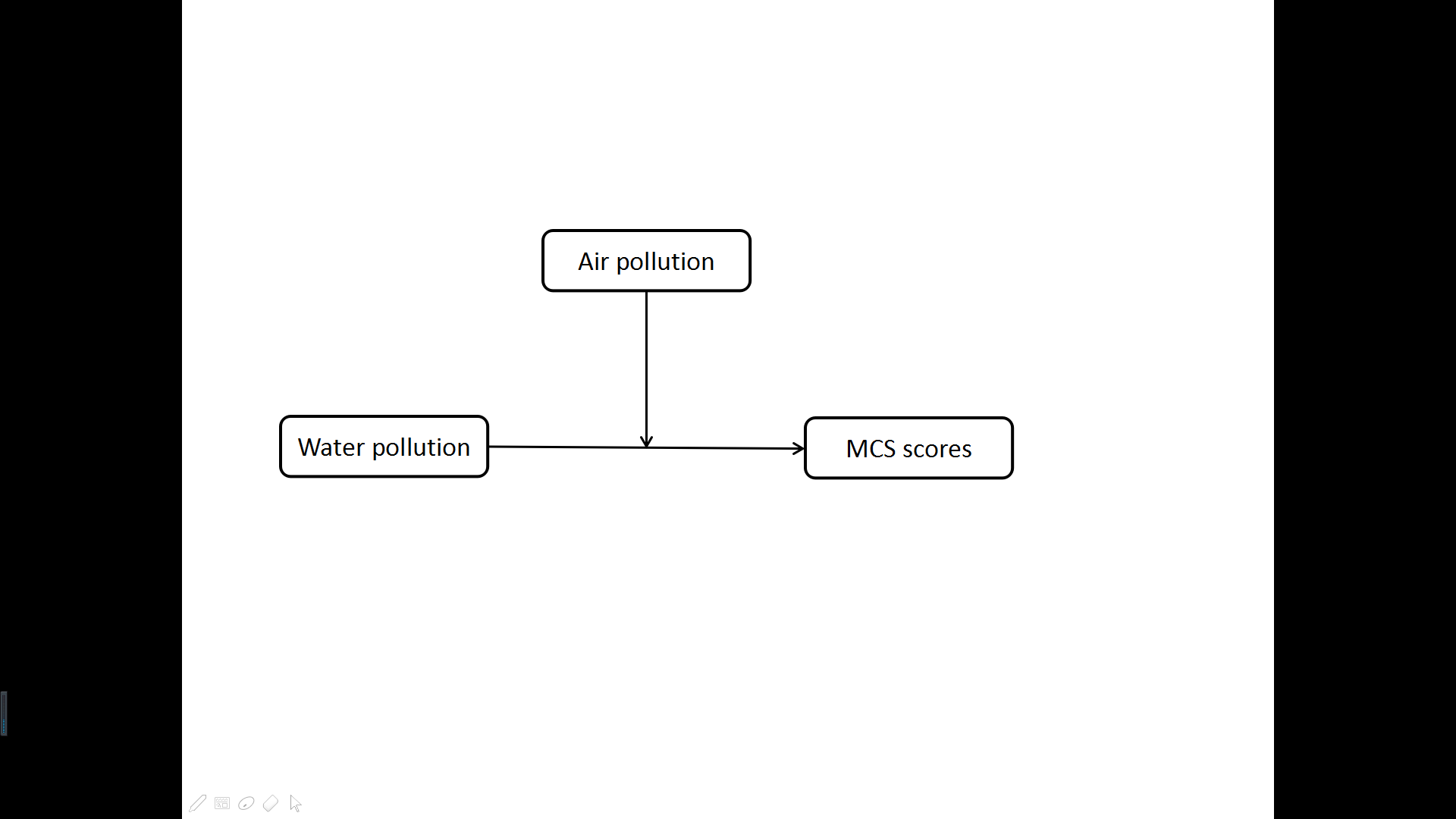

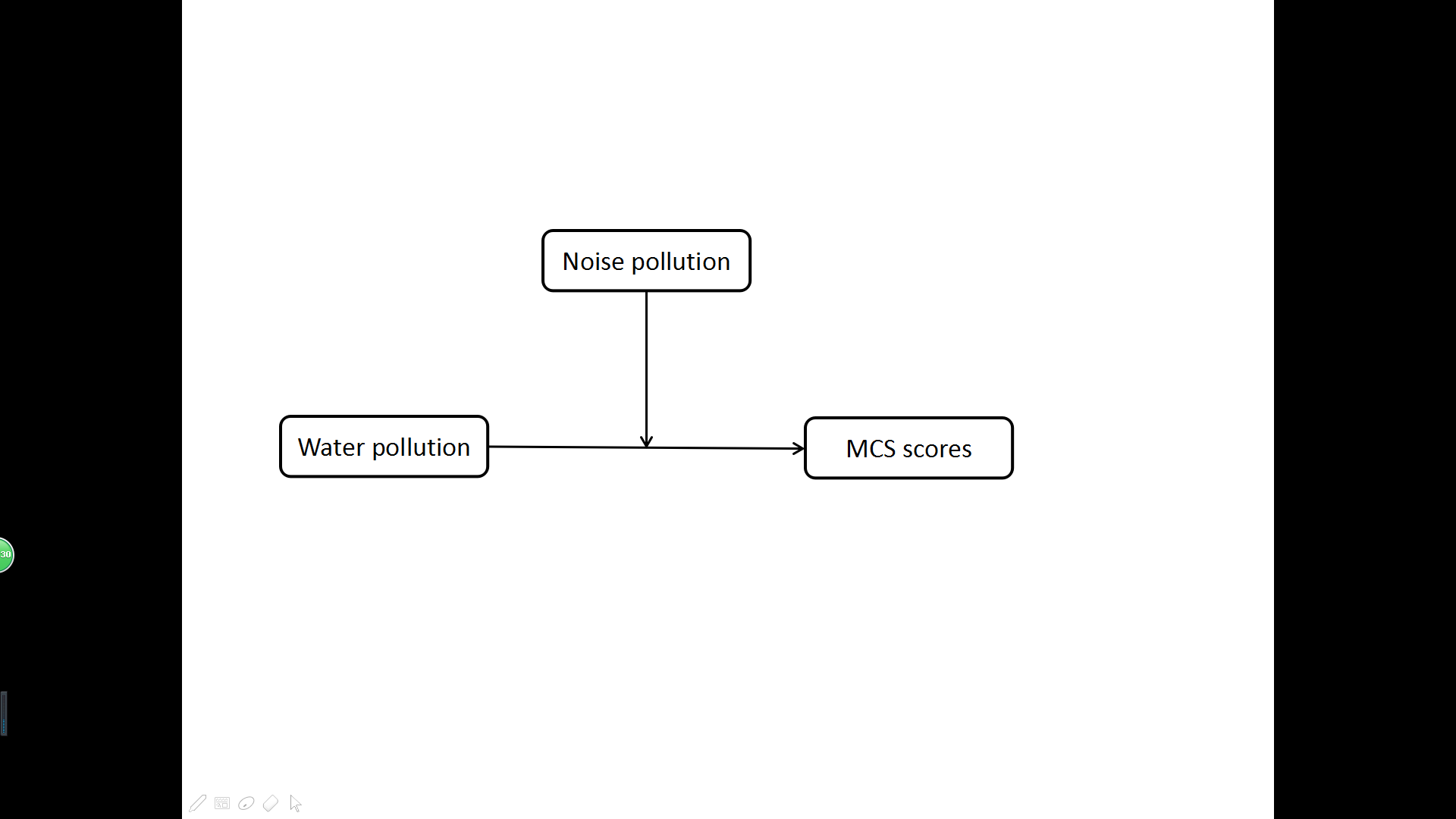


Figure 11. Potential conceptual diagram 11. Figure 12. Potential conceptual diagram 12.


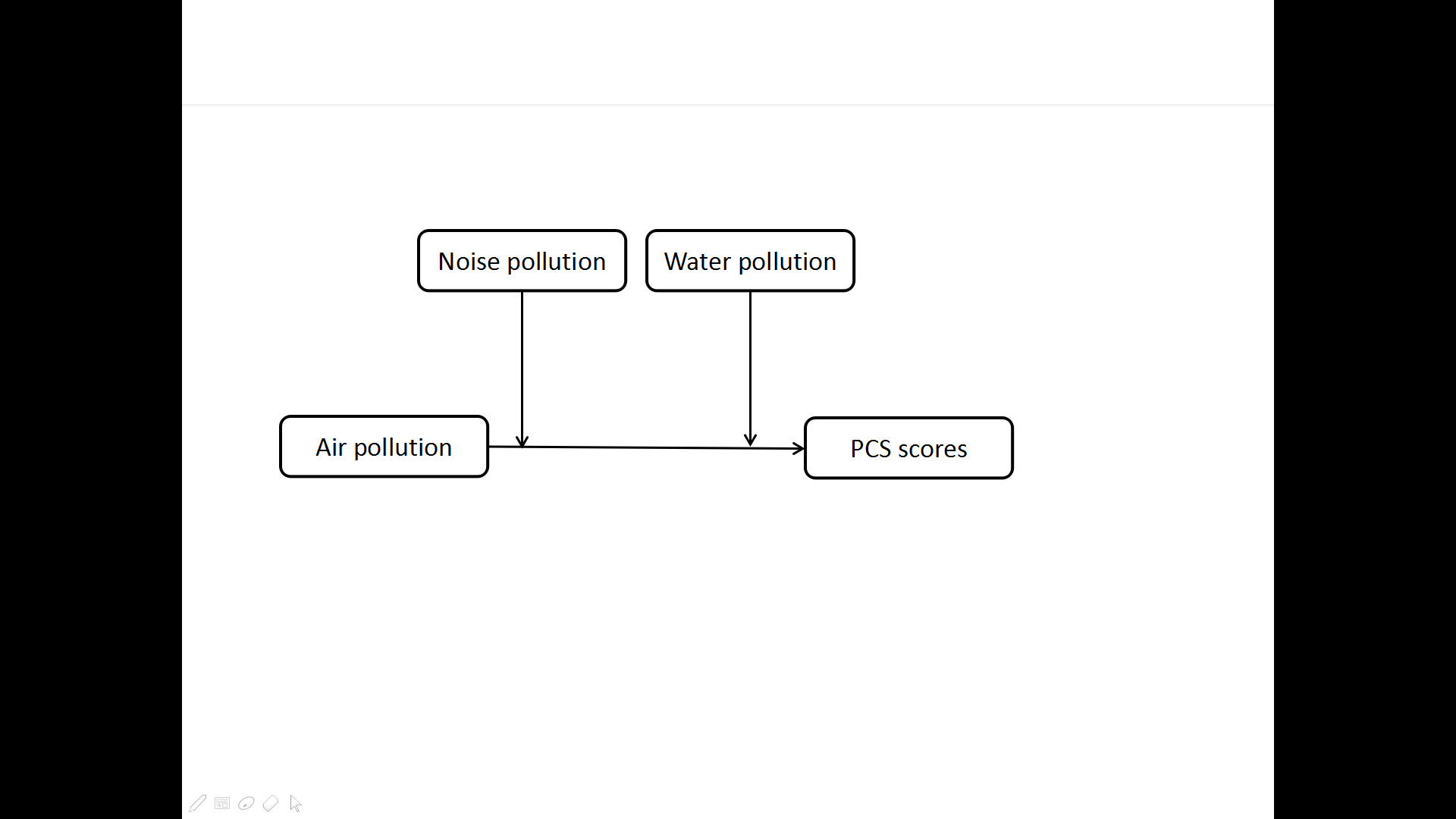

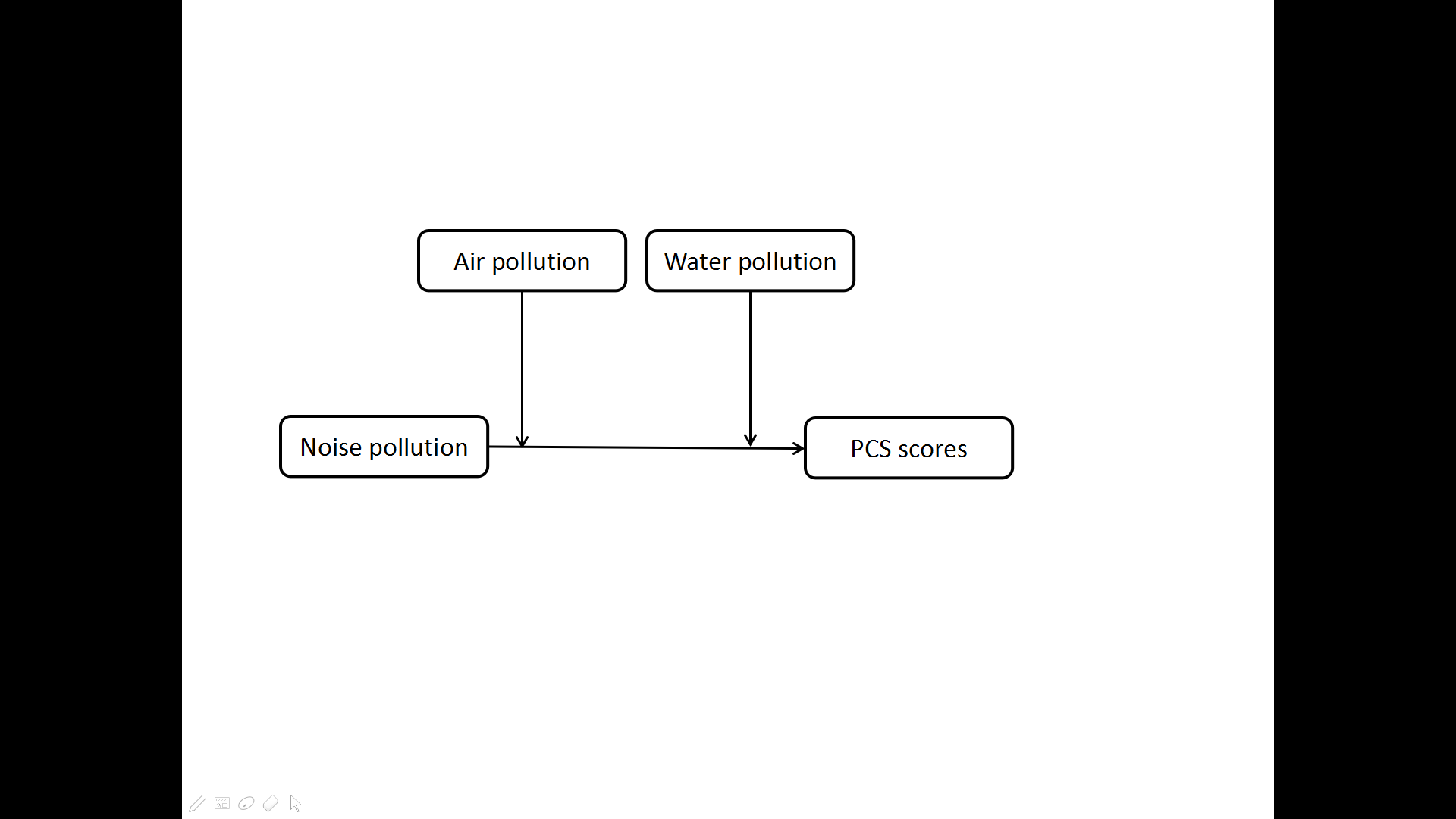


Figure 13. Potential conceptual diagram 13. Figure 14. Potential conceptual diagram 14.


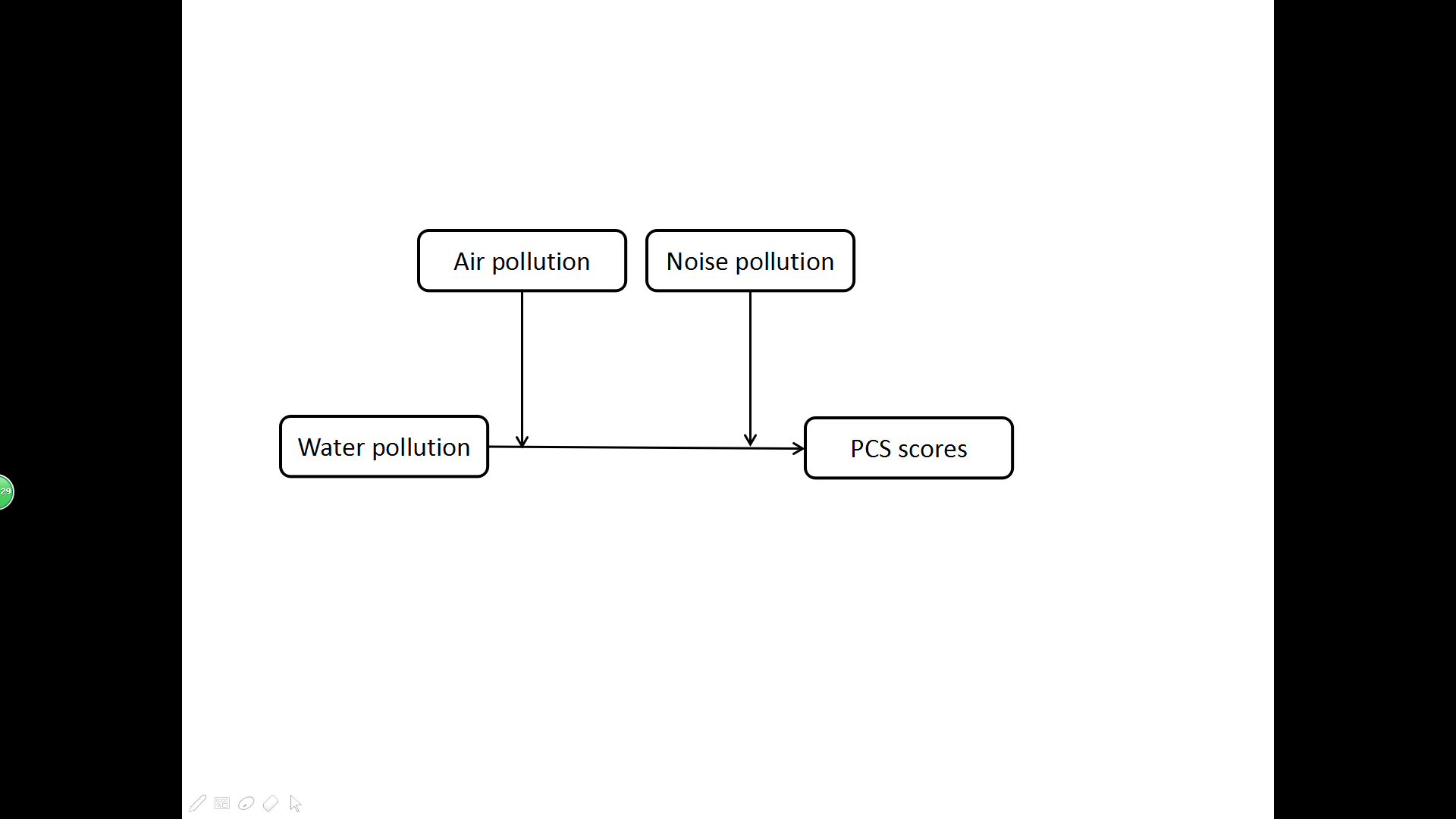

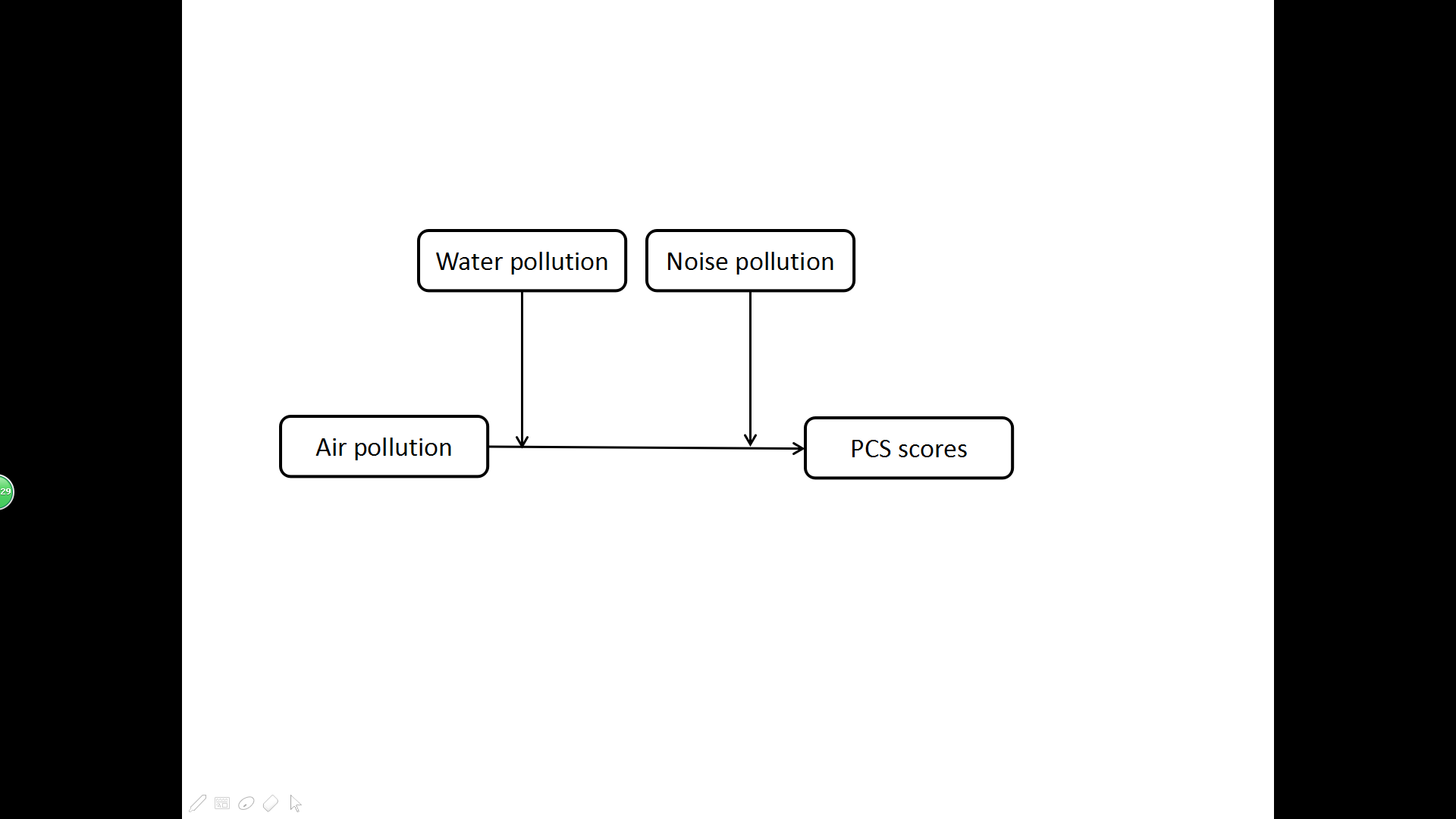


Figure 15. Potential conceptual diagram 15. Figure 16. Potential conceptual diagram 16.


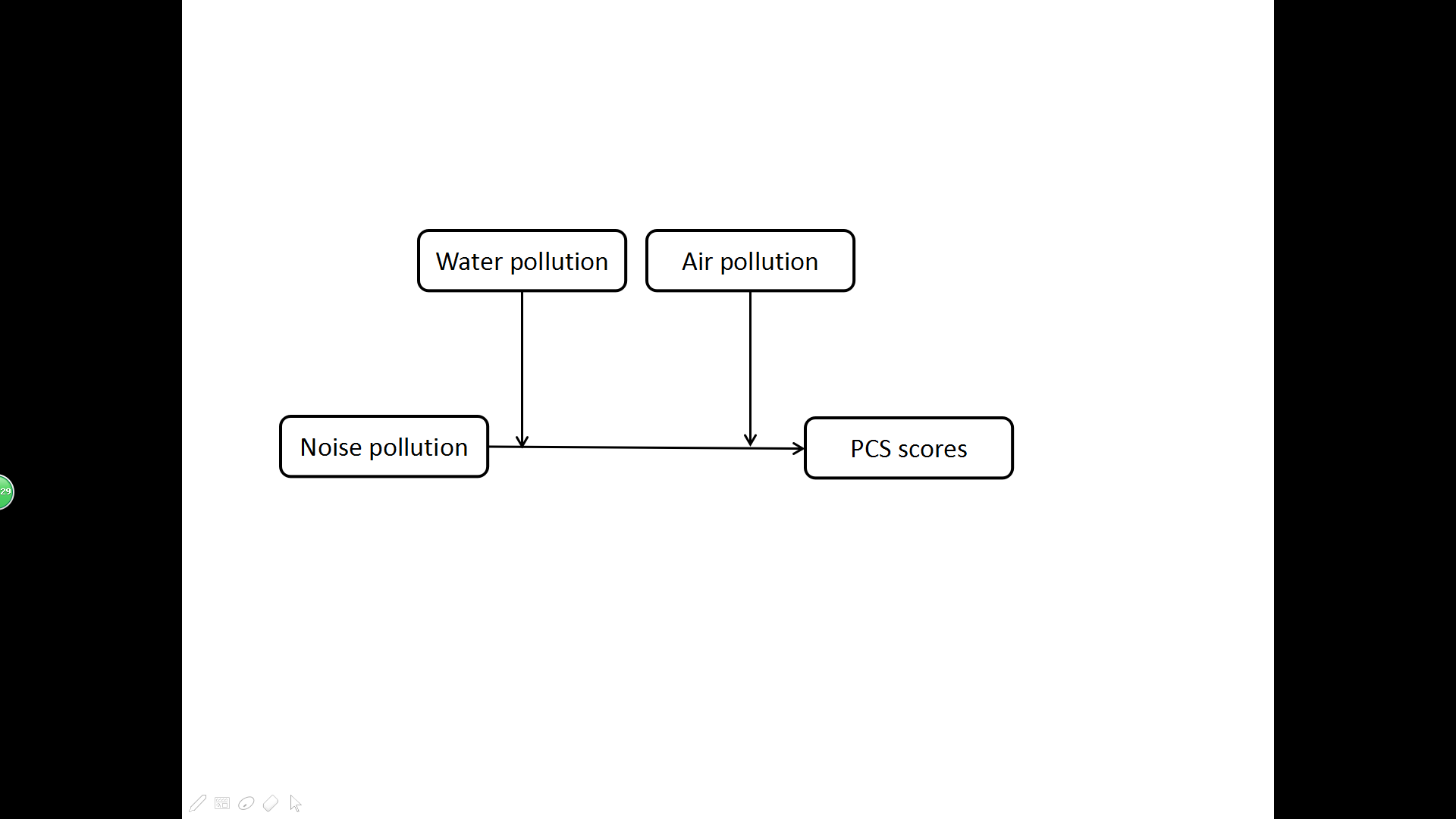

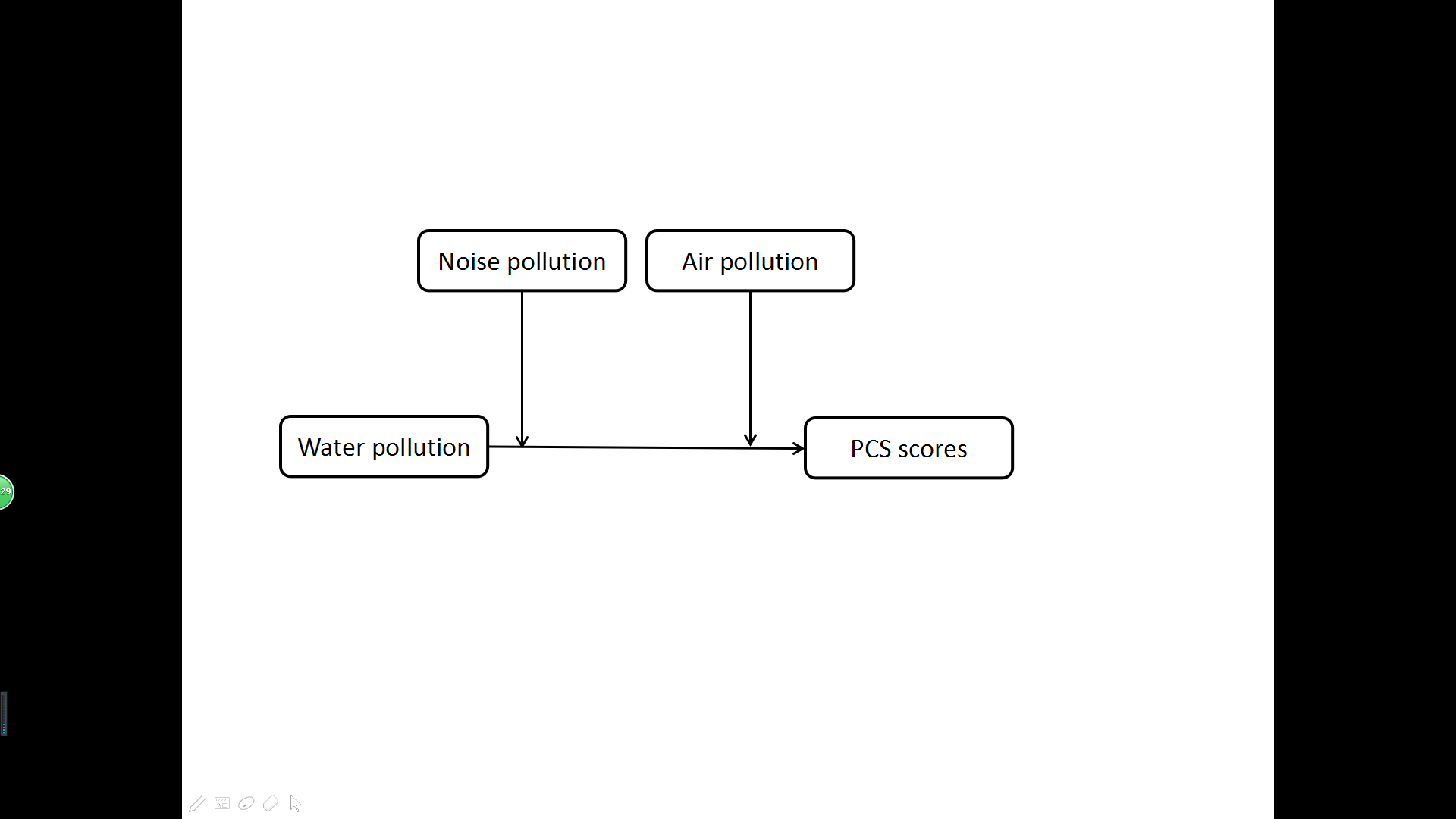


Figure 17. Potential conceptual diagram 17. Figure 18. Potential conceptual diagram 18.


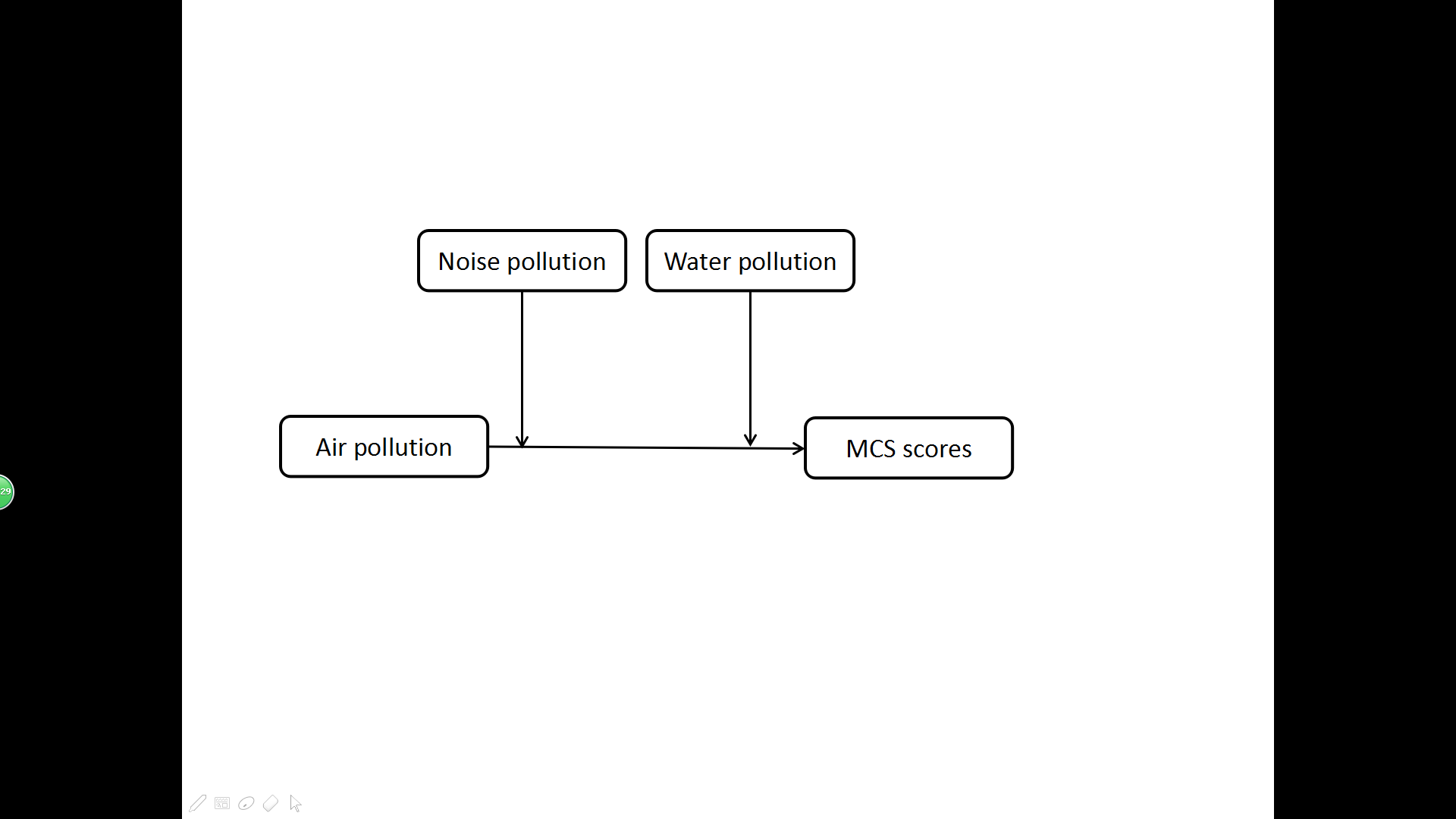

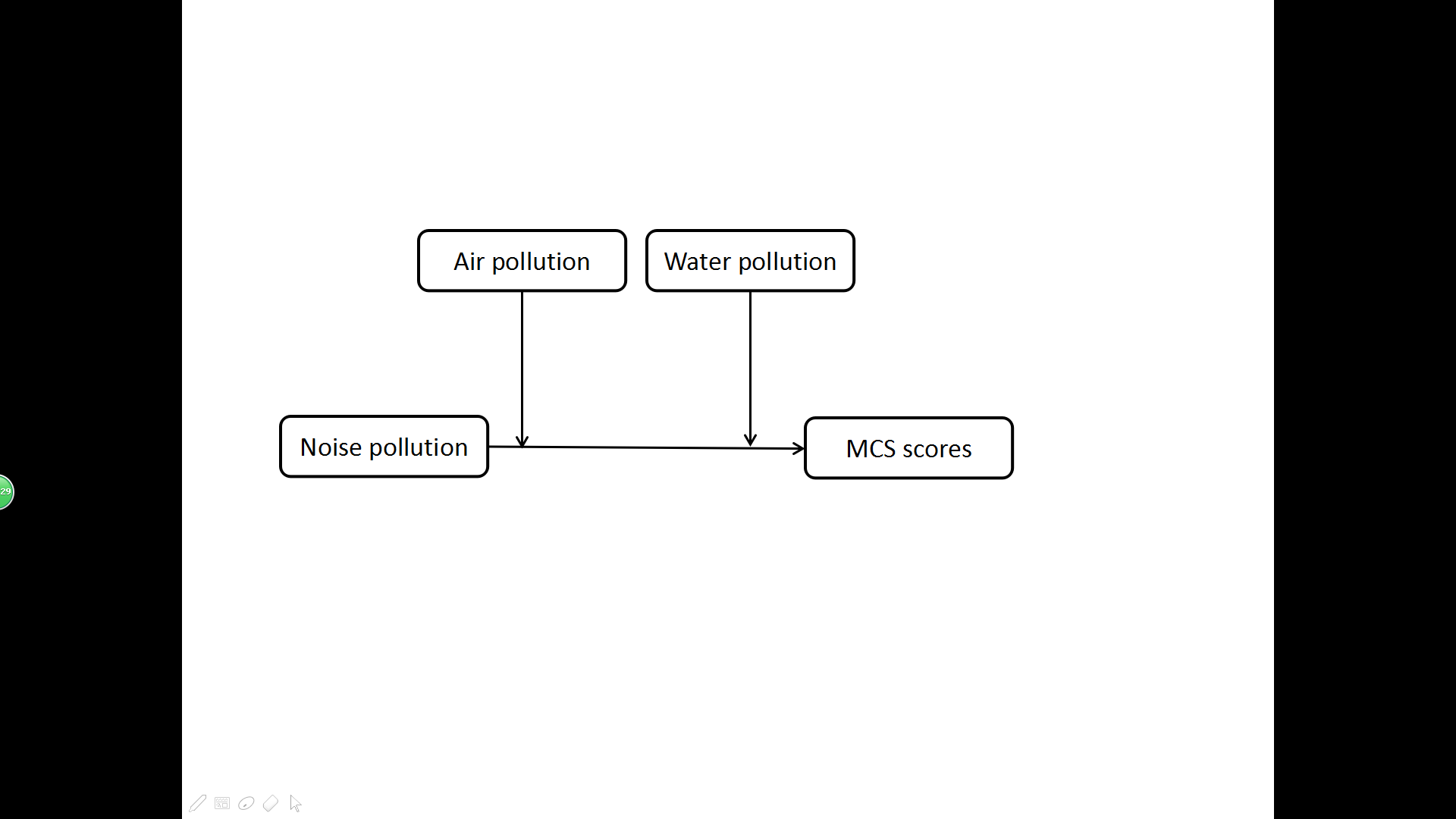


Figure 19. Potential conceptual diagram 19. Figure 20. Potential conceptual diagram 20.


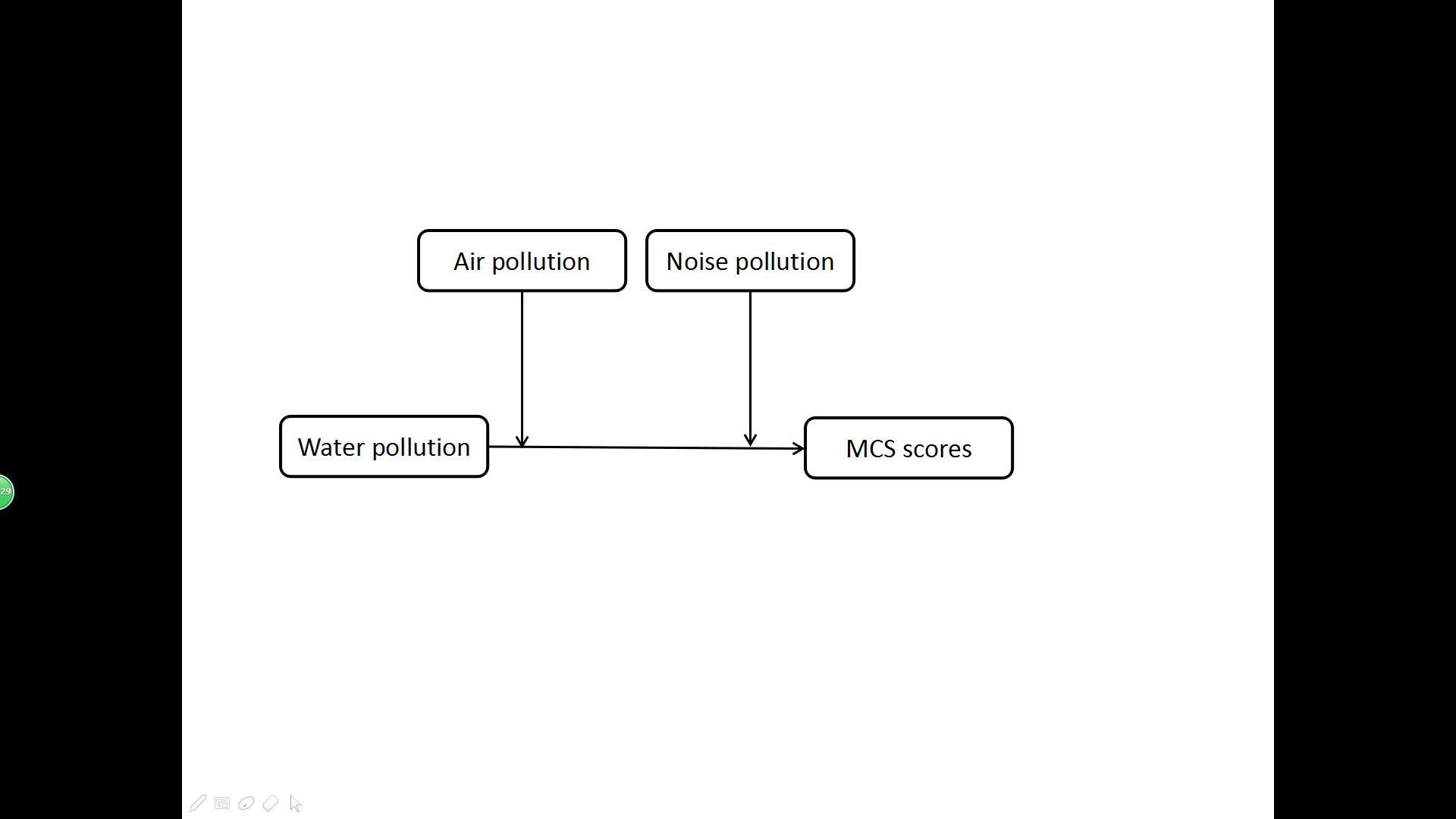

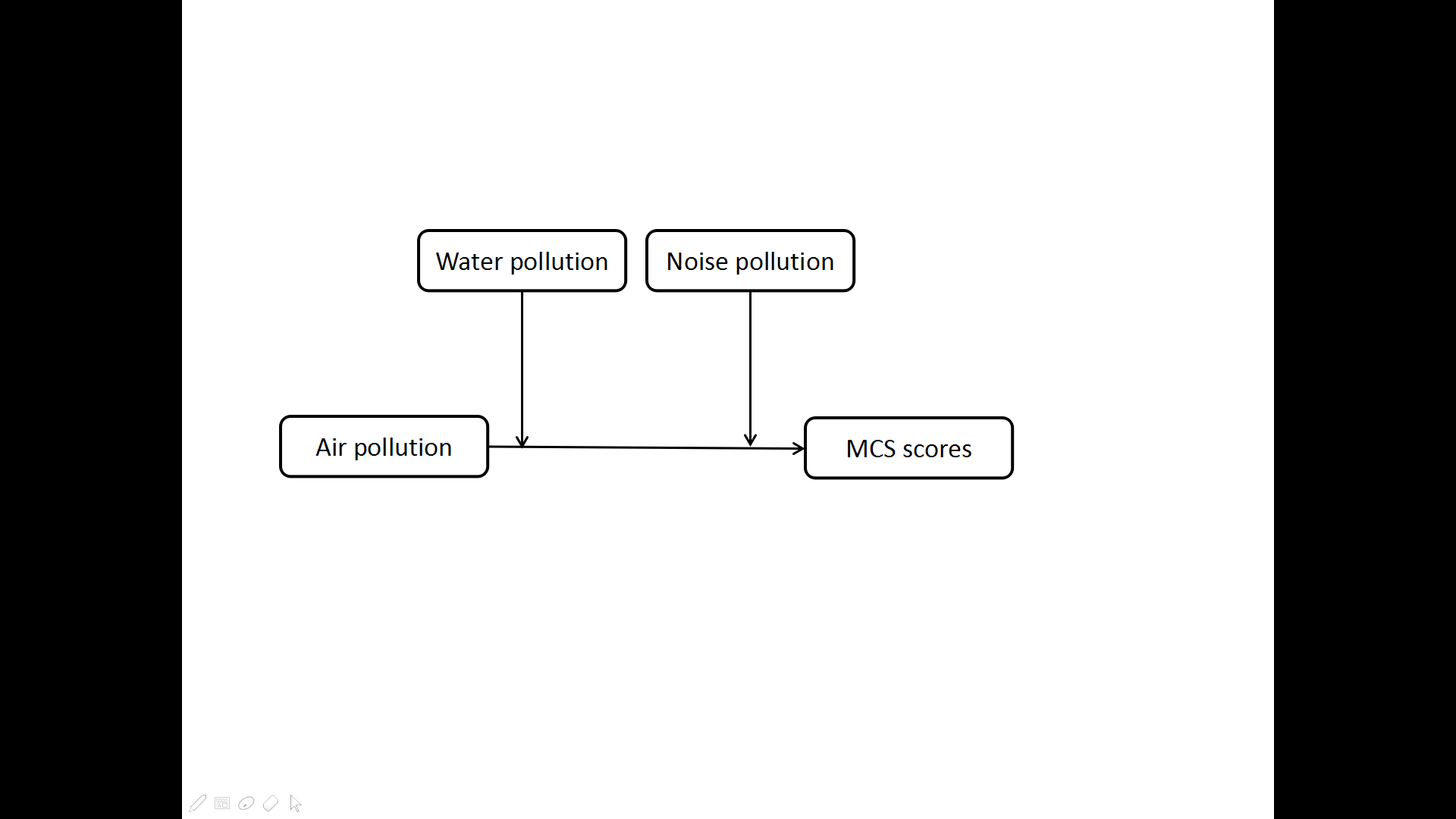


Figure 21. Potential conceptual diagram 21. Figure 22. Potential conceptual diagram 22.


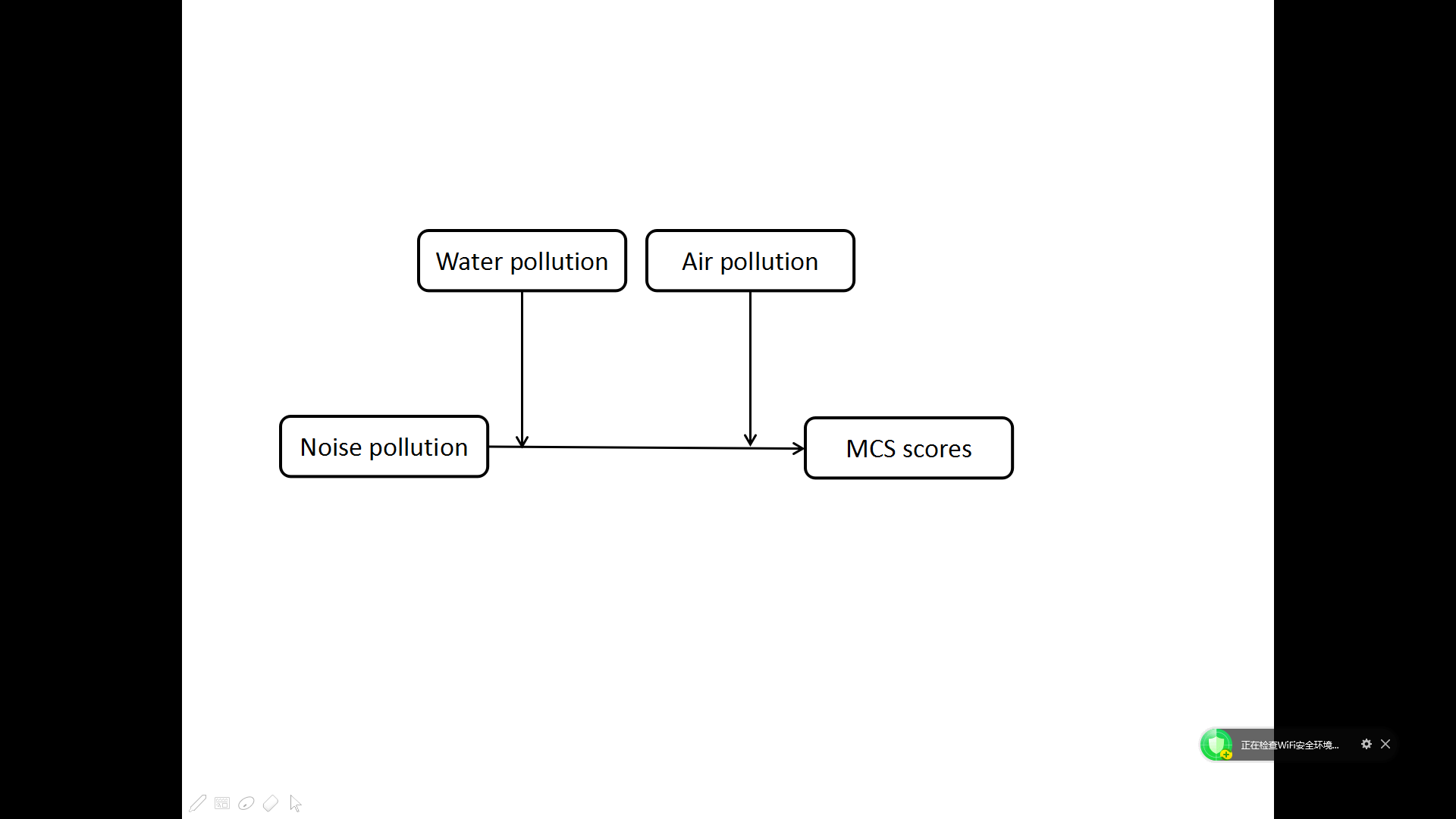

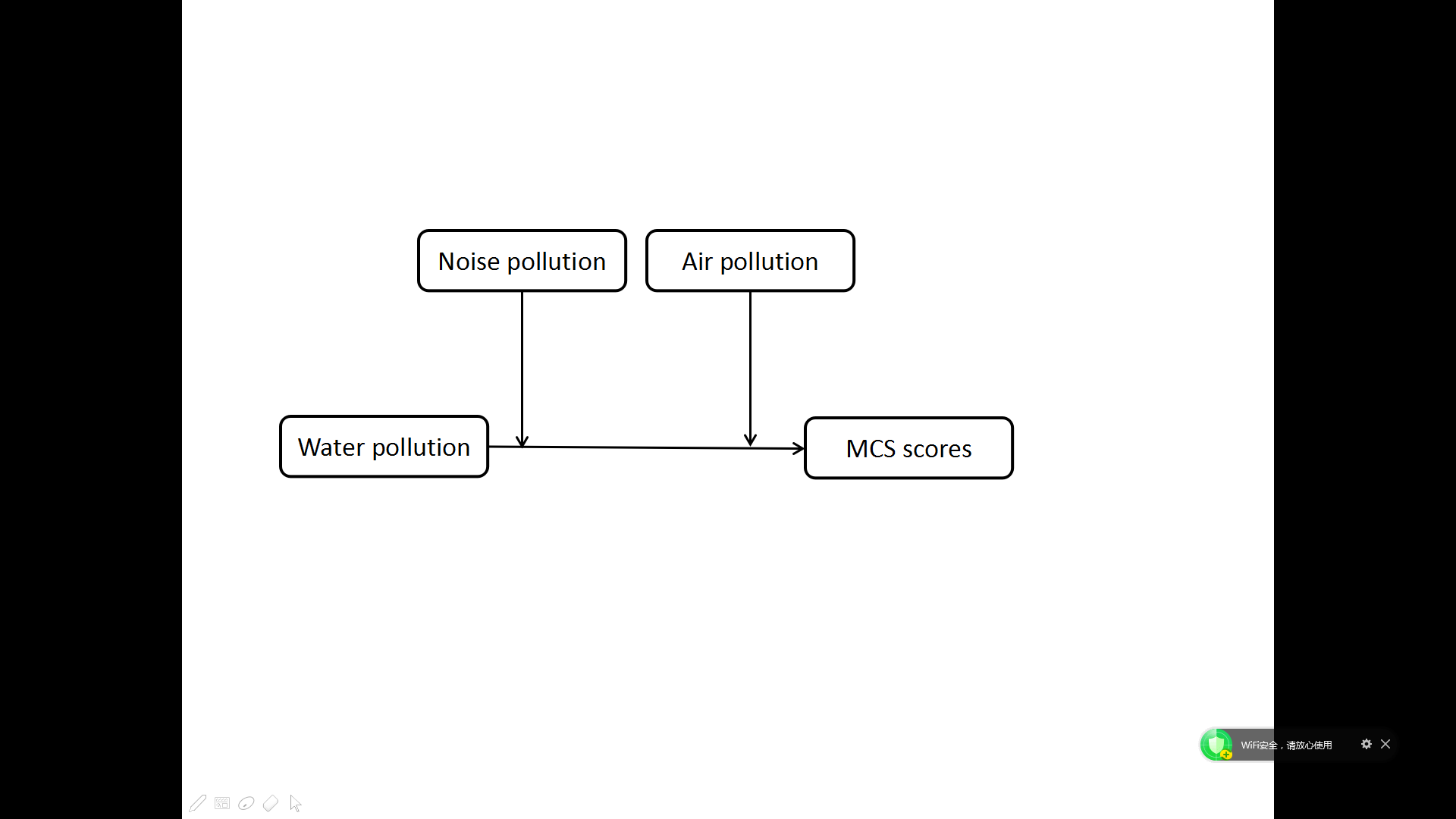


Figure 23. Potential conceptual diagram 23. Figure 24. Potential conceptual diagram 24.


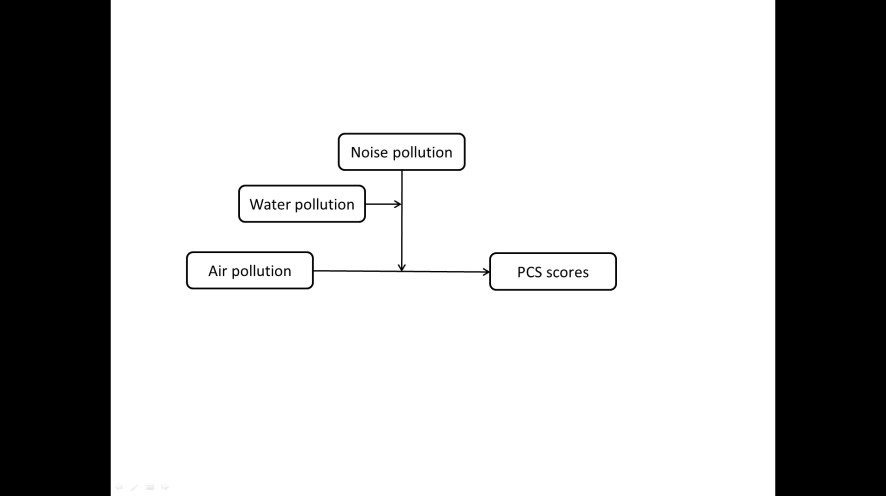

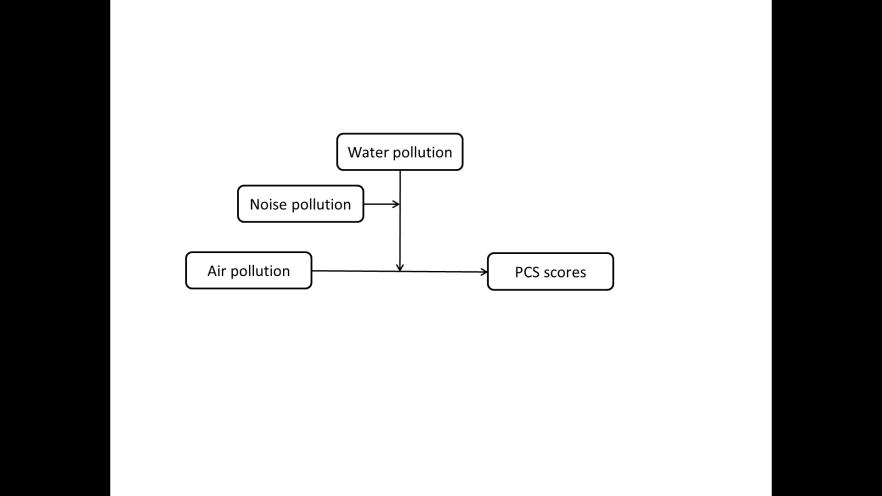


Figure 25. Potential conceptual diagram 25. Figure 26. Potential conceptual diagram 26.


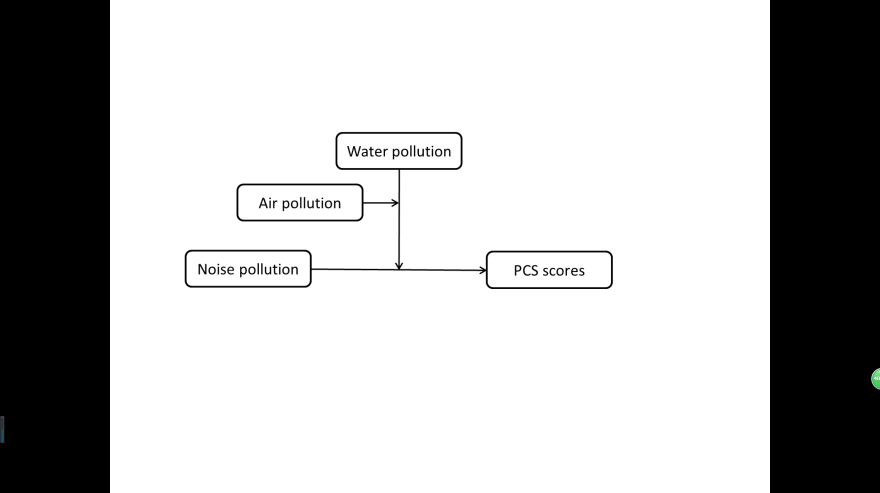

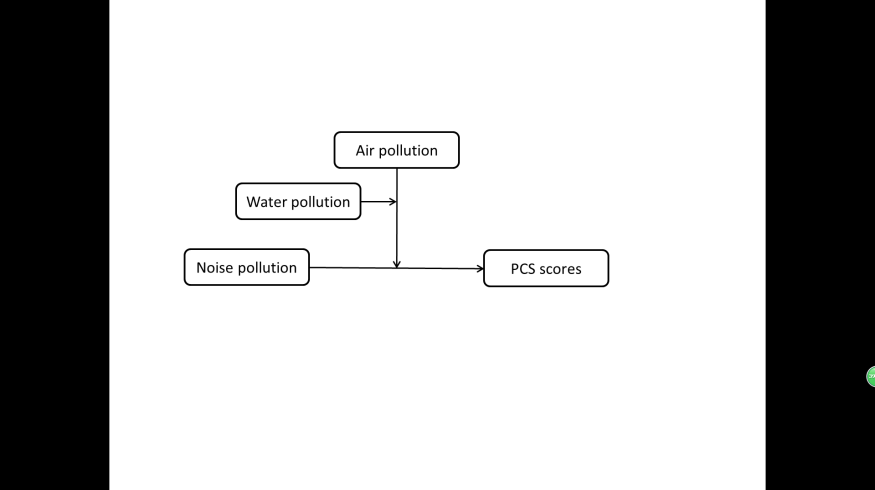


Figure 27. Potential conceptual diagram 27. Figure 28. Potential conceptual diagram 28.


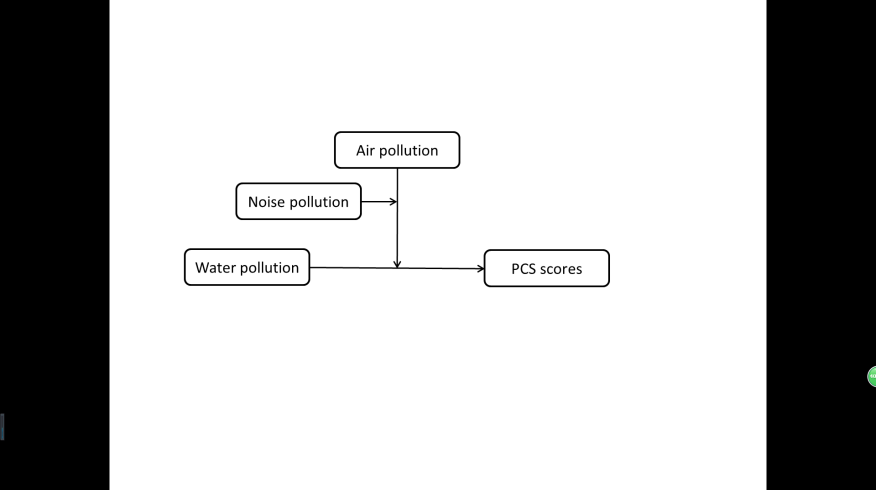

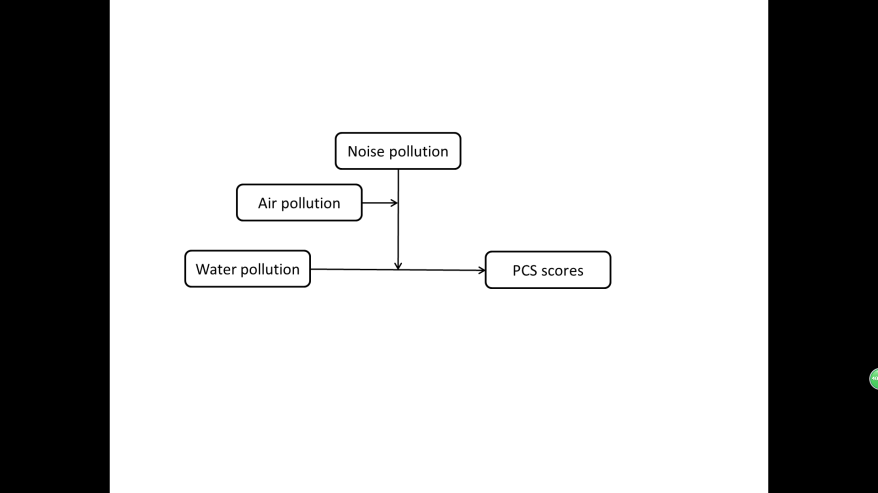


Figure 29. Potential conceptual diagram 29. Figure 30. Potential conceptual diagram 30.


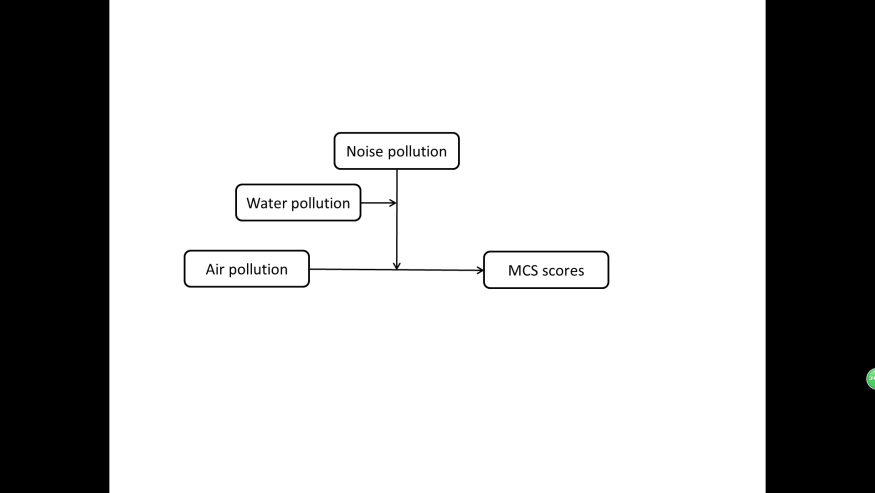

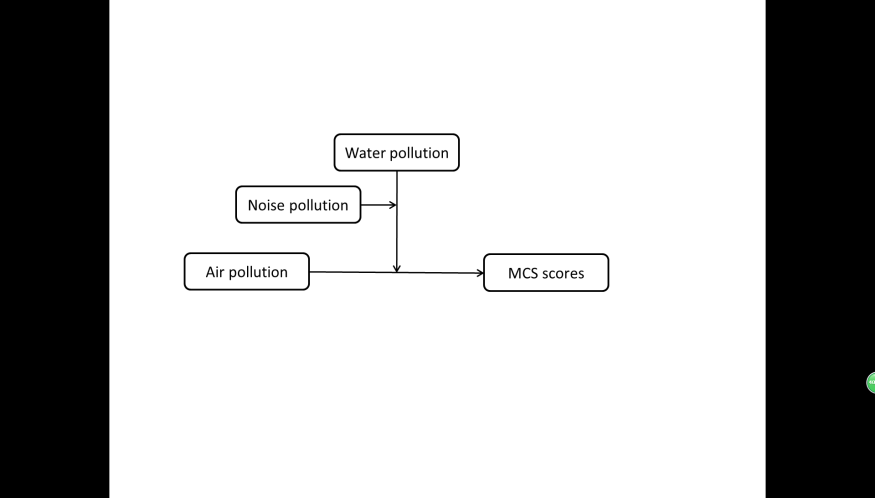


Figure 31. Potential conceptual diagram 31. Figure 32. Potential conceptual diagram 32.


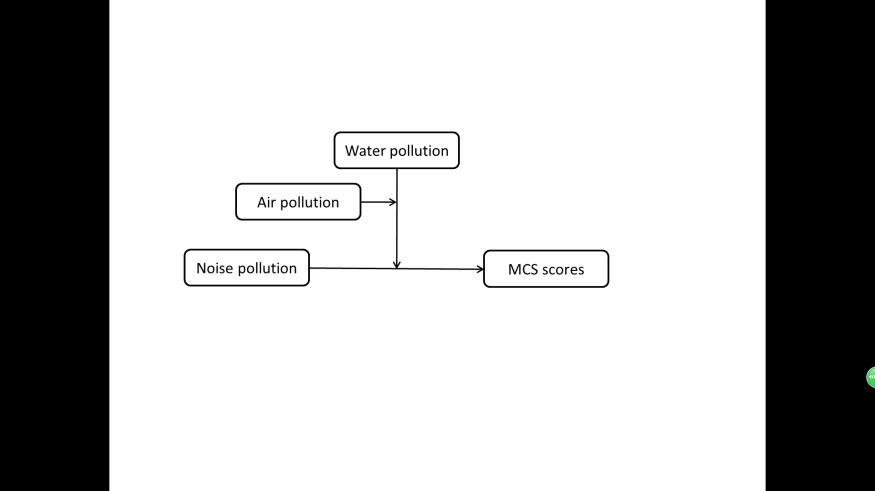

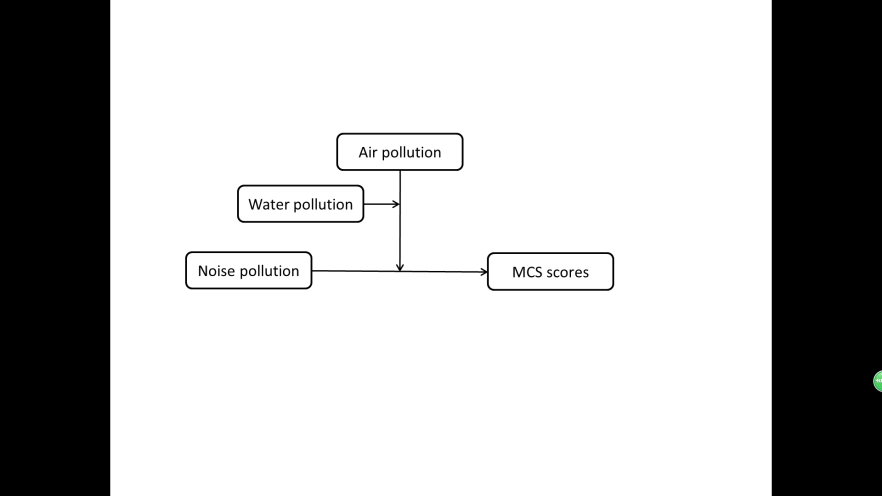


Figure 33. Potential conceptual diagram 33. Figure 34. Potential conceptual diagram 34.


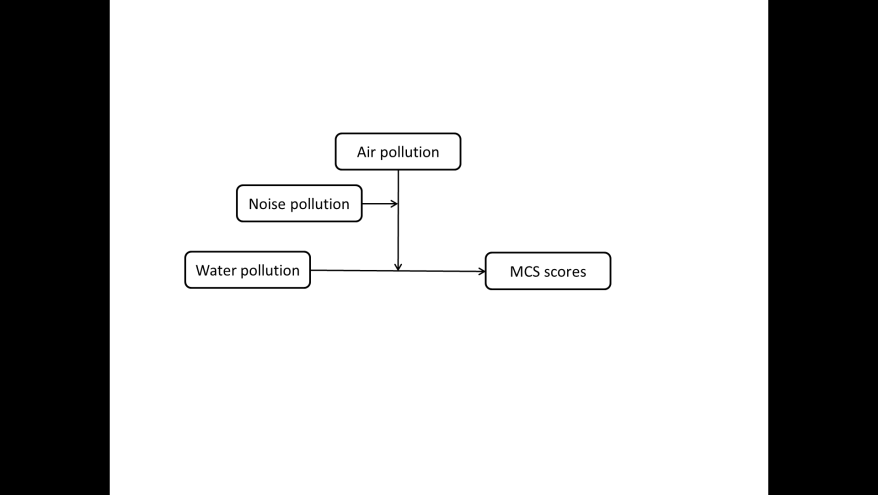

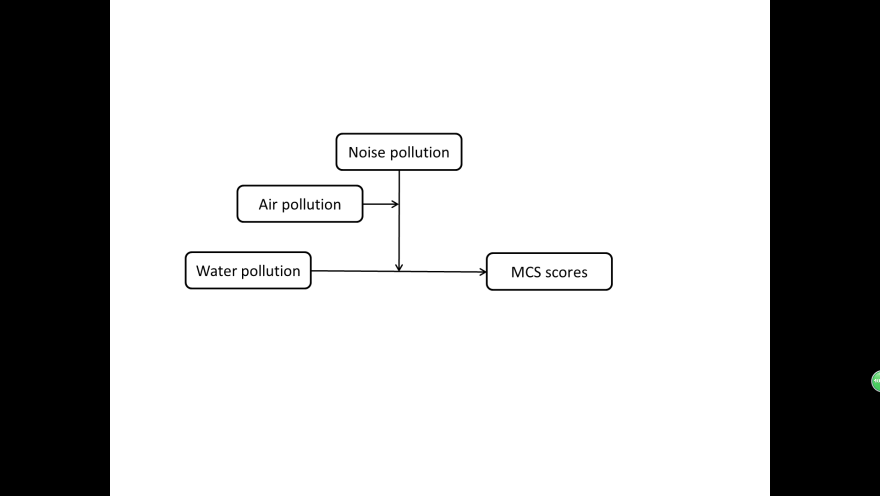


Figure 35. Potential conceptual diagram 35. Figure 36. Potential conceptual diagram 36.
