# Supplementary material for: Associations between perceived environmental pollution and health-related quality of life in a Chinese adult population
Source: Health Qual Life Outcomes. 2020 Jun 23;18:198. doi: 10.1186/s12955-020-01442-9 (PMC7310336; doi:10.1186/s12955-020-01442-9)
Supplement: Supplementary file 3 — Additional file 3 Statistical outcomes of potential conceptual diagrams. [file 12955_2020_1442_MOESM3_ESM.docx]

**Statistical outcomes of potential conceptual diagram 1.**


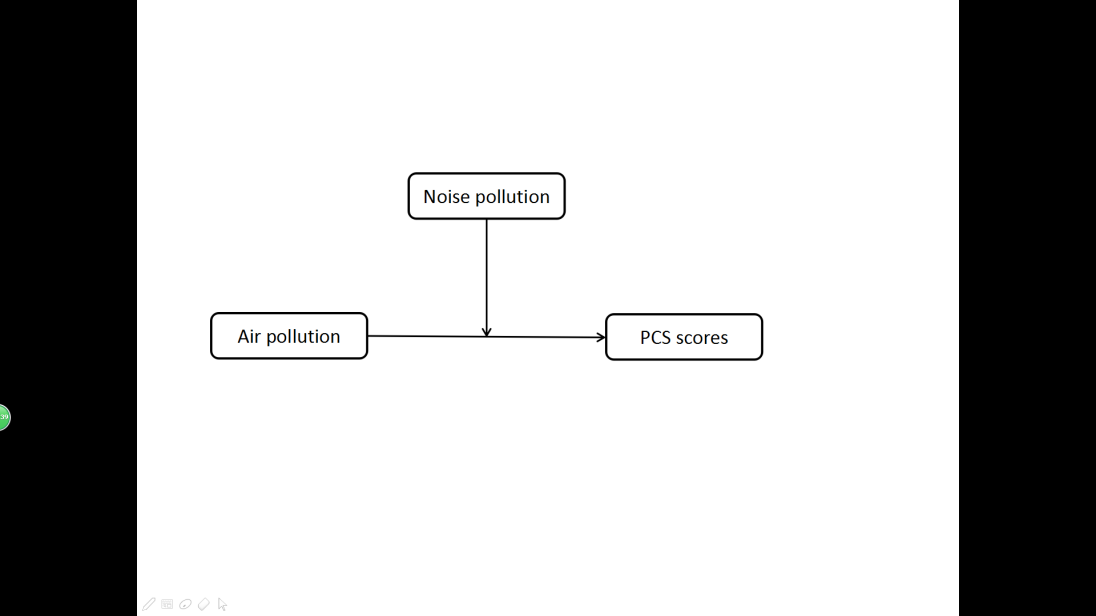


Figure 1. Conceptual diagram

Model = 1 Y = agg_phys X = air M = noise

Statistical Controls:CONTROL= employment bmi hhdnum religion alcohg smokg

Sample size 3701

**************************************************************************

Outcome: agg_phys

Model Summary

R R-sq MSE F df1 df2 p

.3141 .0987 108.2506 41.6327 9.0000 3691.0000 .0000

Model

coeff se t p LLCI ULCI

constant 43.6188 1.4097 30.9430 .0000 40.8551 46.3826

noise 1.0257 .4335 2.3658 .0180 .1757 1.8756

air .3777 .4341 .8700 .3844 -.4735 1.2289

int_1 -1.1309 .8402 -1.3460 .1784 -2.7781 .5164

employment -5.3164 .3938 -13.5012 .0000 -6.0885 -4.5444

bmi .1599 .0547 2.9245 .0035 .0527 .2672

hhdnum .7556 .1667 4.5339 .0000 .4288 1.0823

religion 1.4756 .5365 2.7505 .0060 .4238 2.5274

alcohg -.6854 .4088 -1.6766 .0937 -1.4868 .1161

smokg 3.4405 .3873 8.8831 .0000 2.6811 4.1998

Interactions:

int_1 air X noise

*************************************************************************

Conditional effect of X on Y at values of the moderator(s):

noise Effect se t p LLCI ULCI

-.3002 .7172 .5580 1.2851 .1988 -.3769 1.8113

.6998 -.4137 .6292 -.6575 .5109 -1.6473 .8199

**************************************************************************

Data for visualizing conditional effect of X on Y

Paste text below into a SPSS syntax window and execute to produce plot.

DATA LIST FREE/air noise agg_phys.

BEGIN DATA.

-.2907 -.3002 49.0581

.7093 -.3002 49.7752

-.2907 .6998 50.4125

.7093 .6998 49.9988

END DATA.

GRAPH/SCATTERPLOT=air WITH agg_phys BY noise.

* Estimates are based on setting covariates to their sample means.

******************** ANALYSIS NOTES AND WARNINGS *************************

**Statistical outcomes of potential conceptual diagram 2.**


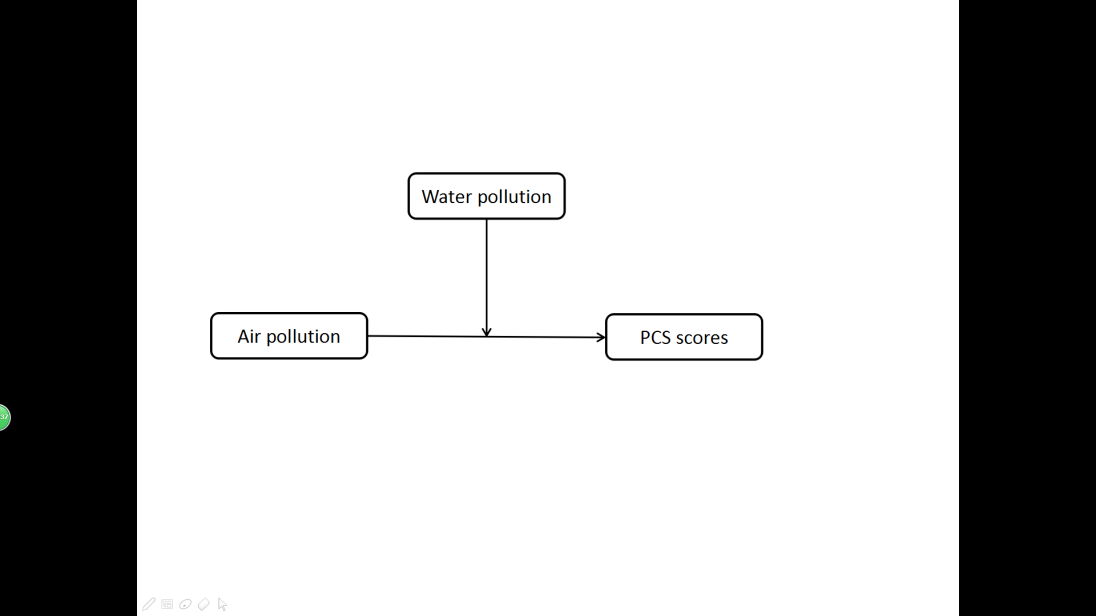


Figure 2. Conceptual diagram

Model = 1 Y = agg_phys X = air M = water

Statistical Controls:CONTROL= employment bmi hhdnum religion alcohg smokg

Sample size 3697

**************************************************************************

Outcome: agg_phys

Model Summary

R R-sq MSE F df1 df2 p

.3159 .0998 108.0639 42.1580 9.0000 3687.0000 .0000

Model

coeff se t p LLCI ULCI

constant 43.2162 1.4080 30.6932 .0000 40.4557 45.9768

water -1.1953 .5185 -2.3055 .0212 -2.2118 -.1788

air .8871 .4426 2.0044 .0451 .0194 1.7548

int_1 1.8177 .9224 1.9706 .0488 .0092 3.6261

employment -5.3334 .3941 -13.5340 .0000 -6.1060 -4.5607

bmi .1668 .0545 3.0626 .0022 .0600 .2735

hhdnum .7532 .1662 4.5322 .0000 .4274 1.0790

religion 1.4208 .5371 2.6453 .0082 .3678 2.4739

alcohg -.6960 .4091 -1.7012 .0890 -1.4981 .1061

smokg 3.4773 .3869 8.9869 .0000 2.7186 4.2359

Interactions:

int_1 air X water

*************************************************************************

Conditional effect of X on Y at values of the moderator(s):

water Effect se t p LLCI ULCI

-.2659 .4038 .5354 .7541 .4508 -.6460 1.4535

.7341 2.2214 .7548 2.9432 .0033 .7416 3.7012

**************************************************************************

Data for visualizing conditional effect of X on Y

Paste text below into a SPSS syntax window and execute to produce plot.

DATA LIST FREE/air water agg_phys.

BEGIN DATA.

-.2905 -.2659 49.4782

.7095 -.2659 49.8820

-.2905 .7341 47.7548

.7095 .7341 49.9763

END DATA.

GRAPH/SCATTERPLOT=air WITH agg_phys BY water.

* Estimates are based on setting covariates to their sample means.

******************** ANALYSIS NOTES AND WARNINGS *************************

**Statistical outcomes of potential conceptual diagram 3.**


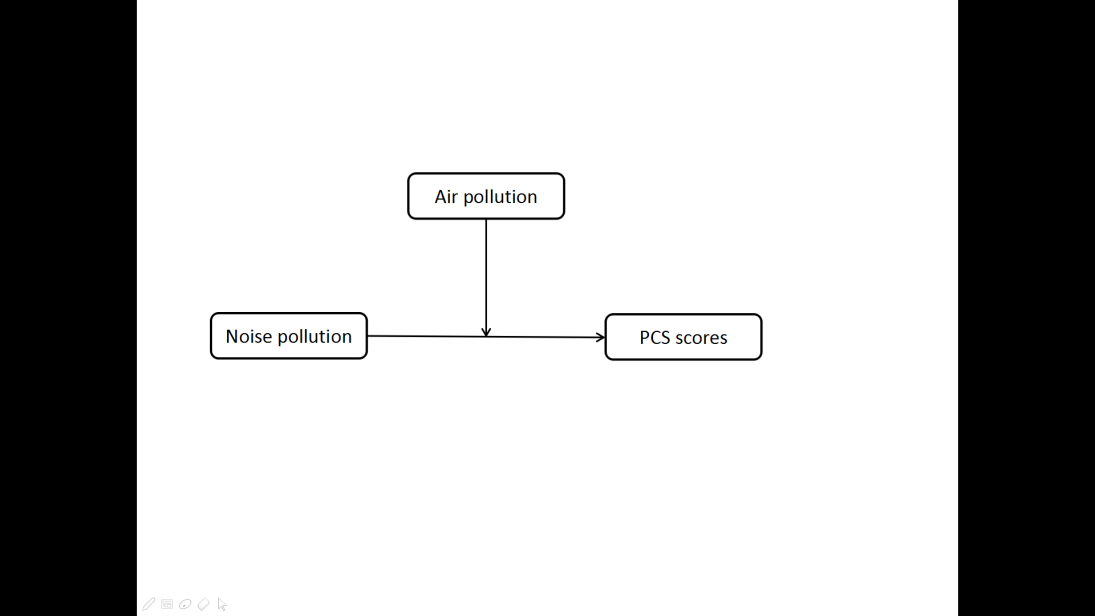


Figure 3. Conceptual diagram

Model = 1 Y = agg_phys X = noise M = air

Statistical Controls:CONTROL= employment bmi hhdnum religion alcohg smokg

Sample size 3701

**************************************************************************

Outcome: agg_phys

Model Summary

R R-sq MSE F df1 df2 p

.3141 .0987 108.2506 41.6327 9.0000 3691.0000 .0000

Model

coeff se t p LLCI ULCI

constant 43.6188 1.4097 30.9430 .0000 40.8551 46.3826

air .3777 .4341 .8700 .3844 -.4735 1.2289

noise 1.0257 .4335 2.3658 .0180 .1757 1.8756

int_1 -1.1309 .8402 -1.3460 .1784 -2.7781 .5164

employment -5.3164 .3938 -13.5012 .0000 -6.0885 -4.5444

bmi .1599 .0547 2.9245 .0035 .0527 .2672

hhdnum .7556 .1667 4.5339 .0000 .4288 1.0823

religion 1.4756 .5365 2.7505 .0060 .4238 2.5274

alcohg -.6854 .4088 -1.6766 .0937 -1.4868 .1161

smokg 3.4405 .3873 8.8831 .0000 2.6811 4.1998

Interactions:

int_1 noise X air

*************************************************************************

Conditional effect of X on Y at values of the moderator(s):

air Effect se t p LLCI ULCI

-.2907 1.3544 .5529 2.4498 .0143 .2705 2.4384

.7093 .2236 .6336 .3529 .7242 -1.0186 1.4658

**************************************************************************

Data for visualizing conditional effect of X on Y

Paste text below into a SPSS syntax window and execute to produce plot.

DATA LIST FREE/noise air agg_phys.

BEGIN DATA.

-.3002 -.2907 49.0581

.6998 -.2907 50.4125

-.3002 .7093 49.7752

.6998 .7093 49.9988

END DATA.

GRAPH/SCATTERPLOT=noise WITH agg_phys BY air.

* Estimates are based on setting covariates to their sample means.

******************** ANALYSIS NOTES AND WARNINGS *************************

**Statistical outcomes of potential conceptual diagram 4.**


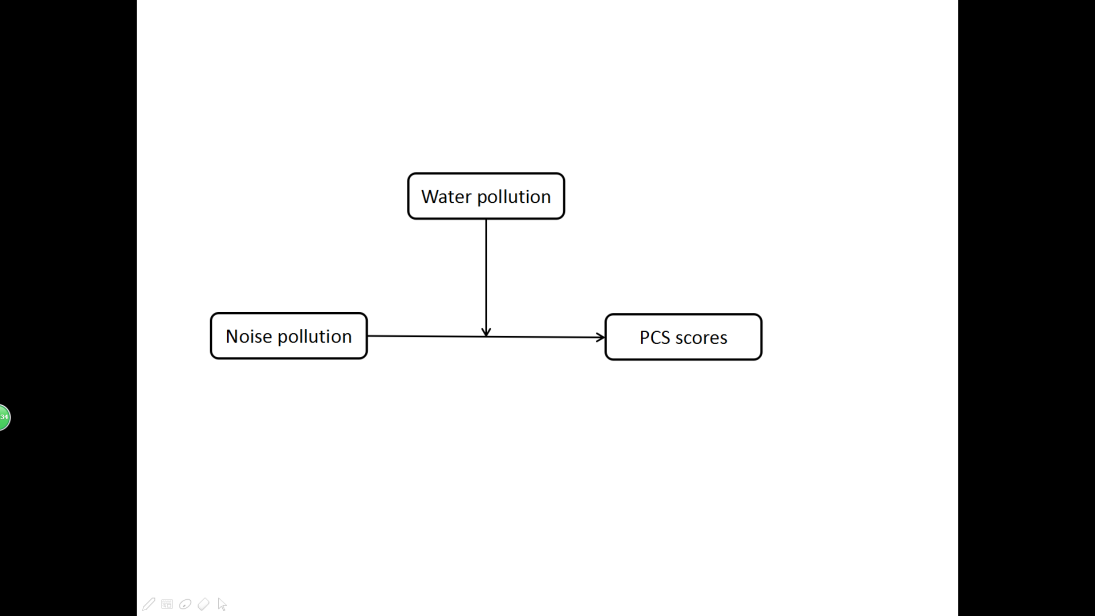


Figure 4. Conceptual diagram

Model = 1 Y = agg_phys X = noise M = water

Statistical Controls:CONTROL= employment bmi hhdnum religion alcohg smokg

Sample size 3697

**************************************************************************

Outcome: agg_phys

Model Summary

R R-sq MSE F df1 df2 p

.3159 .0998 108.0623 42.2529 9.0000 3687.0000 .0000

Model

coeff se t p LLCI ULCI

constant 43.4136 1.4024 30.9563 .0000 40.6640 46.1632

water -.7935 .4373 -1.8146 .0697 -1.6509 .0639

noise 1.2122 .3935 3.0803 .0021 .4406 1.9838

int_1 .5199 .8289 .6272 .5306 -1.1052 2.1450

employment -5.3598 .3941 -13.6004 .0000 -6.1325 -4.5872

bmi .1652 .0544 3.0339 .0024 .0584 .2719

hhdnum .7552 .1649 4.5790 .0000 .4318 1.0785

religion 1.4330 .5357 2.6749 .0075 .3827 2.4834

alcohg -.6985 .4094 -1.7063 .0880 -1.5011 .1041

smokg 3.4352 .3879 8.8551 .0000 2.6746 4.1958

Interactions:

int_1 noise X water

*************************************************************************

Conditional effect of X on Y at values of the moderator(s):

water Effect se t p LLCI ULCI

-.2659 1.0740 .4760 2.2565 .0241 .1408 2.0072

.7341 1.5939 .6793 2.3464 .0190 .2621 2.9257

**************************************************************************

Data for visualizing conditional effect of X on Y

Paste text below into a SPSS syntax window and execute to produce plot.

DATA LIST FREE/noise water agg_phys.

BEGIN DATA.

-.3000 -.2659 49.3177

.7000 -.2659 50.3916

-.3000 .7341 48.3682

.7000 .7341 49.9621

END DATA.

GRAPH/SCATTERPLOT=noise WITH agg_phys BY water.

* Estimates are based on setting covariates to their sample means.

******************** ANALYSIS NOTES AND WARNINGS *************************

**Statistical outcomes of potential conceptual diagram 5.**


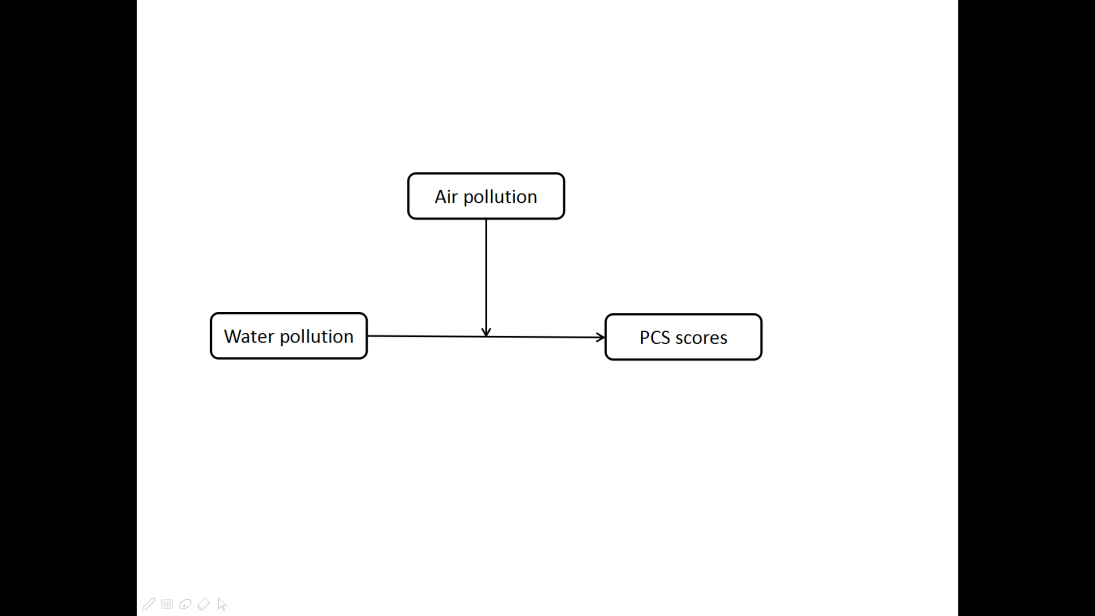


Figure 5. Conceptual diagram

Model = 1 Y = agg_phys X = water M = air

Statistical Controls:CONTROL= employment bmi hhdnum religion alcohg smokg

Sample size 3697

**************************************************************************

Outcome: agg_phys

Model Summary

R R-sq MSE F df1 df2 p

.3159 .0998 108.0639 42.1580 9.0000 3687.0000 .0000

Model

coeff se t p LLCI ULCI

constant 43.2162 1.4080 30.6932 .0000 40.4557 45.9768

air .8871 .4426 2.0044 .0451 .0194 1.7548

water -1.1953 .5185 -2.3055 .0212 -2.2118 -.1788

int_1 1.8177 .9224 1.9706 .0488 .0092 3.6261

employment -5.3334 .3941 -13.5340 .0000 -6.1060 -4.5607

bmi .1668 .0545 3.0626 .0022 .0600 .2735

hhdnum .7532 .1662 4.5322 .0000 .4274 1.0790

religion 1.4208 .5371 2.6453 .0082 .3678 2.4739

alcohg -.6960 .4091 -1.7012 .0890 -1.4981 .1061

smokg 3.4773 .3869 8.9869 .0000 2.7186 4.2359

Interactions:

int_1 water X air

*************************************************************************

Conditional effect of X on Y at values of the moderator(s):

air Effect se t p LLCI ULCI

-.2905 -1.7234 .6842 -2.5190 .0118 -3.0647 -.3820

.7095 .0943 .6211 .1518 .8794 -1.1235 1.3121

**************************************************************************

Data for visualizing conditional effect of X on Y

Paste text below into a SPSS syntax window and execute to produce plot.

DATA LIST FREE/water air agg_phys.

BEGIN DATA.

-.2659 -.2905 49.4782

.7341 -.2905 47.7548

-.2659 .7095 49.8820

.7341 .7095 49.9763

END DATA.

GRAPH/SCATTERPLOT=water WITH agg_phys BY air.

* Estimates are based on setting covariates to their sample means.

******************** ANALYSIS NOTES AND WARNINGS *************************

**Statistical outcomes of potential conceptual diagram 6.**


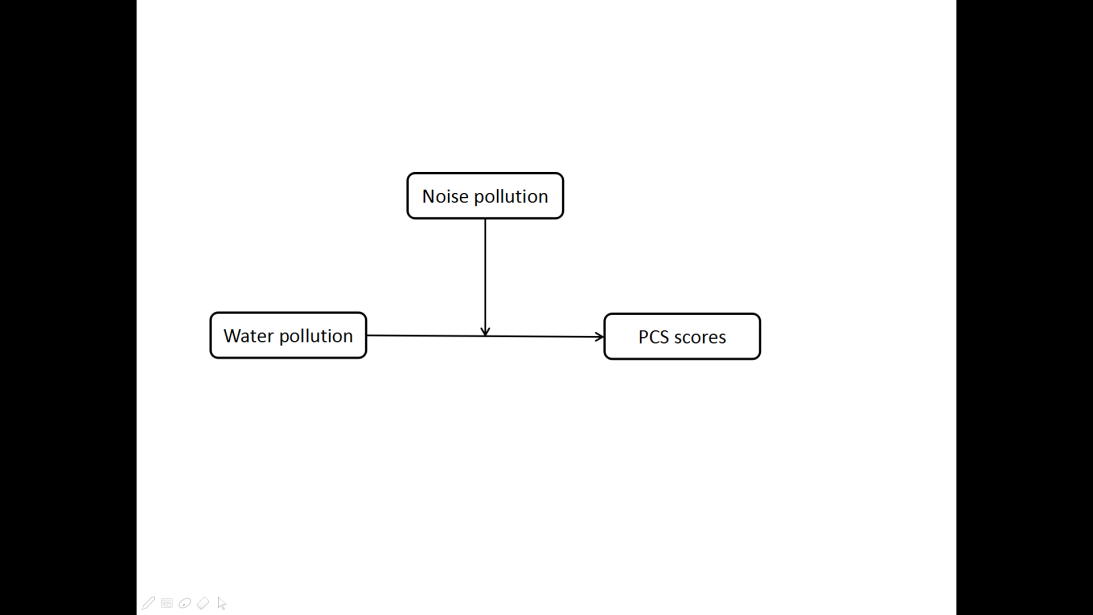


Figure 6. Conceptual diagram

Model = 1 Y = agg_phys X = water M = noise

Statistical Controls:CONTROL= employment bmi hhdnum religion alcohg smokg

Sample size 3697

**************************************************************************

Outcome: agg_phys

Model Summary

R R-sq MSE F df1 df2 p

.3159 .0998 108.0623 42.2529 9.0000 3687.0000 .0000

Model

coeff se t p LLCI ULCI

constant 43.4136 1.4024 30.9563 .0000 40.6640 46.1632

noise 1.2122 .3935 3.0803 .0021 .4406 1.9838

water -.7935 .4373 -1.8146 .0697 -1.6509 .0639

int_1 .5199 .8289 .6272 .5306 -1.1052 2.1450

employment -5.3598 .3941 -13.6004 .0000 -6.1325 -4.5872

bmi .1652 .0544 3.0339 .0024 .0584 .2719

hhdnum .7552 .1649 4.5790 .0000 .4318 1.0785

religion 1.4330 .5357 2.6749 .0075 .3827 2.4834

alcohg -.6985 .4094 -1.7063 .0880 -1.5011 .1041

smokg 3.4352 .3879 8.8551 .0000 2.6746 4.1958

Interactions:

int_1 water X noise

*************************************************************************

Conditional effect of X on Y at values of the moderator(s):

noise Effect se t p LLCI ULCI

-.3000 -.9495 .5688 -1.6691 .0952 -2.0647 .1658

.7000 -.4296 .6028 -.7126 .4761 -1.6114 .7523

**************************************************************************

Data for visualizing conditional effect of X on Y

Paste text below into a SPSS syntax window and execute to produce plot.

DATA LIST FREE/water noise agg_phys.

BEGIN DATA.

-.2659 -.3000 49.3177

.7341 -.3000 48.3682

-.2659 .7000 50.3916

.7341 .7000 49.9621

END DATA.

GRAPH/SCATTERPLOT=water WITH agg_phys BY noise.

* Estimates are based on setting covariates to their sample means.

******************** ANALYSIS NOTES AND WARNINGS *************************

**Statistical outcomes of potential conceptual diagram 7.**


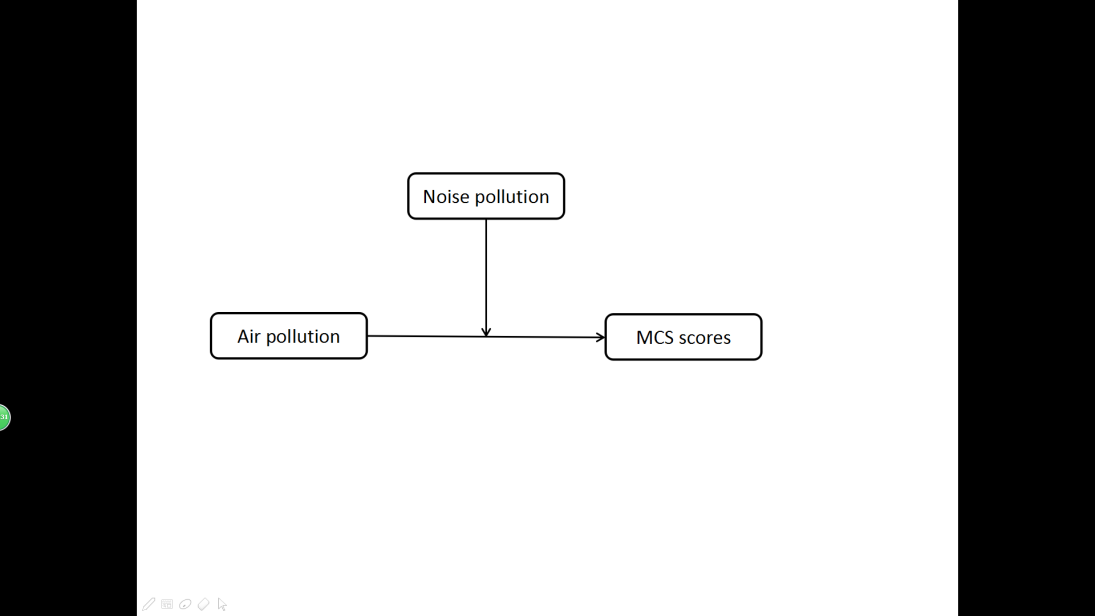


Figure 7. Conceptual diagram

Model = 1 Y = agg_ment X = air M = noise

Statistical Controls:CONTROL= employment bmi hhdnum religion alcohg smokg

Sample size 3701

**************************************************************************

Outcome: agg_ment

Model Summary

R R-sq MSE F df1 df2 p

.1407 .0198 86.8885 8.2180 9.0000 3691.0000 .0000

Model

coeff se t p LLCI ULCI

constant 42.5094 1.2076 35.2005 .0000 40.1417 44.8771

noise -.2411 .3943 -.6115 .5409 -1.0141 .5319

air -.6997 .3964 -1.7649 .0777 -1.4769 .0776

int_1 1.1854 .7722 1.5350 .1249 -.3287 2.6994

employment -.7567 .3379 -2.2392 .0252 -1.4192 -.0941

bmi .2526 .0465 5.4272 .0000 .1613 .3438

hhdnum -.0812 .1410 -.5759 .5647 -.3575 .1952

religion .5196 .4735 1.0975 .2725 -.4087 1.4479

alcohg .5088 .3688 1.3798 .1677 -.2142 1.2318

smokg 1.2380 .3530 3.5071 .0005 .5459 1.9301

Interactions:

int_1 air X noise

*************************************************************************

Conditional effect of X on Y at values of the moderator(s):

noise Effect se t p LLCI ULCI

-.3002 -1.0555 .5085 -2.0756 .0380 -2.0525 -.0585

.6998 .1299 .5814 .2234 .8233 -1.0100 1.2697

**************************************************************************

Data for visualizing conditional effect of X on Y

Paste text below into a SPSS syntax window and execute to produce plot.

DATA LIST FREE/air noise agg_ment.

BEGIN DATA.

-.2907 -.3002 49.1613

.7093 -.3002 48.1059

-.2907 .6998 48.5756

.7093 .6998 48.7055

END DATA.

GRAPH/SCATTERPLOT=air WITH agg_ment BY noise.

* Estimates are based on setting covariates to their sample means.

******************** ANALYSIS NOTES AND WARNINGS *************************

**Statistical outcomes of potential conceptual diagram 8.**


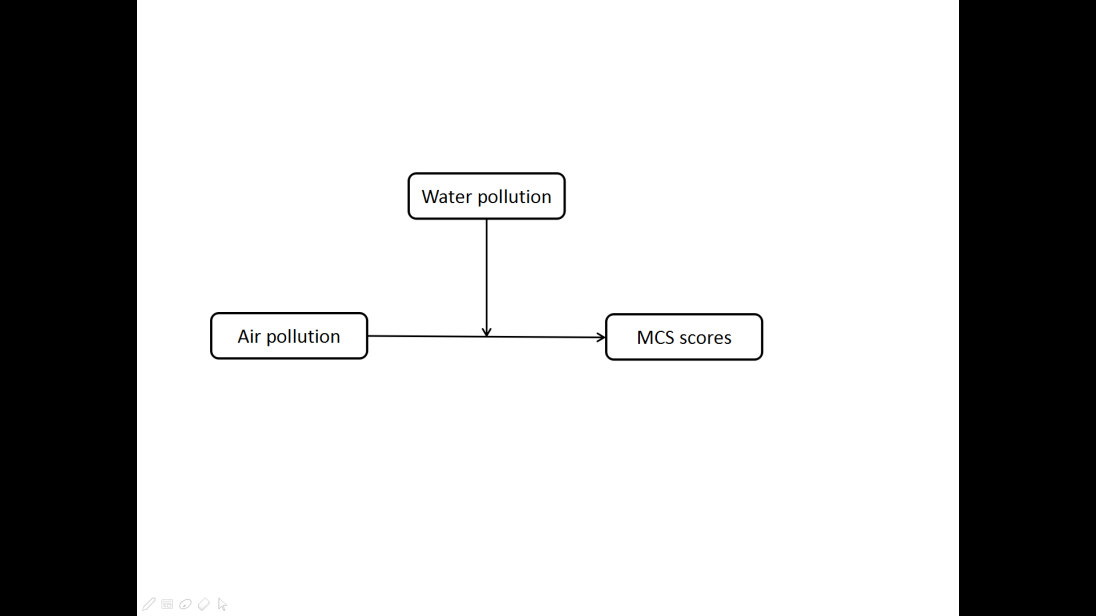


Figure 8. Conceptual diagram

Model = 1 Y = agg_ment X = air M = water

Statistical Controls:CONTROL= employment bmi hhdnum religion alcohg smokg

Sample size 3697

**************************************************************************

Outcome: agg_ment

Model Summary

R R-sq MSE F df1 df2 p

.1406 .0198 86.7315 8.2739 9.0000 3687.0000 .0000

Model

coeff se t p LLCI ULCI

constant 42.6005 1.2095 35.2203 .0000 40.2291 44.9720

water -.6463 .4449 -1.4527 .1464 -1.5185 .2260

air -.4553 .4011 -1.1350 .2565 -1.2418 .3312

int_1 .8471 .8192 1.0341 .3012 -.7590 2.4532

employment -.7895 .3377 -2.3378 .0194 -1.4517 -.1274

bmi .2507 .0464 5.4014 .0000 .1597 .3417

hhdnum -.0760 .1411 -.5385 .5903 -.3526 .2007

religion .4838 .4743 1.0200 .3078 -.4461 1.4137

alcohg .5310 .3695 1.4371 .1508 -.1934 1.2554

smokg 1.2159 .3522 3.4522 .0006 .5254 1.9064

Interactions:

int_1 air X water

*************************************************************************

Conditional effect of X on Y at values of the moderator(s):

water Effect se t p LLCI ULCI

-.2659 -.6805 .4908 -1.3866 .1656 -1.6428 .2817

.7341 .1666 .6579 .2532 .8001 -1.1232 1.4564

**************************************************************************

Data for visualizing conditional effect of X on Y

Paste text below into a SPSS syntax window and execute to produce plot.

DATA LIST FREE/air water agg_ment.

BEGIN DATA.

-.2905 -.2659 49.1679

.7095 -.2659 48.4874

-.2905 .7341 48.2755

.7095 .7341 48.4421

END DATA.

GRAPH/SCATTERPLOT=air WITH agg_ment BY water.

* Estimates are based on setting covariates to their sample means.

******************** ANALYSIS NOTES AND WARNINGS *************************

**Statistical outcomes of potential conceptual diagram 9.**


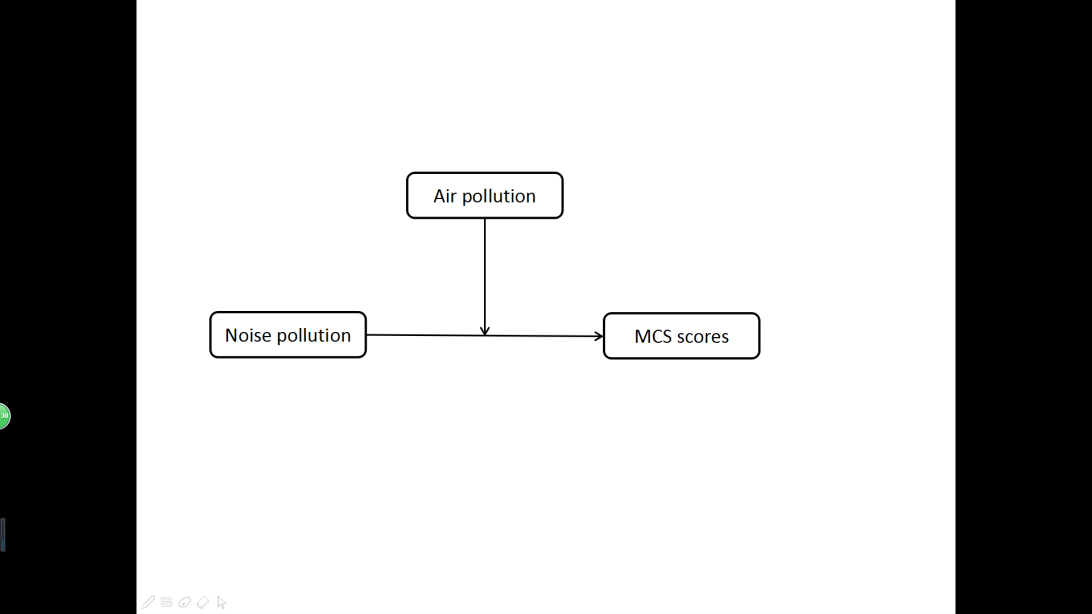


Figure 9. Conceptual diagram

Model = 1 Y = agg_ment X = noise M = air

Statistical Controls:CONTROL= employment bmi hhdnum religion alcohg smokg

Sample size 3701

**************************************************************************

Outcome: agg_ment

Model Summary

R R-sq MSE F df1 df2 p

.1407 .0198 86.8885 8.2180 9.0000 3691.0000 .0000

Model

coeff se t p LLCI ULCI

constant 42.5094 1.2076 35.2005 .0000 40.1417 44.8771

air -.6997 .3964 -1.7649 .0777 -1.4769 .0776

noise -.2411 .3943 -.6115 .5409 -1.0141 .5319

int_1 1.1854 .7722 1.5350 .1249 -.3287 2.6994

employment -.7567 .3379 -2.2392 .0252 -1.4192 -.0941

bmi .2526 .0465 5.4272 .0000 .1613 .3438

hhdnum -.0812 .1410 -.5759 .5647 -.3575 .1952

religion .5196 .4735 1.0975 .2725 -.4087 1.4479

alcohg .5088 .3688 1.3798 .1677 -.2142 1.2318

smokg 1.2380 .3530 3.5071 .0005 .5459 1.9301

Interactions:

int_1 noise X air

*************************************************************************

Conditional effect of X on Y at values of the moderator(s):

air Effect se t p LLCI ULCI

-.2907 -.5857 .5020 -1.1669 .2433 -1.5699 .3984

.7093 .5996 .5856 1.0240 .3059 -.5485 1.7478

**************************************************************************

Data for visualizing conditional effect of X on Y

Paste text below into a SPSS syntax window and execute to produce plot.

DATA LIST FREE/noise air agg_ment.

BEGIN DATA.

-.3002 -.2907 49.1613

.6998 -.2907 48.5756

-.3002 .7093 48.1059

.6998 .7093 48.7055

END DATA.

GRAPH/SCATTERPLOT=noise WITH agg_ment BY air.

* Estimates are based on setting covariates to their sample means.

******************** ANALYSIS NOTES AND WARNINGS *************************

**Statistical outcomes of potential conceptual diagram 10.**


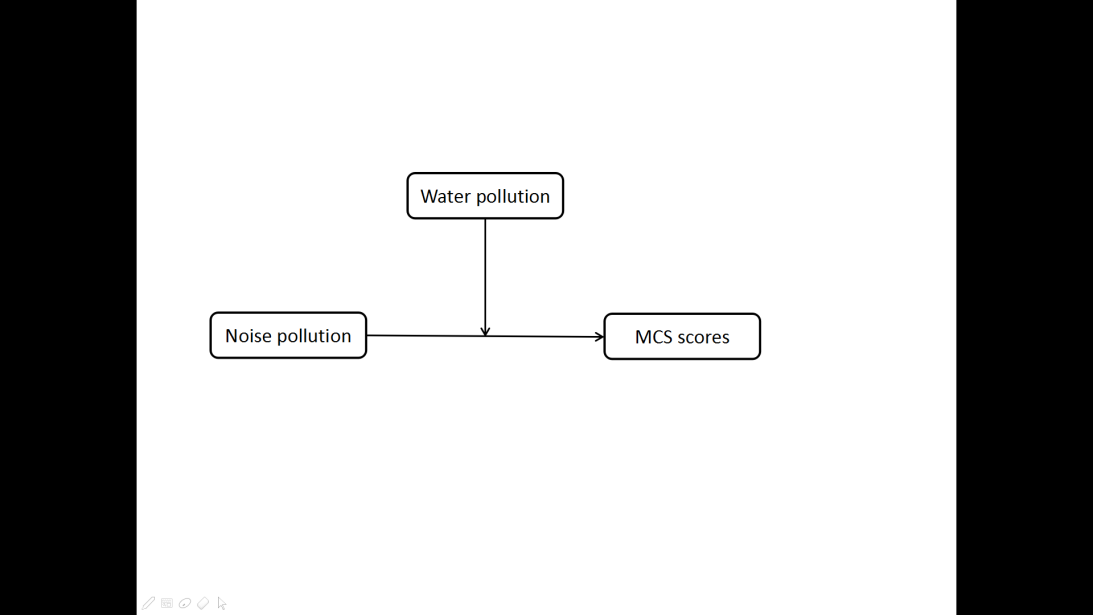


Figure 10. Conceptual diagram

Model = 1 Y = agg_ment X = noise M = water

Statistical Controls:CONTROL= employment bmi hhdnum religion alcohg smokg

Sample size 3697

**************************************************************************

Outcome: agg_ment

Model Summary

R R-sq MSE F df1 df2 p

.1391 .0194 86.7678 8.1396 9.0000 3687.0000 .0000

Model

coeff se t p LLCI ULCI

constant 42.7542 1.2020 35.5693 .0000 40.3975 45.1108

water -.6561 .3825 -1.7151 .0864 -1.4061 .0939

noise -.1902 .3571 -.5326 .5943 -.8903 .5100

int_1 .2791 .7484 .3730 .7092 -1.1882 1.7465

employment -.7913 .3374 -2.3456 .0190 -1.4528 -.1299

bmi .2473 .0463 5.3459 .0000 .1566 .3380

hhdnum -.0716 .1411 -.5074 .6119 -.3483 .2051

religion .4574 .4743 .9643 .3350 -.4726 1.3874

alcohg .5409 .3692 1.4653 .1429 -.1829 1.2647

smokg 1.2150 .3534 3.4381 .0006 .5221 1.9078

Interactions:

int_1 noise X water

*************************************************************************

Conditional effect of X on Y at values of the moderator(s):

water Effect se t p LLCI ULCI

-.2659 -.2644 .4348 -.6081 .5432 -1.1169 .5881

.7341 .0147 .6073 .0242 .9807 -1.1759 1.2054

**************************************************************************

Data for visualizing conditional effect of X on Y

Paste text below into a SPSS syntax window and execute to produce plot.

DATA LIST FREE/noise water agg_ment.

BEGIN DATA.

-.3000 -.2659 49.1200

.7000 -.2659 48.8556

-.3000 .7341 48.3802

.7000 .7341 48.3949

END DATA.

GRAPH/SCATTERPLOT=noise WITH agg_ment BY water.

* Estimates are based on setting covariates to their sample means.

******************** ANALYSIS NOTES AND WARNINGS *************************

**Statistical outcomes of potential conceptual diagram 11.**


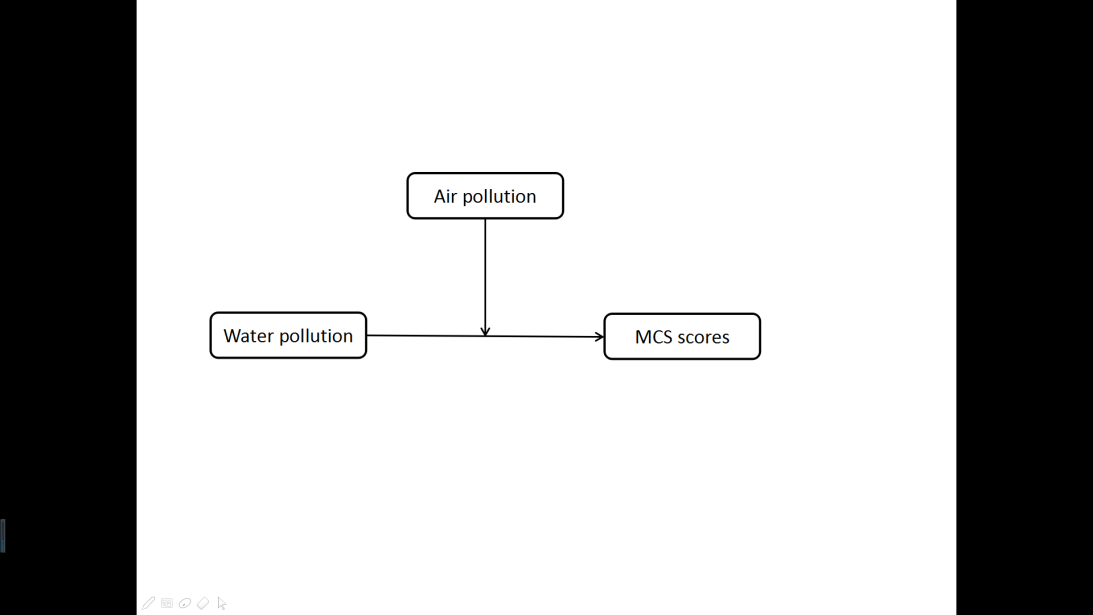


Figure 11. Conceptual diagram

Model = 1 Y = agg_ment X = water M = air

Statistical Controls:CONTROL= employment bmi hhdnum religion alcohg smokg

Sample size 3697

**************************************************************************

Outcome: agg_ment

Model Summary

R R-sq MSE F df1 df2 p

.1406 .0198 86.7315 8.2739 9.0000 3687.0000 .0000

Model

coeff se t p LLCI ULCI

constant 42.6005 1.2095 35.2203 .0000 40.2291 44.9720

air -.4553 .4011 -1.1350 .2565 -1.2418 .3312

water -.6463 .4449 -1.4527 .1464 -1.5185 .2260

int_1 .8471 .8192 1.0341 .3012 -.7590 2.4532

employment -.7895 .3377 -2.3378 .0194 -1.4517 -.1274

bmi .2507 .0464 5.4014 .0000 .1597 .3417

hhdnum -.0760 .1411 -.5385 .5903 -.3526 .2007

religion .4838 .4743 1.0200 .3078 -.4461 1.4137

alcohg .5310 .3695 1.4371 .1508 -.1934 1.2554

smokg 1.2159 .3522 3.4522 .0006 .5254 1.9064

Interactions:

int_1 water X air

*************************************************************************

Conditional effect of X on Y at values of the moderator(s):

air Effect se t p LLCI ULCI

-.2905 -.8924 .5808 -1.5365 .1245 -2.0311 .2463

.7095 -.0453 .5775 -.0784 .9375 -1.1776 1.0871

**************************************************************************

Data for visualizing conditional effect of X on Y

Paste text below into a SPSS syntax window and execute to produce plot.

DATA LIST FREE/water air agg_ment.

BEGIN DATA.

-.2659 -.2905 49.1679

.7341 -.2905 48.2755

-.2659 .7095 48.4874

.7341 .7095 48.4421

END DATA.

GRAPH/SCATTERPLOT=water WITH agg_ment BY air.

* Estimates are based on setting covariates to their sample means.

******************** ANALYSIS NOTES AND WARNINGS *************************

**Statistical outcomes of potential conceptual diagram 12.**


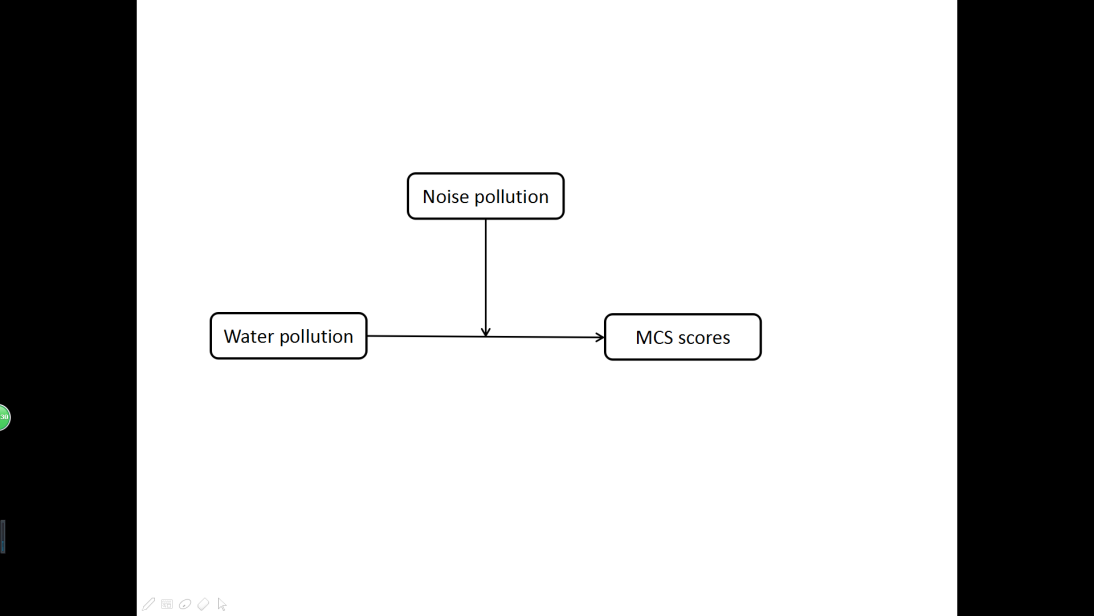


Figure 12. Conceptual diagram

Model = 1 Y = agg_ment X = water M = noise

Statistical Controls:CONTROL= employment bmi hhdnum religion alcohg smokg

Sample size 3697

**************************************************************************

Outcome: agg_ment

Model Summary

R R-sq MSE F df1 df2 p

.1391 .0194 86.7678 8.1396 9.0000 3687.0000 .0000

Model

coeff se t p LLCI ULCI

constant 42.7542 1.2020 35.5693 .0000 40.3975 45.1108

noise -.1902 .3571 -.5326 .5943 -.8903 .5100

water -.6561 .3825 -1.7151 .0864 -1.4061 .0939

int_1 .2791 .7484 .3730 .7092 -1.1882 1.7465

employment -.7913 .3374 -2.3456 .0190 -1.4528 -.1299

bmi .2473 .0463 5.3459 .0000 .1566 .3380

hhdnum -.0716 .1411 -.5074 .6119 -.3483 .2051

religion .4574 .4743 .9643 .3350 -.4726 1.3874

alcohg .5409 .3692 1.4653 .1429 -.1829 1.2647

smokg 1.2150 .3534 3.4381 .0006 .5221 1.9078

Interactions:

int_1 water X noise

*************************************************************************

Conditional effect of X on Y at values of the moderator(s):

noise Effect se t p LLCI ULCI

-.3000 -.7399 .4916 -1.5049 .1324 -1.7038 .2241

.7000 -.4607 .5620 -.8197 .4124 -1.5627 .6412

**************************************************************************

Data for visualizing conditional effect of X on Y

Paste text below into a SPSS syntax window and execute to produce plot.

DATA LIST FREE/water noise agg_ment.

BEGIN DATA.

-.2659 -.3000 49.1200

.7341 -.3000 48.3802

-.2659 .7000 48.8556

.7341 .7000 48.3949

END DATA.

GRAPH/SCATTERPLOT=water WITH agg_ment BY noise.

* Estimates are based on setting covariates to their sample means.

******************** ANALYSIS NOTES AND WARNINGS *************************

**Statistical outcomes of potential conceptual diagram 13.**


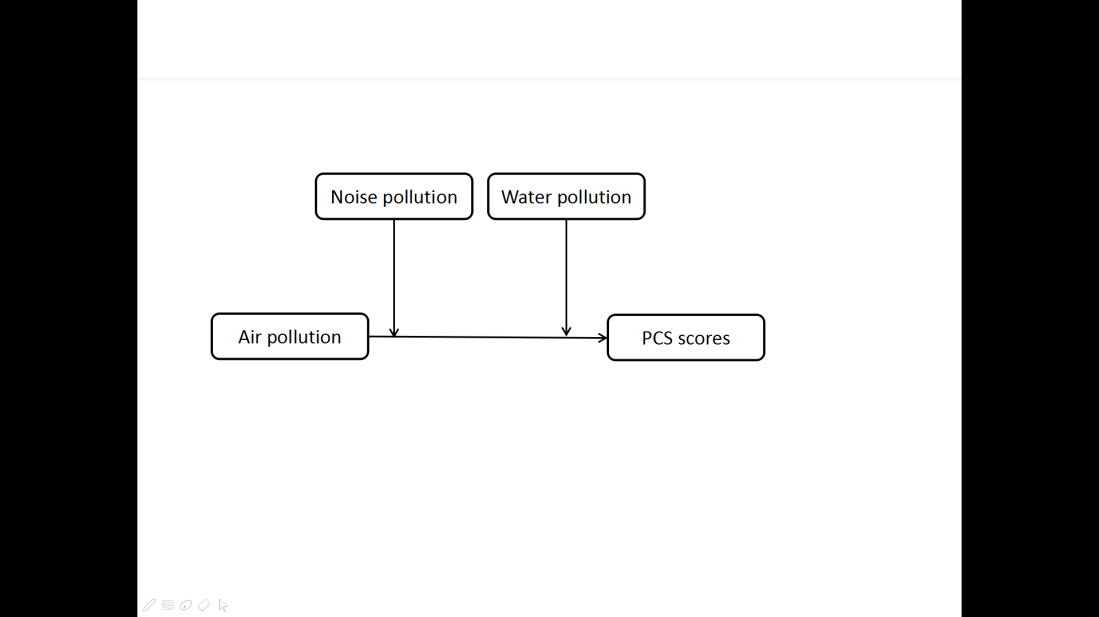


Figure 13. Conceptual diagram

Model = 2

Y = agg_phys X = air M = noise W = water

Statistical Controls:

CONTROL= employment bmi hhdnum religion alcohg smokg

Sample size 3697

**************************************************************************

Outcome: agg_phys

Model Summary

R R-sq MSE F df1 df2 p

.32 .10 107.89 37.91 11.00 3685.00 .00

Model

coeff se t p LLCI ULCI

constant 43.33 1.32 32.84 .00 40.74 45.92

noise 1.17 .44 2.65 .01 .30 2.03

air .63 .50 1.27 .20 -.34 1.61

int_1 -1.30 .87 -1.49 .14 -3.00 .41

water -1.34 .48 -2.76 .01 -2.29 -.39

int_2 1.96 .91 2.15 .03 .17 3.74

employment -5.38 .37 -14.70 .00 -6.10 -4.67

bmi .17 .05 3.30 .00 .07 .27

hhdnum .76 .16 4.81 .00 .45 1.08

religion 1.42 .52 2.72 .01 .40 2.44

alcohg -.69 .42 -1.64 .10 -1.51 .13

smokg 3.42 .40 8.50 .00 2.63 4.20

Interactions:

int_1 air X noise

int_2 air X water

R-square increase due to interaction(s):

R2-chng F df1 df2 p

int_1 .00 2.21 1.00 3685.00 .14

int_2 .00 4.61 1.00 3685.00 .03

Both .00 3.04 2.00 3685.00 .05

*************************************************************************

Conditional effect of X on Y at values of the moderator(s):

water noise Effect se t p LLCI ULCI

-.27 -.30 .50 .67 .74 .46 -.82 1.82

-.27 .70 -.80 .80 -1.00 .32 -2.36 .76

.73 -.30 2.46 .86 2.85 .00 .76 4.15

.73 .70 1.16 .84 1.37 .17 -.49 2.82

**************************************************************************

Data for visualizing conditional effect of X on Y

Paste text below into a SPSS syntax window and execute to produce plot.

DATA LIST FREE/air water noise agg_phys.

BEGIN DATA.

-.29 -.27 -.30 49.25

.71 -.27 -.30 49.75

-.29 -.27 .70 50.80

.71 -.27 .70 50.00

-.29 .73 -.30 47.35

.71 .73 -.30 49.80

-.29 .73 .70 48.89

.71 .73 .70 50.05

**Statistical outcomes of potential conceptual diagram 14.**


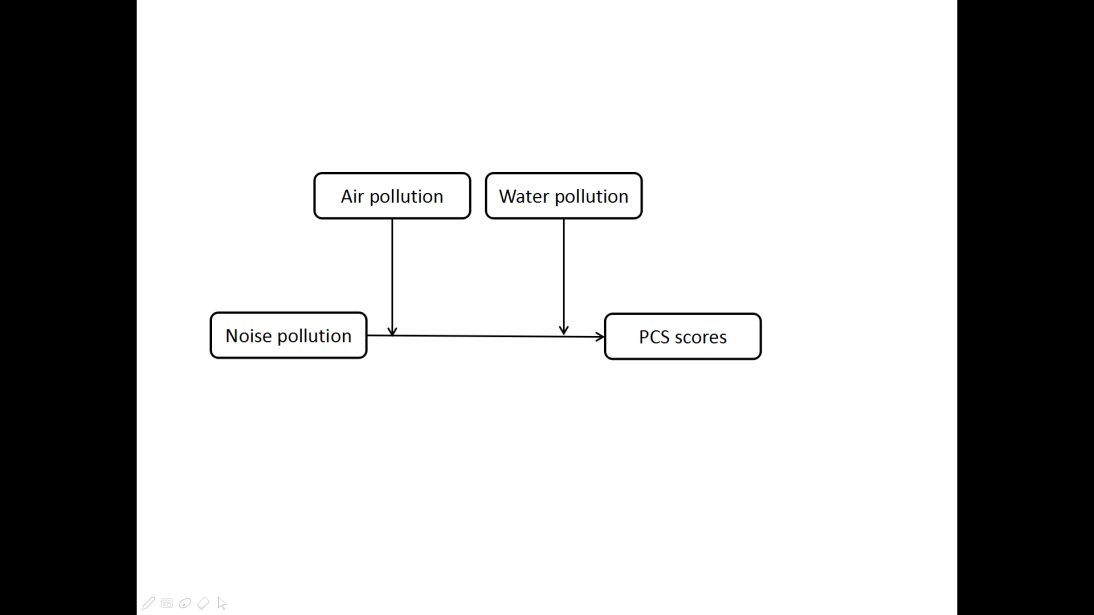


Figure 14. Conceptual diagram

Model = 2 Y = agg_phys X = noise M = air W = water

Statistical Controls:

CONTROL= employment bmi hhdnum religion alcohg smokg

Sample size 3697

**************************************************************************

Outcome: agg_phys

Model Summary

R R-sq MSE F df1 df2 p

.32 .10 107.99 37.59 11.00 3685.00 .00

Model

coeff se t p LLCI ULCI

constant 43.57 1.31 33.16 .00 40.99 46.14

air .86 .49 1.76 .08 -.10 1.82

noise 1.13 .44 2.55 .01 .26 1.99

int_1 -1.55 .96 -1.62 .11 -3.43 .33

water -1.11 .47 -2.38 .02 -2.03 -.19

int_2 1.12 .92 1.21 .23 -.69 2.93

employment -5.38 .37 -14.69 .00 -6.10 -4.66

bmi .16 .05 3.24 .00 .06 .26

hhdnum .77 .16 4.81 .00 .45 1.08

religion 1.39 .52 2.65 .01 .36 2.41

alcohg -.68 .42 -1.62 .11 -1.51 .14

smokg 3.42 .40 8.50 .00 2.63 4.21

Interactions:

int_1 noise X air

int_2 noise X water

R-square increase due to interaction(s):

R2-chng F df1 df2 p

int_1 .00 2.61 1.00 3685.00 .11

int_2 .00 1.47 1.00 3685.00 .23

Both .00 1.47 2.00 3685.00 .23

*************************************************************************

Conditional effect of X on Y at values of the moderator(s):

water air Effect se t p LLCI ULCI

-.27 -.29 1.28 .58 2.22 .03 .15 2.41

-.27 .71 -.27 .87 -.31 .76 -1.98 1.44

.73 -.29 2.40 .96 2.51 .01 .53 4.27

.73 .71 .85 .76 1.12 .26 -.63 2.33

**************************************************************************

Data for visualizing conditional effect of X on Y

Paste text below into a SPSS syntax window and execute to produce plot.

DATA LIST FREE/noise water air agg_phys.

BEGIN DATA.

-.30 -.27 -.29 49.20

.70 -.27 -.29 50.48

-.30 -.27 .71 50.53

.70 -.27 .71 50.26

-.30 .73 -.29 47.75

.70 .73 -.29 50.15

-.30 .73 .71 49.08

.70 .73 .71 49.93

**Statistical outcomes of potential conceptual diagram 15.**


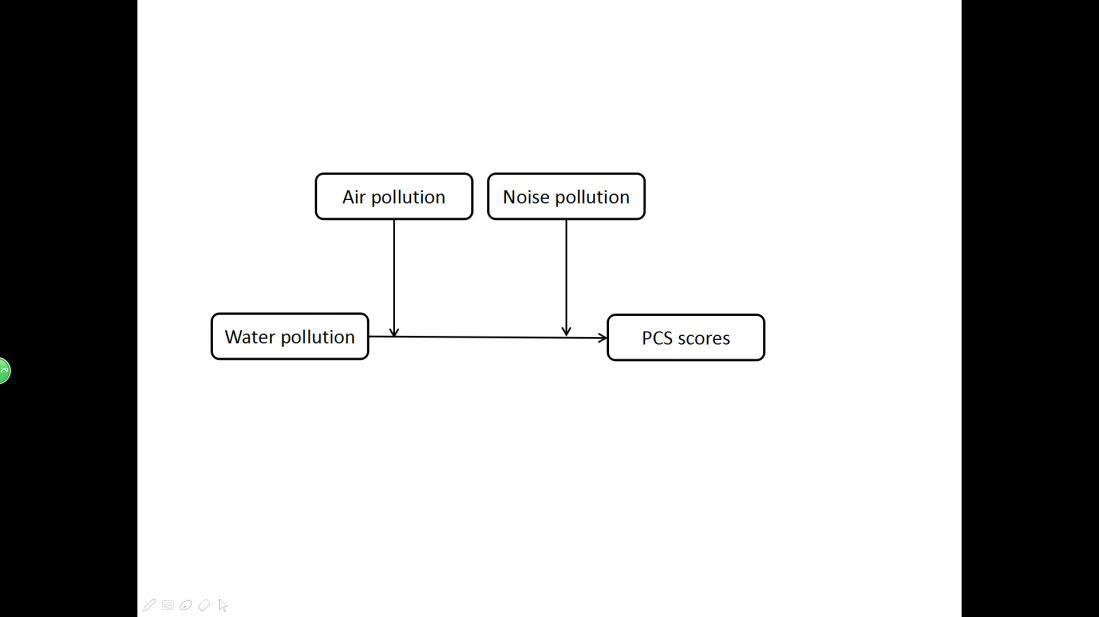


Figure 15. Conceptual diagram

Model = 2 Y = agg_phys X = water M = air W = noise

Statistical Controls:CONTROL= employment bmi hhdnum religion alcohg smokg

Sample size 3697

**************************************************************************

Outcome: agg_phys

Model Summary

R R-sq MSE F df1 df2 p

.32 .10 107.96 37.69 11.00 3685.00 .00

Model

coeff se t p LLCI ULCI

constant 43.28 1.32 32.81 .00 40.69 45.86

air .47 .49 .97 .33 -.48 1.43

water -1.32 .49 -2.71 .01 -2.27 -.36

int_1 1.86 .98 1.90 .06 -.06 3.79

noise 1.03 .43 2.37 .02 .18 1.88

int_2 -.20 .91 -.22 .82 -1.98 1.57

employment -5.37 .37 -14.68 .00 -6.09 -4.66

bmi .16 .05 3.25 .00 .07 .26

hhdnum .76 .16 4.80 .00 .45 1.07

religion 1.42 .52 2.71 .01 .39 2.45

alcohg -.69 .42 -1.64 .10 -1.52 .13

smokg 3.43 .40 8.52 .00 2.64 4.22

Interactions:

int_1 water X air

int_2 water X noise

R-square increase due to interaction(s):

R2-chng F df1 df2 p

int_1 .00 3.60 1.00 3685.00 .06

int_2 .00 .05 1.00 3685.00 .82

Both .00 1.96 2.00 3685.00 .14

*************************************************************************

Conditional effect of X on Y at values of the moderator(s):

noise air Effect se t p LLCI ULCI

-.30 -.29 -1.80 .66 -2.74 .01 -3.09 -.51

-.30 .71 .06 .88 .07 .94 -1.67 1.79

.70 -.29 -2.00 .94 -2.13 .03 -3.85 -.16

.70 .71 -.14 .73 -.19 .85 -1.57 1.29

**************************************************************************

Data for visualizing conditional effect of X on Y

Paste text below into a SPSS syntax window and execute to produce plot.

DATA LIST FREE/water noise air agg_phys.

BEGIN DATA.

-.27 -.30 -.29 49.32

.73 -.30 -.29 47.52

-.27 -.30 .71 49.30

.73 -.30 .71 49.36

-.27 .70 -.29 50.40

.73 .70 -.29 48.40

-.27 .70 .71 50.38

.73 .70 .71 50.24

**Statistical outcomes of potential conceptual diagram 16.**


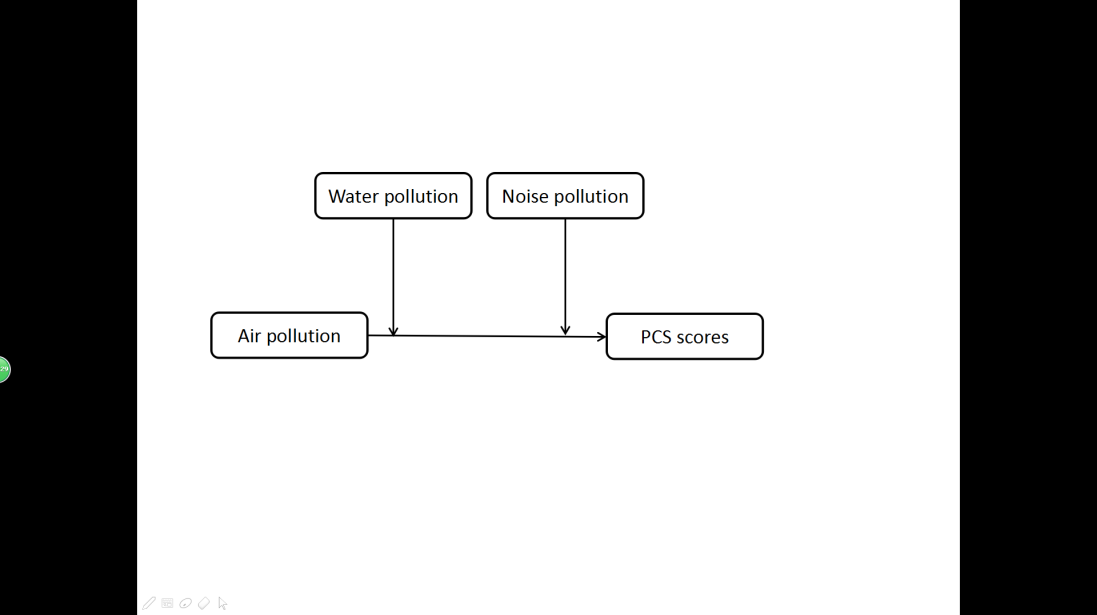


Figure 16. Conceptual diagram

Model = 2 Y = agg_phys X = air M = water W = noise

Statistical Controls:CONTROL= employment bmi hhdnum religion alcohg smokg

Sample size 3697

**************************************************************************

Outcome: agg_phys

Model Summary

R R-sq MSE F df1 df2 p

.32 .10 107.89 37.91 11.00 3685.00 .00

Model

coeff se t p LLCI ULCI

constant 43.33 1.32 32.84 .00 40.74 45.92

water -1.34 .48 -2.76 .01 -2.29 -.39

air .63 .50 1.27 .20 -.34 1.61

int_1 1.96 .91 2.15 .03 .17 3.74

noise 1.17 .44 2.65 .01 .30 2.03

int_2 -1.30 .87 -1.49 .14 -3.00 .41

employment -5.38 .37 -14.70 .00 -6.10 -4.67

bmi .17 .05 3.30 .00 .07 .27

hhdnum .76 .16 4.81 .00 .45 1.08

religion 1.42 .52 2.72 .01 .40 2.44

alcohg -.69 .42 -1.64 .10 -1.51 .13

smokg 3.42 .40 8.50 .00 2.63 4.20

Interactions:

int_1 air X water

int_2 air X noise

R-square increase due to interaction(s):

R2-chng F df1 df2 p

int_1 .00 4.61 1.00 3685.00 .03

int_2 .00 2.21 1.00 3685.00 .14

Both .00 3.04 2.00 3685.00 .05

*************************************************************************

Conditional effect of X on Y at values of the moderator(s):

noise water Effect se t p LLCI ULCI

-.30 -.27 .50 .67 .74 .46 -.82 1.82

-.30 .73 2.46 .86 2.85 .00 .76 4.15

.70 -.27 -.80 .80 -1.00 .32 -2.36 .76

.70 .73 1.16 .84 1.37 .17 -.49 2.82

**************************************************************************

Data for visualizing conditional effect of X on Y

Paste text below into a SPSS syntax window and execute to produce plot.

DATA LIST FREE/air noise water agg_phys.

BEGIN DATA.

-.29 -.30 -.27 49.25

.71 -.30 -.27 49.75

-.29 -.30 .73 47.35

.71 -.30 .73 49.80

-.29 .70 -.27 50.80

.71 .70 -.27 50.00

-.29 .70 .73 48.89

.71 .70 .73 50.05

**Statistical outcomes of potential conceptual diagram 17.**


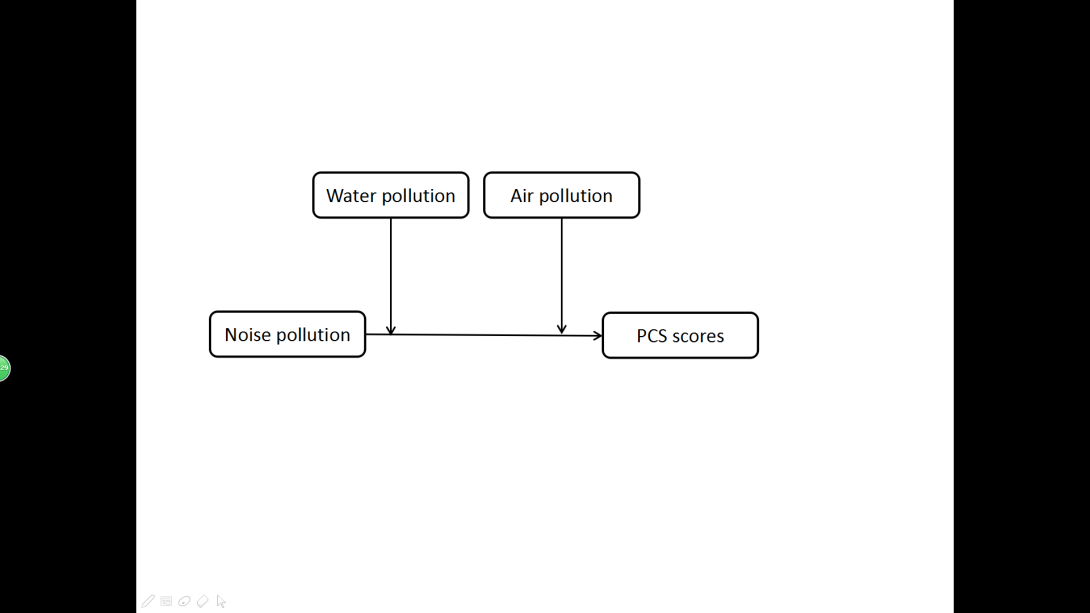


Figure 17. Conceptual diagram

Model = 2 Y = agg_phys X = noise M = water W = air

Statistical Controls:CONTROL= employment bmi hhdnum religion alcohg smokg

Sample size 3697

**************************************************************************

Outcome: agg_phys

Model Summary

R R-sq MSE F df1 df2 p

.32 .10 107.99 37.59 11.00 3685.00 .00

Model

coeff se t p LLCI ULCI

constant 43.57 1.31 33.16 .00 40.99 46.14

water -1.11 .47 -2.38 .02 -2.03 -.19

noise 1.13 .44 2.55 .01 .26 1.99

int_1 1.12 .92 1.21 .23 -.69 2.93

air .86 .49 1.76 .08 -.10 1.82

int_2 -1.55 .96 -1.62 .11 -3.43 .33

employment -5.38 .37 -14.69 .00 -6.10 -4.66

bmi .16 .05 3.24 .00 .06 .26

hhdnum .77 .16 4.81 .00 .45 1.08

religion 1.39 .52 2.65 .01 .36 2.41

alcohg -.68 .42 -1.62 .11 -1.51 .14

smokg 3.42 .40 8.50 .00 2.63 4.21

Interactions:

int_1 noise X water

int_2 noise X air

R-square increase due to interaction(s):

R2-chng F df1 df2 p

int_1 .00 1.47 1.00 3685.00 .23

int_2 .00 2.61 1.00 3685.00 .11

Both .00 1.47 2.00 3685.00 .23

*************************************************************************

Conditional effect of X on Y at values of the moderator(s):

air water Effect se t p LLCI ULCI

-.29 -.27 1.28 .58 2.22 .03 .15 2.41

-.29 .73 2.40 .96 2.51 .01 .53 4.27

.71 -.27 -.27 .87 -.31 .76 -1.98 1.44

.71 .73 .85 .76 1.12 .26 -.63 2.33

**************************************************************************

Data for visualizing conditional effect of X on Y

Paste text below into a SPSS syntax window and execute to produce plot.

DATA LIST FREE/noise air water agg_phys.

BEGIN DATA.

-.30 -.29 -.27 49.20

.70 -.29 -.27 50.48

-.30 -.29 .73 47.75

.70 -.29 .73 50.15

-.30 .71 -.27 50.53

.70 .71 -.27 50.26

-.30 .71 .73 49.08

.70 .71 .73 49.93

**Statistical outcomes of potential conceptual diagram 18.**


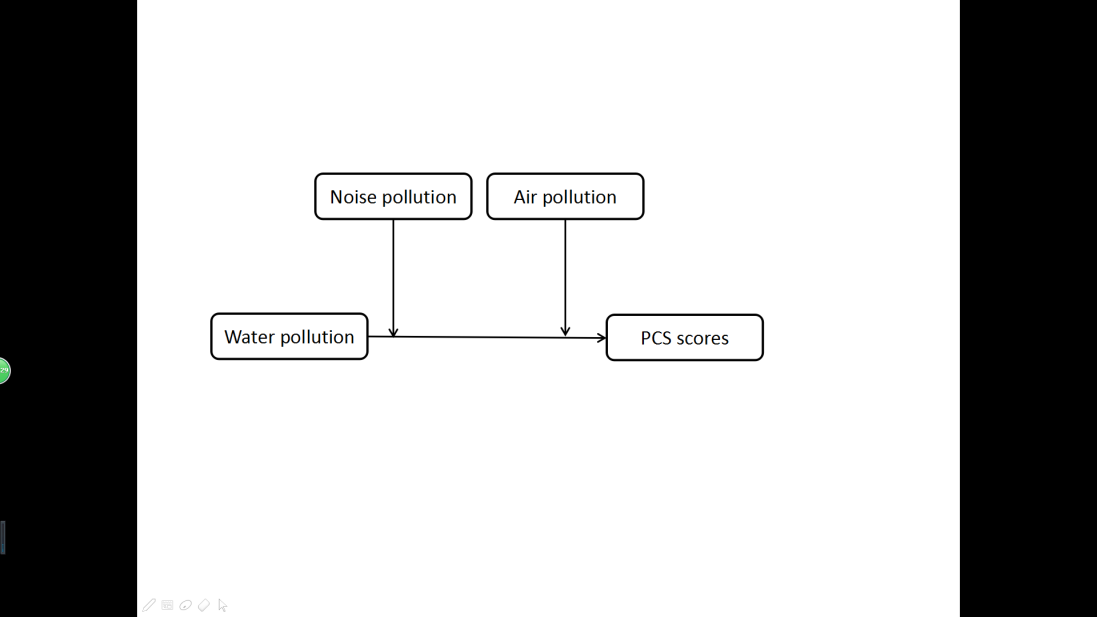


Figure 18. Conceptual diagram

Model = 2 Y = agg_phys X = water M = noise W = air

Statistical Controls:

CONTROL= employment bmi hhdnum religion alcohg smokg

Sample size 3697

**************************************************************************

Outcome: agg_phys

Model Summary

R R-sq MSE F df1 df2 p

.32 .10 107.96 37.69 11.00 3685.00 .00

Model

coeff se t p LLCI ULCI

constant 43.28 1.32 32.81 .00 40.69 45.86

noise 1.03 .43 2.37 .02 .18 1.88

water -1.32 .49 -2.71 .01 -2.27 -.36

int_1 -.20 .91 -.22 .82 -1.98 1.57

air .47 .49 .97 .33 -.48 1.43

int_2 1.86 .98 1.90 .06 -.06 3.79

employment -5.37 .37 -14.68 .00 -6.09 -4.66

bmi .16 .05 3.25 .00 .07 .26

hhdnum .76 .16 4.80 .00 .45 1.07

religion 1.42 .52 2.71 .01 .39 2.45

alcohg -.69 .42 -1.64 .10 -1.52 .13

smokg 3.43 .40 8.52 .00 2.64 4.22

Interactions:

int_1 water X noise

int_2 water X air

R-square increase due to interaction(s):

R2-chng F df1 df2 p

int_1 .00 .05 1.00 3685.00 .82

int_2 .00 3.60 1.00 3685.00 .06

Both .00 1.96 2.00 3685.00 .14

*************************************************************************

Conditional effect of X on Y at values of the moderator(s):

air noise Effect se t p LLCI ULCI

-.29 -.30 -1.80 .66 -2.74 .01 -3.09 -.51

-.29 .70 -2.00 .94 -2.13 .03 -3.85 -.16

.71 -.30 .06 .88 .07 .94 -1.67 1.79

.71 .70 -.14 .73 -.19 .85 -1.57 1.29

**************************************************************************

Data for visualizing conditional effect of X on Y

Paste text below into a SPSS syntax window and execute to produce plot.

DATA LIST FREE/water air noise agg_phys.

BEGIN DATA.

-.27 -.29 -.30 49.32

.73 -.29 -.30 47.52

-.27 -.29 .70 50.40

.73 -.29 .70 48.40

-.27 .71 -.30 49.30

.73 .71 -.30 49.36

-.27 .71 .70 50.38

.73 .71 .70 50.24

**Statistical outcomes of potential conceptual diagram 19.**


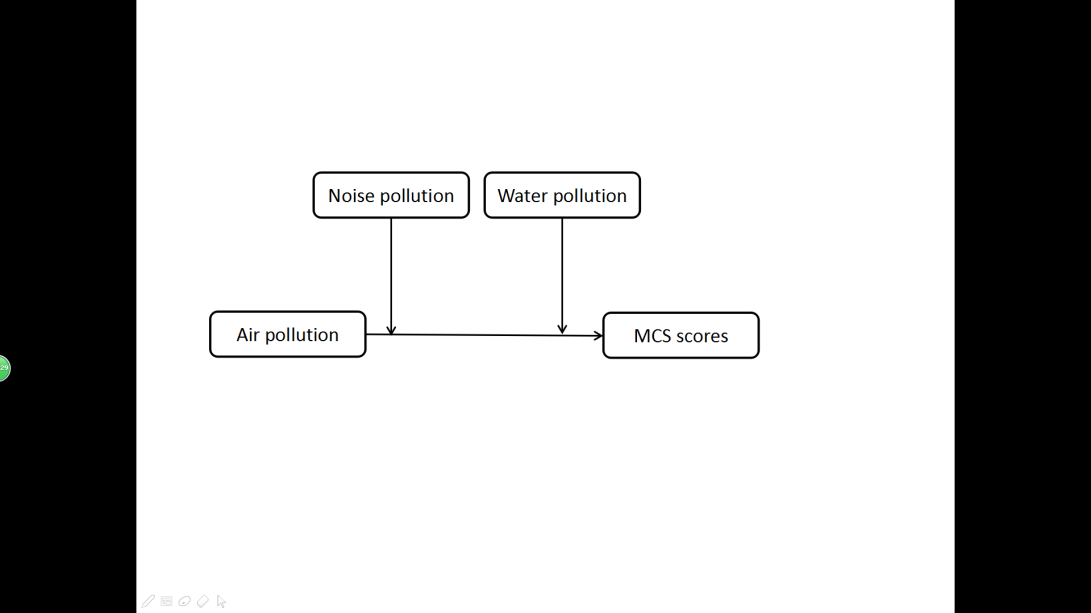


Figure 19. Conceptual diagram

Model = 2 Y = agg_ment X = air M = noise W = water

Statistical Controls:CONTROL= employment bmi hhdnum religion alcohg smokg

Sample size

3697

**************************************************************************

Outcome: agg_ment

Model Summary

R R-sq MSE F df1 df2 p

.14 .02 86.73 6.93 11.00 3685.00 .00

Model

coeff se t p LLCI ULCI

constant 42.55 1.18 35.97 .00 40.23 44.87

noise -.19 .40 -.48 .63 -.97 .59

air -.56 .45 -1.25 .21 -1.43 .32

int_1 1.07 .78 1.37 .17 -.46 2.60

water -.63 .43 -1.45 .15 -1.48 .22

int_2 .70 .82 .86 .39 -.90 2.30

employment -.78 .33 -2.38 .02 -1.42 -.14

bmi .25 .05 5.47 .00 .16 .34

hhdnum -.08 .14 -.54 .59 -.36 .20

religion .48 .47 1.02 .31 -.44 1.40

alcohg .53 .38 1.40 .16 -.21 1.27

smokg 1.23 .36 3.42 .00 .53 1.94

Interactions:

int_1 air X noise

int_2 air X water

R-square increase due to interaction(s):

R2-chng F df1 df2 p

int_1 .00 1.87 1.00 3685.00 .17

int_2 .00 .73 1.00 3685.00 .39

Both .00 1.49 2.00 3685.00 .23

*************************************************************************

Conditional effect of X on Y at values of the moderator(s):

water noise Effect se t p LLCI ULCI

-.27 -.30 -1.06 .60 -1.76 .08 -2.25 .12

-.27 .70 .01 .71 .01 .99 -1.39 1.41

.73 -.30 -.36 .77 -.47 .64 -1.88 1.15

.73 .70 .71 .76 .93 .35 -.78 2.19

**************************************************************************

Data for visualizing conditional effect of X on Y

Paste text below into a SPSS syntax window and execute to produce plot.

DATA LIST FREE/air water noise agg_ment.

BEGIN DATA.

-.29 -.27 -.30 49.24

.71 -.27 -.30 48.18

-.29 -.27 .70 48.74

.71 -.27 .70 48.75

-.29 .73 -.30 48.41

.71 .73 -.30 48.04

-.29 .73 .70 47.91

.71 .73 .70 48.61

**Statistical outcomes of potential conceptual diagram 20.**


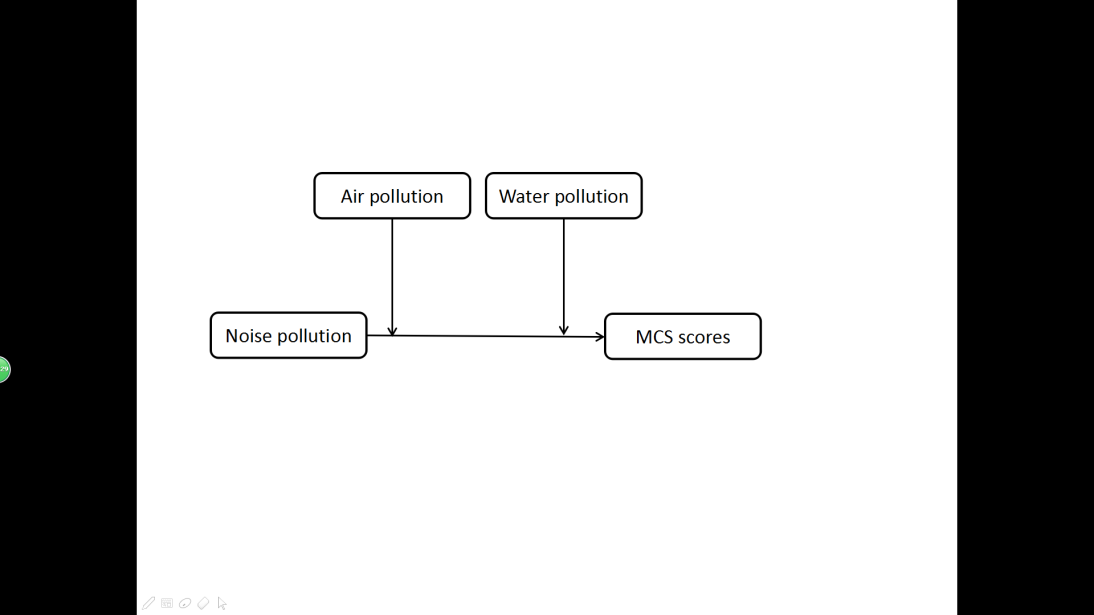


Figure 20. Conceptual diagram

Model = 2 Y = agg_ment X = noise M = air W = water

Statistical Controls:CONTROL= employment bmi hhdnum religion alcohg smokg

Sample size 3697

**************************************************************************

Outcome: agg_ment

Model Summary

R R-sq MSE F df1 df2 p

.14 .02 86.75 6.87 11.00 3685.00 .00

Model

coeff se t p LLCI ULCI

constant 42.66 1.18 36.22 .00 40.35 44.97

air -.50 .44 -1.13 .26 -1.36 .36

noise -.19 .40 -.47 .64 -.97 .59

int_1 1.26 .86 1.47 .14 -.43 2.95

water -.48 .42 -1.14 .26 -1.30 .35

int_2 -.22 .83 -.27 .79 -1.85 1.40

employment -.78 .33 -2.37 .02 -1.42 -.13

bmi .25 .05 5.43 .00 .16 .34

hhdnum -.08 .14 -.54 .59 -.36 .20

religion .48 .47 1.03 .30 -.44 1.40

alcohg .53 .38 1.41 .16 -.21 1.27

smokg 1.23 .36 3.40 .00 .52 1.93

Interactions:

int_1 noise X air

int_2 noise X water

R-square increase due to interaction(s):

R2-chng F df1 df2 p

int_1 .00 2.15 1.00 3685.00 .14

int_2 .00 .07 1.00 3685.00 .79

Both .00 1.16 2.00 3685.00 .31

*************************************************************************

Conditional effect of X on Y at values of the moderator(s):

water air Effect se t p LLCI ULCI

-.27 -.29 -.49 .52 -.96 .34 -1.51 .52

-.27 .71 .76 .78 .98 .33 -.77 2.30

.73 -.29 -.72 .86 -.84 .40 -2.40 .96

.73 .71 .54 .68 .80 .42 -.79 1.87

**************************************************************************

Data for visualizing conditional effect of X on Y

Paste text below into a SPSS syntax window and execute to produce plot.

DATA LIST FREE/noise water air agg_ment.

BEGIN DATA.

-.30 -.27 -.29 49.20

.70 -.27 -.29 48.70

-.30 -.27 .71 48.32

.70 -.27 .71 49.09

-.30 .73 -.29 48.79

.70 .73 -.29 48.07

-.30 .73 .71 47.91

.70 .73 .71 48.45

**Statistical outcomes of potential conceptual diagram 21.**


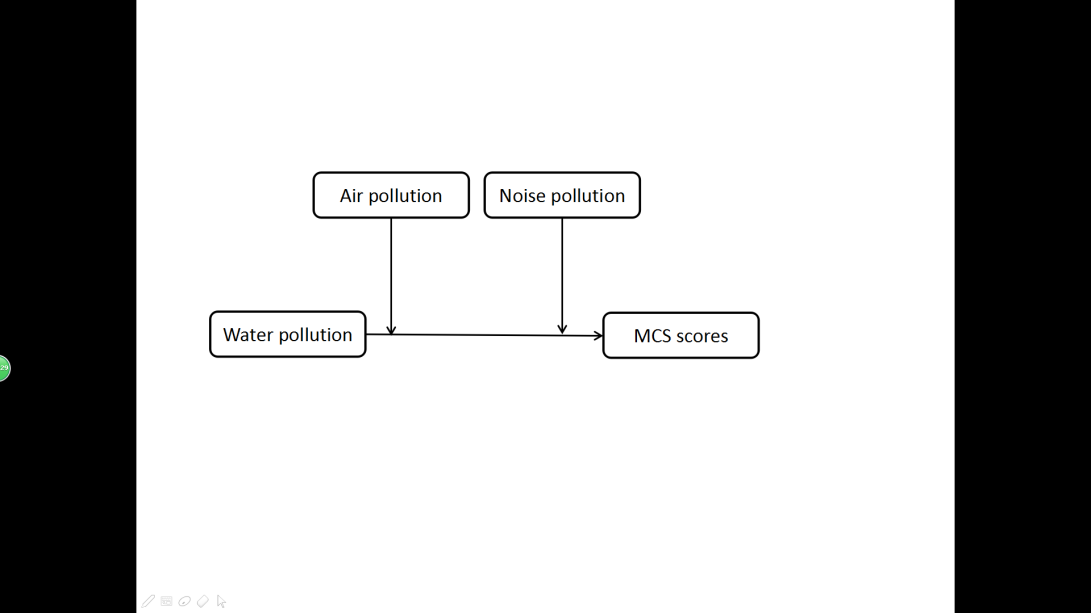


Figure 21. Conceptual diagram

Model = 2 Y = agg_ment X = water M = air W = noise

Statistical Controls:CONTROL= employment bmi hhdnum religion alcohg smokg

Sample size 3697

**************************************************************************

Outcome: agg_ment

Model Summary

R R-sq MSE F df1 df2 p

.14 .02 86.78 6.76 11.00 3685.00 .00

Model

coeff se t p LLCI ULCI

constant 42.60 1.18 36.02 .00 40.28 44.92

air -.43 .44 -.99 .32 -1.29 .43

water -.64 .44 -1.46 .14 -1.49 .22

int_1 .85 .88 .97 .33 -.87 2.58

noise -.06 .39 -.16 .87 -.83 .70

int_2 .00 .81 -.01 1.00 -1.60 1.59

employment -.79 .33 -2.40 .02 -1.43 -.14

bmi .25 .05 5.52 .00 .16 .34

hhdnum -.08 .14 -.54 .59 -.36 .20

religion .48 .47 1.03 .30 -.44 1.40

alcohg .53 .38 1.41 .16 -.21 1.27

smokg 1.22 .36 3.38 .00 .51 1.93

Interactions:

int_1 water X air

int_2 water X noise

R-square increase due to interaction(s):

R2-chng F df1 df2 p

int_1 .00 .94 1.00 3685.00 .33

int_2 .00 .00 1.00 3685.00 1.00

Both .00 .55 2.00 3685.00 .58

*************************************************************************

Conditional effect of X on Y at values of the moderator(s):

noise air Effect se t p LLCI ULCI

-.30 -.29 -.88 .59 -1.50 .13 -2.04 .27

-.30 .71 -.03 .79 -.04 .97 -1.58 1.52

.70 -.29 -.89 .84 -1.05 .29 -2.54 .77

.70 .71 -.04 .66 -.06 .96 -1.32 1.25

**************************************************************************

Data for visualizing conditional effect of X on Y

Paste text below into a SPSS syntax window and execute to produce plot.

DATA LIST FREE/water noise air agg_ment.

BEGIN DATA.

-.27 -.30 -.29 49.18

.73 -.30 -.29 48.29

-.27 -.30 .71 48.52

.73 -.30 .71 48.49

-.27 .70 -.29 49.12

.73 .70 -.29 48.23

-.27 .70 .71 48.46

.73 .70 .71 48.42

**Statistical outcomes of potential conceptual diagram 22.**


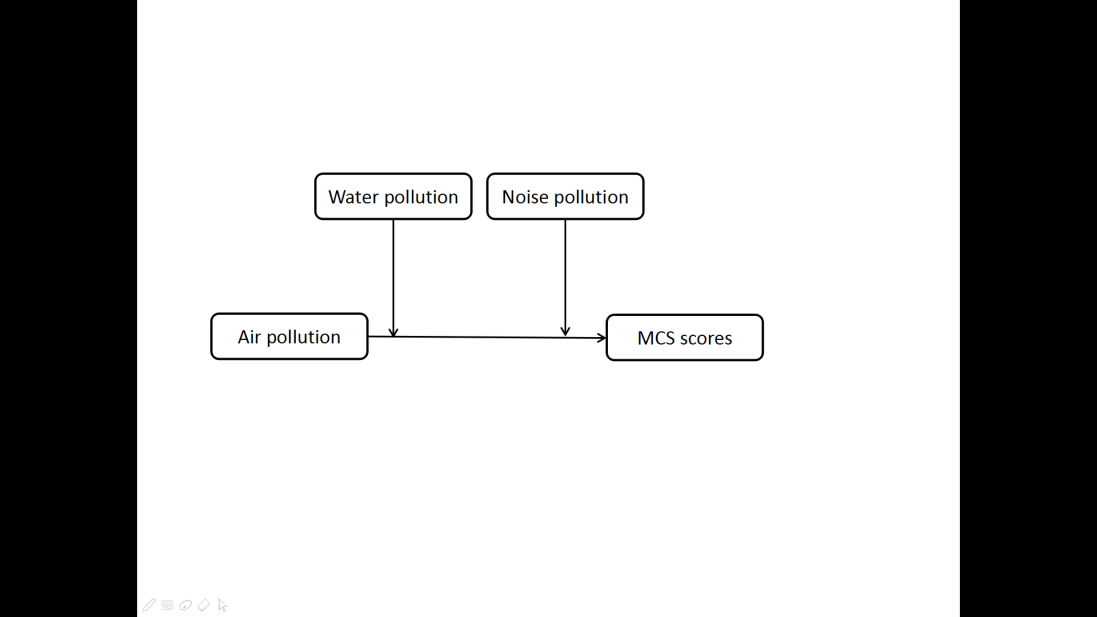


Figure 22. Conceptual diagram

Model = 2 Y = agg_ment X = air M = water W = noise

Statistical Controls:CONTROL= employment bmi hhdnum religion alcohg smokg

Sample size 3697

**************************************************************************

Outcome: agg_ment

Model Summary

R R-sq MSE F df1 df2 p

.14 .02 86.73 6.93 11.00 3685.00 .00

Model

coeff se t p LLCI ULCI

constant 42.55 1.18 35.97 .00 40.23 44.87

water -.63 .43 -1.45 .15 -1.48 .22

air -.56 .45 -1.25 .21 -1.43 .32

int_1 .70 .82 .86 .39 -.90 2.30

noise -.19 .40 -.48 .63 -.97 .59

int_2 1.07 .78 1.37 .17 -.46 2.60

employment -.78 .33 -2.38 .02 -1.42 -.14

bmi .25 .05 5.47 .00 .16 .34

hhdnum -.08 .14 -.54 .59 -.36 .20

religion .48 .47 1.02 .31 -.44 1.40

alcohg .53 .38 1.40 .16 -.21 1.27

smokg 1.23 .36 3.42 .00 .53 1.94

Interactions:

int_1 air X water

int_2 air X noise

R-square increase due to interaction(s):

R2-chng F df1 df2 p

int_1 .00 .73 1.00 3685.00 .39

int_2 .00 1.87 1.00 3685.00 .17

Both .00 1.49 2.00 3685.00 .23

*************************************************************************

Conditional effect of X on Y at values of the moderator(s):

noise water Effect se t p LLCI ULCI

-.30 -.27 -1.06 .60 -1.76 .08 -2.25 .12

-.30 .73 -.36 .77 -.47 .64 -1.88 1.15

.70 -.27 .01 .71 .01 .99 -1.39 1.41

.70 .73 .71 .76 .93 .35 -.78 2.19

**************************************************************************

Data for visualizing conditional effect of X on Y

Paste text below into a SPSS syntax window and execute to produce plot.

DATA LIST FREE/air noise water agg_ment.

BEGIN DATA.

-.29 -.30 -.27 49.24

.71 -.30 -.27 48.18

-.29 -.30 .73 48.41

.71 -.30 .73 48.04

-.29 .70 -.27 48.74

.71 .70 -.27 48.75

-.29 .70 .73 47.91

.71 .70 .73 48.61

**Statistical outcomes of potential conceptual diagram 23.**


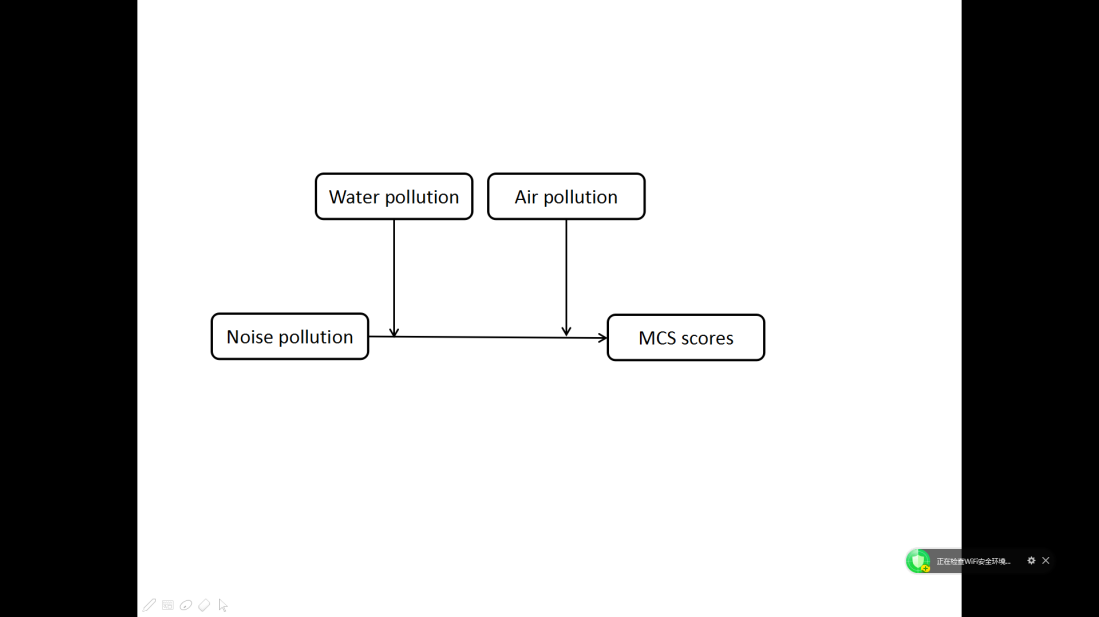


Figure 23. Conceptual diagram

Model = 2 Y = agg_ment X = noise M = water W = air

Statistical Controls:CONTROL= employment bmi hhdnum religion alcohg smokg

Sample size 3697

**************************************************************************

Outcome: agg_ment

Model Summary

R R-sq MSE F df1 df2 p

.14 .02 86.75 6.87 11.00 3685.00 .00

Model

coeff se t p LLCI ULCI

constant 42.66 1.18 36.22 .00 40.35 44.97

water -.48 .42 -1.14 .26 -1.30 .35

noise -.19 .40 -.47 .64 -.97 .59

int_1 -.22 .83 -.27 .79 -1.85 1.40

air -.50 .44 -1.13 .26 -1.36 .36

int_2 1.26 .86 1.47 .14 -.43 2.95

employment -.78 .33 -2.37 .02 -1.42 -.13

bmi .25 .05 5.43 .00 .16 .34

hhdnum -.08 .14 -.54 .59 -.36 .20

religion .48 .47 1.03 .30 -.44 1.40

alcohg .53 .38 1.41 .16 -.21 1.27

smokg 1.23 .36 3.40 .00 .52 1.93

Interactions:

int_1 noise X water

int_2 noise X air

R-square increase due to interaction(s):

R2-chng F df1 df2 p

int_1 .00 .07 1.00 3685.00 .79

int_2 .00 2.15 1.00 3685.00 .14

Both .00 1.16 2.00 3685.00 .31

*************************************************************************

Conditional effect of X on Y at values of the moderator(s):

air water Effect se t p LLCI ULCI

-.29 -.27 -.49 .52 -.96 .34 -1.51 .52

-.29 .73 -.72 .86 -.84 .40 -2.40 .96

.71 -.27 .76 .78 .98 .33 -.77 2.30

.71 .73 .54 .68 .80 .42 -.79 1.87

**************************************************************************

Data for visualizing conditional effect of X on Y

Paste text below into a SPSS syntax window and execute to produce plot.

DATA LIST FREE/noise air water agg_ment.

BEGIN DATA.

-.30 -.29 -.27 49.20

.70 -.29 -.27 48.70

-.30 -.29 .73 48.79

.70 -.29 .73 48.07

-.30 .71 -.27 48.32

.70 .71 -.27 49.09

-.30 .71 .73 47.91

.70 .71 .73 48.45

**Statistical outcomes of potential conceptual diagram 24.**


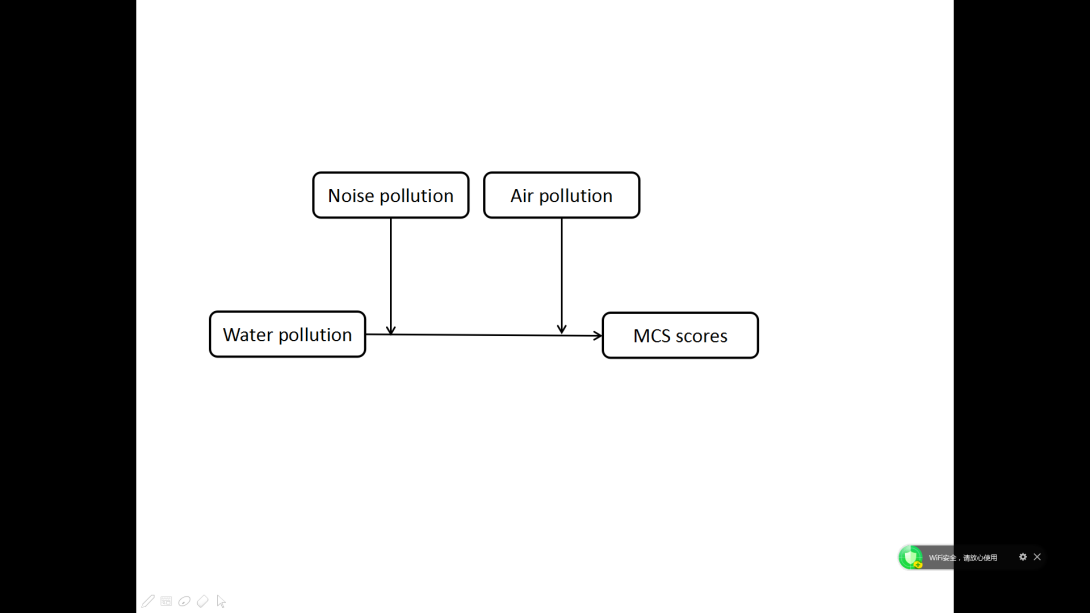


Figure 24. Conceptual diagram

Model = 2 Y = agg_ment X = water M = noise W = air

Statistical Controls:CONTROL= employment bmi hhdnum religion alcohg smokg

Sample size 3697

**************************************************************************

Outcome: agg_ment

Model Summary

R R-sq MSE F df1 df2 p

.14 .02 86.78 6.76 11.00 3685.00 .00

Model

coeff se t p LLCI ULCI

constant 42.60 1.18 36.02 .00 40.28 44.92

noise -.06 .39 -.16 .87 -.83 .70

water -.64 .44 -1.46 .14 -1.49 .22

int_1 .00 .81 -.01 1.00 -1.60 1.59

air -.43 .44 -.99 .32 -1.29 .43

int_2 .85 .88 .97 .33 -.87 2.58

employment -.79 .33 -2.40 .02 -1.43 -.14

bmi .25 .05 5.52 .00 .16 .34

hhdnum -.08 .14 -.54 .59 -.36 .20

religion .48 .47 1.03 .30 -.44 1.40

alcohg .53 .38 1.41 .16 -.21 1.27

smokg 1.22 .36 3.38 .00 .51 1.93

Interactions:

int_1 water X noise

int_2 water X air

R-square increase due to interaction(s):

R2-chng F df1 df2 p

int_1 .00 .00 1.00 3685.00 1.00

int_2 .00 .94 1.00 3685.00 .33

Both .00 .55 2.00 3685.00 .58

*************************************************************************

Conditional effect of X on Y at values of the moderator(s):

air noise Effect se t p LLCI ULCI

-.29 -.30 -.88 .59 -1.50 .13 -2.04 .27

-.29 .70 -.89 .84 -1.05 .29 -2.54 .77

.71 -.30 -.03 .79 -.04 .97 -1.58 1.52

.71 .70 -.04 .66 -.06 .96 -1.32 1.25

**************************************************************************

Data for visualizing conditional effect of X on Y

Paste text below into a SPSS syntax window and execute to produce plot.

DATA LIST FREE/water air noise agg_ment.

BEGIN DATA.

-.27 -.29 -.30 49.18

.73 -.29 -.30 48.29

-.27 -.29 .70 49.12

.73 -.29 .70 48.23

-.27 .71 -.30 48.52

.73 .71 -.30 48.49

-.27 .71 .70 48.46

.73 .71 .70 48.42

**Statistical outcomes of potential conceptual diagram 25.**


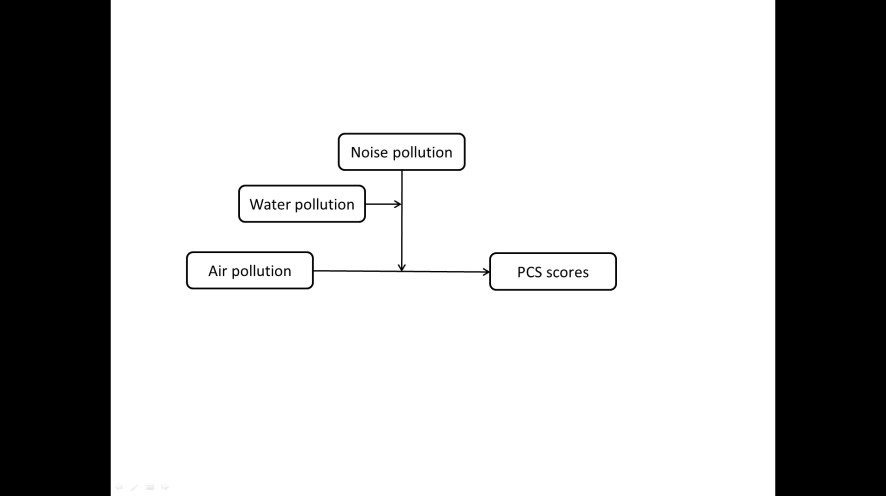


Figure 25. Conceptual diagram

Model = 3 Y = agg_phys X = air M = noise W = water

Statistical Controls:CONTROL= employment bmi hhdnum religion alcohg smokg

Sample size 3697

**************************************************************************

Outcome: agg_phys

Model Summary

R R-sq MSE F df1 df2 p

.32 .10 107.94 29.84 13.00 3683.00 .00

Model

coeff se t p LLCI ULCI

constant 43.35 1.41 30.83 .00 40.59 46.11

noise 1.09 .47 2.31 .02 .16 2.01

air .68 .50 1.36 .17 -.30 1.66

int_1 -1.56 .96 -1.63 .10 -3.44 .32

water -1.42 .54 -2.64 .01 -2.47 -.36

int_2 1.69 1.02 1.66 .10 -.31 3.69

int_3 .21 1.17 .18 .86 -2.09 2.51

int_4 .92 2.03 .45 .65 -3.05 4.90

employment -5.38 .39 -13.66 .00 -6.16 -4.61

bmi .17 .05 3.07 .00 .06 .27

hhdnum .77 .17 4.60 .00 .44 1.09

religion 1.41 .54 2.62 .01 .36 2.47

alcohg -.69 .41 -1.68 .09 -1.49 .11

smokg 3.42 .39 8.81 .00 2.66 4.18

Interactions:

int_1 air X noise

int_2 air X water

int_3 noise X water

int_4 air X noise X water

*************************************************************************

Conditional effect of X on Y at values of the moderator(s):

water noise Effect se t p LLCI ULCI

-.27 -.30 .77 .80 .97 .33 -.79 2.34

-.27 .70 -1.03 .84 -1.22 .22 -2.68 .62

.73 -.30 2.19 1.03 2.13 .03 .18 4.21

.73 .70 1.31 1.31 1.00 .32 -1.26 3.87

Conditional effect of X*M interaction at values of W:

water Effect se t p LLCI ULCI

-.27 -1.81 1.16 -1.56 .12 -4.08 .47

.73 -.89 1.66 -.53 .59 -4.15 2.38

**************************************************************************

Data for visualizing conditional effect of X on Y

Paste text below into a SPSS syntax window and execute to produce plot.

DATA LIST FREE/air water noise agg_phys.

BEGIN DATA.

-.29 -.27 -.30 49.25

.71 -.27 -.30 50.03

-.29 -.27 .70 50.81

.71 -.27 .70 49.77

-.29 .73 -.30 47.36

.71 .73 -.30 49.55

-.29 .73 .70 48.86

.71 .73 .70 50.16

**Statistical outcomes of potential conceptual diagram 26.**


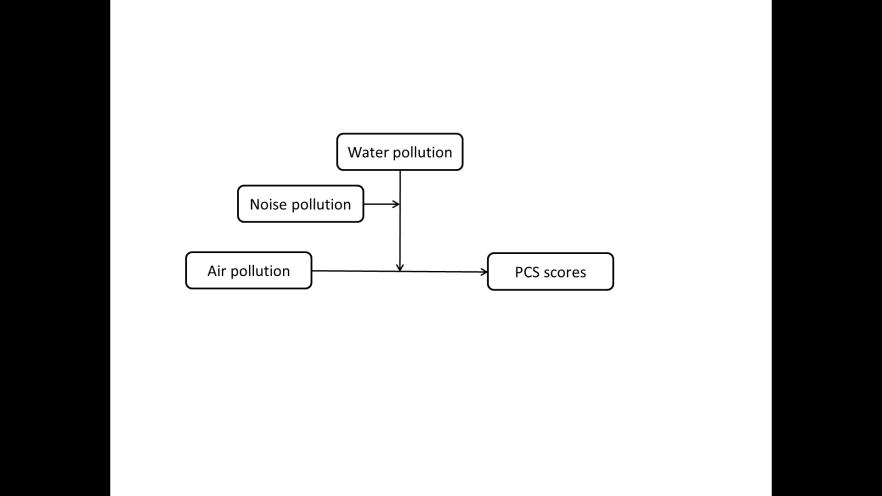


Figure 26. Conceptual diagram

Model = 3 Y = agg_phys X = air M = water W = noise

Statistical Controls:CONTROL= employment bmi hhdnum religion alcohg smokg

Sample size 3697

**************************************************************************

Outcome: agg_phys

Model Summary

R R-sq MSE F df1 df2 p

.32 .10 107.94 29.84 13.00 3683.00 .00

Model

coeff se t p LLCI ULCI

constant 43.35 1.41 30.83 .00 40.59 46.11

water -1.42 .54 -2.64 .01 -2.47 -.36

air .68 .50 1.36 .17 -.30 1.66

int_1 1.69 1.02 1.66 .10 -.31 3.69

noise 1.09 .47 2.31 .02 .16 2.01

int_2 -1.56 .96 -1.63 .10 -3.44 .32

int_3 .21 1.17 .18 .86 -2.09 2.51

int_4 .92 2.03 .45 .65 -3.05 4.90

employment -5.38 .39 -13.66 .00 -6.16 -4.61

bmi .17 .05 3.07 .00 .06 .27

hhdnum .77 .17 4.60 .00 .44 1.09

religion 1.41 .54 2.62 .01 .36 2.47

alcohg -.69 .41 -1.68 .09 -1.49 .11

smokg 3.42 .39 8.81 .00 2.66 4.18

Interactions:

int_1 air X water

int_2 air X noise

int_3 water X noise

int_4 air X water X noise

*************************************************************************

Conditional effect of X on Y at values of the moderator(s):

noise water Effect se t p LLCI ULCI

-.30 -.27 .77 .80 .97 .33 -.79 2.34

-.30 .73 2.19 1.03 2.13 .03 .18 4.21

.70 -.27 -1.03 .84 -1.22 .22 -2.68 .62

.70 .73 1.31 1.31 1.00 .32 -1.26 3.87

Conditional effect of X*M interaction at values of W:

noise Effect se t p LLCI ULCI

-.30 1.42 1.30 1.09 .28 -1.13 3.96

.70 2.34 1.55 1.50 .13 -.71 5.38

**************************************************************************

Data for visualizing conditional effect of X on Y

Paste text below into a SPSS syntax window and execute to produce plot.

DATA LIST FREE/air noise water agg_phys.

BEGIN DATA.

-.29 -.30 -.27 49.25

.71 -.30 -.27 50.03

-.29 -.30 .73 47.36

.71 -.30 .73 49.55

-.29 .70 -.27 50.81

.71 .70 -.27 49.77

-.29 .70 .73 48.86

.71 .70 .73 50.16

**Statistical outcomes of potential conceptual diagram 27.**


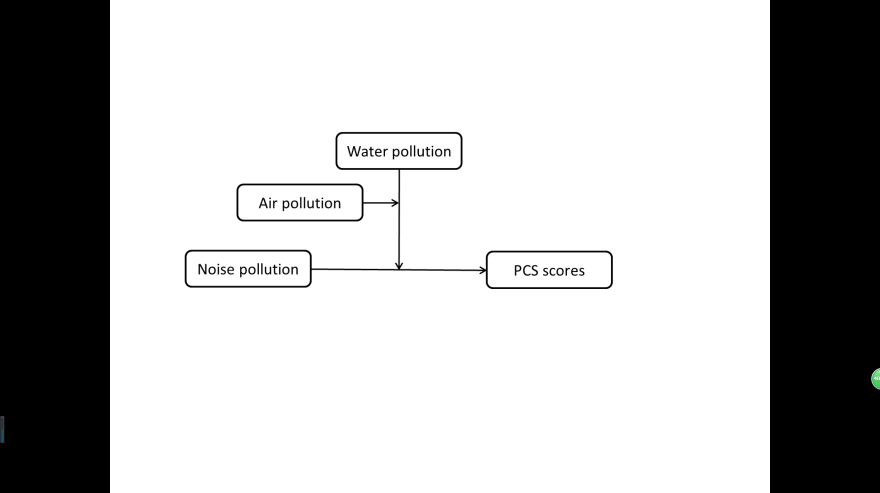


Figure 27. Conceptual diagram

Model = 3 Y = agg_phys X = noise M = water W = air

Statistical Controls:CONTROL= employment bmi hhdnum religion alcohg smokg

Sample size 3697

**************************************************************************

Outcome: agg_phys

Model Summary

R R-sq MSE F df1 df2 p

.32 .10 107.94 29.84 13.00 3683.00 .00

Model

coeff se t p LLCI ULCI

constant 43.35 1.41 30.83 .00 40.59 46.11

water -1.42 .54 -2.64 .01 -2.47 -.36

noise 1.09 .47 2.31 .02 .16 2.01

int_1 .21 1.17 .18 .86 -2.09 2.51

air .68 .50 1.36 .17 -.30 1.66

int_2 -1.56 .96 -1.63 .10 -3.44 .32

int_3 1.69 1.02 1.66 .10 -.31 3.69

int_4 .92 2.03 .45 .65 -3.05 4.90

employment -5.38 .39 -13.66 .00 -6.16 -4.61

bmi .17 .05 3.07 .00 .06 .27

hhdnum .77 .17 4.60 .00 .44 1.09

religion 1.41 .54 2.62 .01 .36 2.47

alcohg -.69 .41 -1.68 .09 -1.49 .11

smokg 3.42 .39 8.81 .00 2.66 4.18

Interactions:

int_1 noise X water

int_2 noise X air

int_3 water X air

int_4 noise X water X air

*************************************************************************

Conditional effect of X on Y at values of the moderator(s):

air water Effect se t p LLCI ULCI

-.29 -.27 1.55 .60 2.59 .01 .38 2.73

-.29 .73 1.50 1.44 1.04 .30 -1.33 4.33

.71 -.27 -.25 .99 -.25 .80 -2.20 1.70

.71 .73 .61 .83 .74 .46 -1.01 2.23

Conditional effect of X*M interaction at values of W:

air Effect se t p LLCI ULCI

-.29 -.06 1.56 -.04 .97 -3.12 3.01

.71 .86 1.29 .67 .50 -1.67 3.40

**************************************************************************

Data for visualizing conditional effect of X on Y

Paste text below into a SPSS syntax window and execute to produce plot.

DATA LIST FREE/noise air water agg_phys.

BEGIN DATA.

-.30 -.29 -.27 49.25

.70 -.29 -.27 50.81

-.30 -.29 .73 47.36

.70 -.29 .73 48.86

-.30 .71 -.27 50.03

.70 .71 -.27 49.77

-.30 .71 .73 49.55

.70 .71 .73 50.16

END DATA.

GRAPH/SCATTERPLOT=noise WITH agg_phys BY water/PANEL ROWVAR=air.

* Estimates are based on setting covariates to their sample means.

**Statistical outcomes of potential conceptual diagram 28.**


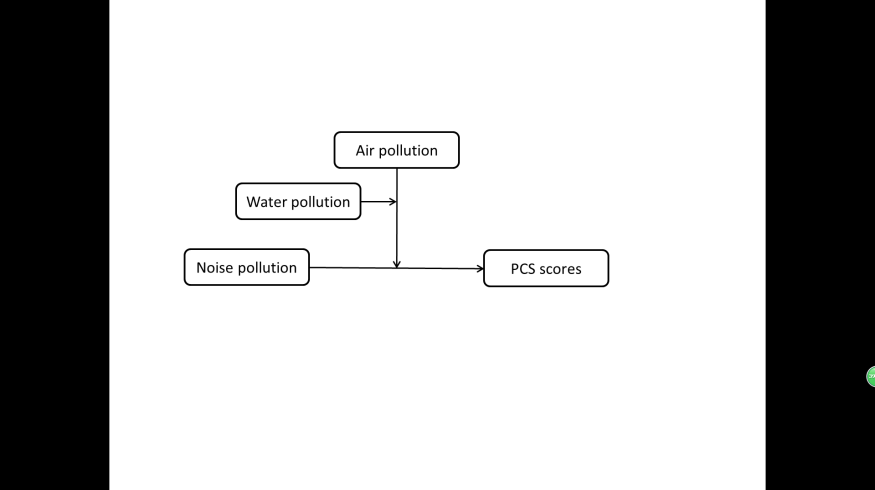


Figure 28. Conceptual diagram

Model = 3 Y = agg_phys X = noise M = air W = water

Statistical Controls:

CONTROL= employment bmi hhdnum religion alcohg smokg

Sample size 3697

**************************************************************************

Outcome: agg_phys

Model Summary

R R-sq MSE F df1 df2 p

.32 .10 107.94 29.84 13.00 3683.00 .00

Model

coeff se t p LLCI ULCI

constant 43.35 1.41 30.83 .00 40.59 46.11

air .68 .50 1.36 .17 -.30 1.66

noise 1.09 .47 2.31 .02 .16 2.01

int_1 -1.56 .96 -1.63 .10 -3.44 .32

water -1.42 .54 -2.64 .01 -2.47 -.36

int_2 .21 1.17 .18 .86 -2.09 2.51

int_3 1.69 1.02 1.66 .10 -.31 3.69

int_4 .92 2.03 .45 .65 -3.05 4.90

employment -5.38 .39 -13.66 .00 -6.16 -4.61

bmi .17 .05 3.07 .00 .06 .27

hhdnum .77 .17 4.60 .00 .44 1.09

religion 1.41 .54 2.62 .01 .36 2.47

alcohg -.69 .41 -1.68 .09 -1.49 .11

smokg 3.42 .39 8.81 .00 2.66 4.18

Interactions:

int_1 noise X air

int_2 noise X water

int_3 air X water

int_4 noise X air X water

*************************************************************************

Conditional effect of X on Y at values of the moderator(s):

water air Effect se t p LLCI ULCI

-.27 -.29 1.55 .60 2.59 .01 .38 2.73

-.27 .71 -.25 .99 -.25 .80 -2.20 1.70

.73 -.29 1.50 1.44 1.04 .30 -1.33 4.33

.73 .71 .61 .83 .74 .46 -1.01 2.23

Conditional effect of X*M interaction at values of W:

water Effect se t p LLCI ULCI

-.27 -1.81 1.16 -1.56 .12 -4.08 .47

.73 -.89 1.66 -.53 .59 -4.15 2.38

**************************************************************************

Data for visualizing conditional effect of X on Y

Paste text below into a SPSS syntax window and execute to produce plot.

DATA LIST FREE/noise water air agg_phys.

BEGIN DATA.

-.30 -.27 -.29 49.25

.70 -.27 -.29 50.81

-.30 -.27 .71 50.03

.70 -.27 .71 49.77

-.30 .73 -.29 47.36

.70 .73 -.29 48.86

-.30 .73 .71 49.55

.70 .73 .71 50.16

**Statistical outcomes of potential conceptual diagram 29.**


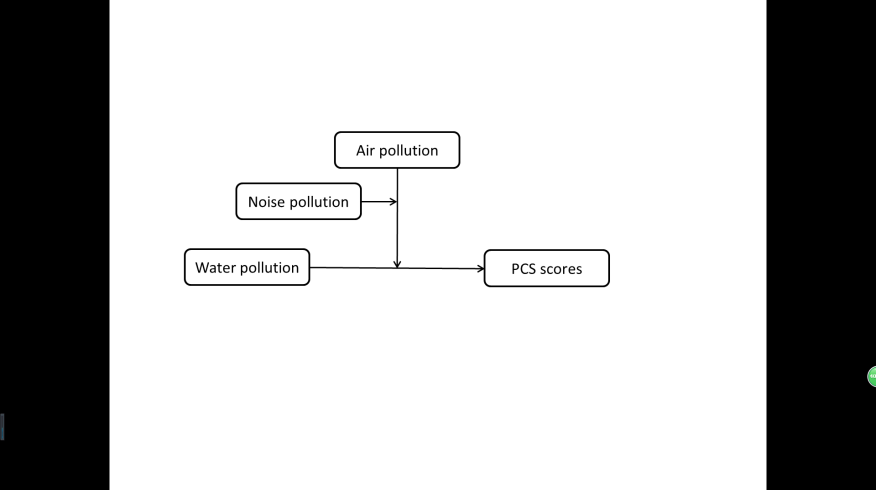


Figure 29. Conceptual diagram

Model = 3 Y = agg_phys X = water M = air W = noise

Statistical Controls:CONTROL= employment bmi hhdnum religion alcohg smokg

Sample size 3697

**************************************************************************

Outcome: agg_phys

Model Summary

R R-sq MSE F df1 df2 p

.32 .10 107.94 29.84 13.00 3683.00 .00

Model

coeff se t p LLCI ULCI

constant 43.35 1.41 30.83 .00 40.59 46.11

air .68 .50 1.36 .17 -.30 1.66

water -1.42 .54 -2.64 .01 -2.47 -.36

int_1 1.69 1.02 1.66 .10 -.31 3.69

noise 1.09 .47 2.31 .02 .16 2.01

int_2 .21 1.17 .18 .86 -2.09 2.51

int_3 -1.56 .96 -1.63 .10 -3.44 .32

int_4 .92 2.03 .45 .65 -3.05 4.90

employment -5.38 .39 -13.66 .00 -6.16 -4.61

bmi .17 .05 3.07 .00 .06 .27

hhdnum .77 .17 4.60 .00 .44 1.09

religion 1.41 .54 2.62 .01 .36 2.47

alcohg -.69 .41 -1.68 .09 -1.49 .11

smokg 3.42 .39 8.81 .00 2.66 4.18

Interactions:

int_1 water X air

int_2 water X noise

int_3 air X noise

int_4 water X air X noise

*************************************************************************

Conditional effect of X on Y at values of the moderator(s):

noise air Effect se t p LLCI ULCI

-.30 -.29 -1.89 .80 -2.36 .02 -3.46 -.32

-.30 .71 -.48 1.02 -.47 .64 -2.48 1.53

.70 -.29 -1.95 1.34 -1.45 .15 -4.58 .68

.70 .71 .39 .79 .49 .62 -1.16 1.93

Conditional effect of X*M interaction at values of W:

noise Effect se t p LLCI ULCI

-.30 1.42 1.30 1.09 .28 -1.13 3.96

.70 2.34 1.55 1.50 .13 -.71 5.38

**************************************************************************

Data for visualizing conditional effect of X on Y

Paste text below into a SPSS syntax window and execute to produce plot.

DATA LIST FREE/water noise air agg_phys.

BEGIN DATA.

-.27 -.30 -.29 49.25

.73 -.30 -.29 47.36

-.27 -.30 .71 50.03

.73 -.30 .71 49.55

-.27 .70 -.29 50.81

.73 .70 -.29 48.86

-.27 .70 .71 49.77

.73 .70 .71 50.16

**Statistical outcomes of potential conceptual diagram 30.**


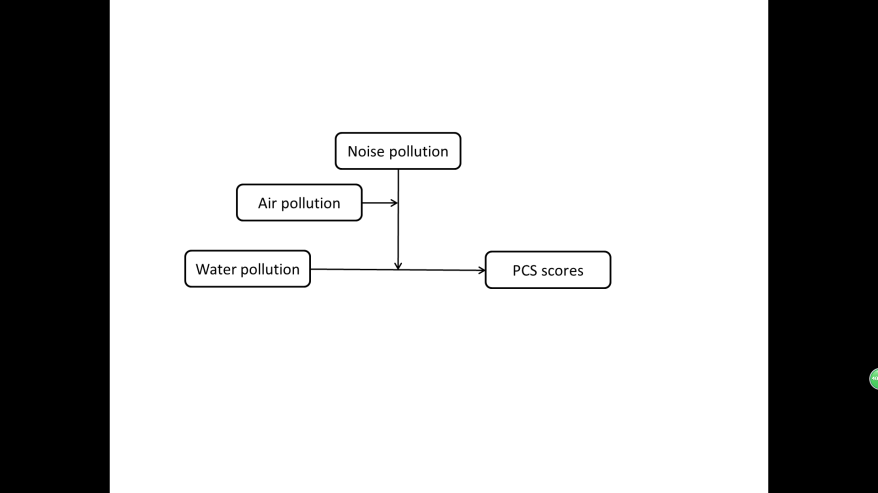


Figure 30. Conceptual diagram

Model = 3 Y = agg_phys X = water M = noise W = air

Statistical Controls:CONTROL= employment bmi hhdnum religion alcohg smokg

Sample size 3697

**************************************************************************

Outcome: agg_phys

Model Summary

R R-sq MSE F df1 df2 p

.32 .10 107.94 29.84 13.00 3683.00 .00

Model

coeff se t p LLCI ULCI

constant 43.35 1.41 30.83 .00 40.59 46.11

noise 1.09 .47 2.31 .02 .16 2.01

water -1.42 .54 -2.64 .01 -2.47 -.36

int_1 .21 1.17 .18 .86 -2.09 2.51

air .68 .50 1.36 .17 -.30 1.66

int_2 1.69 1.02 1.66 .10 -.31 3.69

int_3 -1.56 .96 -1.63 .10 -3.44 .32

int_4 .92 2.03 .45 .65 -3.05 4.90

employment -5.38 .39 -13.66 .00 -6.16 -4.61

bmi .17 .05 3.07 .00 .06 .27

hhdnum .77 .17 4.60 .00 .44 1.09

religion 1.41 .54 2.62 .01 .36 2.47

alcohg -.69 .41 -1.68 .09 -1.49 .11

smokg 3.42 .39 8.81 .00 2.66 4.18

Interactions:

int_1 water X noise

int_2 water X air

int_3 noise X air

int_4 water X noise X air

*************************************************************************

Conditional effect of X on Y at values of the moderator(s):

air noise Effect se t p LLCI ULCI

-.29 -.30 -1.89 .80 -2.36 .02 -3.46 -.32

-.29 .70 -1.95 1.34 -1.45 .15 -4.58 .68

.71 -.30 -.48 1.02 -.47 .64 -2.48 1.53

.71 .70 .39 .79 .49 .62 -1.16 1.93

Conditional effect of X*M interaction at values of W:

air Effect se t p LLCI ULCI

-.29 -.06 1.56 -.04 .97 -3.12 3.01

.71 .86 1.29 .67 .50 -1.67 3.40

**************************************************************************

Data for visualizing conditional effect of X on Y

Paste text below into a SPSS syntax window and execute to produce plot.

DATA LIST FREE/water air noise agg_phys.

BEGIN DATA.

-.27 -.29 -.30 49.25

.73 -.29 -.30 47.36

-.27 -.29 .70 50.81

.73 -.29 .70 48.86

-.27 .71 -.30 50.03

.73 .71 -.30 49.55

-.27 .71 .70 49.77

.73 .71 .70 50.16

**Statistical outcomes of potential conceptual diagram 31.**


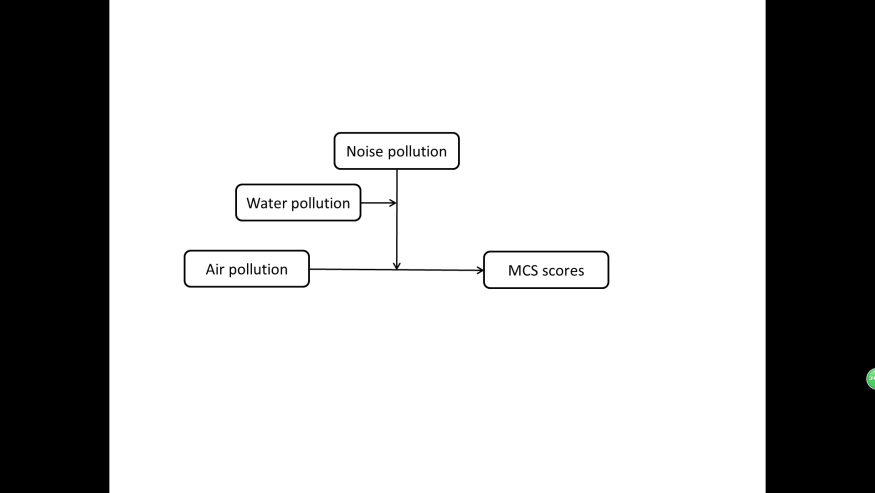


Figure 31. Conceptual diagram

Model = 3 Y = agg_ment X = air M = noise W = water

Statistical Controls:CONTROL= employment bmi hhdnum religion alcohg smokg

Sample size 3697

**************************************************************************

Outcome: agg_ment

Model Summary

R R-sq MSE F df1 df2 p

.15 .02 86.64 6.16 13.00 3683.00 .00

Model

coeff se t p LLCI ULCI

constant 42.59 1.21 35.16 .00 40.21 44.96

noise -.50 .42 -1.17 .24 -1.33 .34

air -.51 .44 -1.14 .25 -1.38 .36

int_1 .89 .87 1.02 .31 -.82 2.60

water -.87 .46 -1.88 .06 -1.78 .04

int_2 .43 .91 .48 .63 -1.35 2.21

int_3 -1.61 1.07 -1.51 .13 -3.70 .48

int_4 4.22 1.85 2.28 .02 .59 7.86

employment -.78 .34 -2.30 .02 -1.44 -.11

bmi .25 .05 5.33 .00 .16 .34

hhdnum -.07 .14 -.52 .60 -.35 .20

religion .51 .47 1.07 .29 -.42 1.44

alcohg .53 .37 1.44 .15 -.19 1.25

smokg 1.22 .35 3.46 .00 .53 1.92

Interactions:

int_1 air X noise

int_2 air X water

int_3 noise X water

int_4 air X noise X water

*************************************************************************

Conditional effect of X on Y at values of the moderator(s):

water noise Effect se t p LLCI ULCI

-.27 -.30 -.55 .70 -.80 .43 -1.92 .81

-.27 .70 -.78 .79 -1.00 .32 -2.33 .76

.73 -.30 -1.38 .90 -1.54 .12 -3.15 .38

.73 .70 2.60 1.24 2.10 .04 .18 5.03

Conditional effect of X*M interaction at values of W:

water Effect se t p LLCI ULCI

-.27 -.23 1.05 -.22 .82 -2.29 1.83

.73 3.99 1.53 2.61 .01 .99 6.99

**************************************************************************

Data for visualizing conditional effect of X on Y

Paste text below into a SPSS syntax window and execute to produce plot.

DATA LIST FREE/air water noise agg_ment.

BEGIN DATA.

-.29 -.27 -.30 49.17

.71 -.27 -.30 48.61

-.29 -.27 .70 49.17

.71 -.27 .70 48.38

-.29 .73 -.30 49.02

.71 .73 -.30 47.64

-.29 .73 .70 46.18

.71 .73 .70 48.79

END DATA.

GRAPH/SCATTERPLOT=air WITH agg_ment BY noise/PANEL ROWVAR=water.

* Estimates are based on setting covariates to their sample means.

******************** ANALYSIS NOTES AND WARNINGS *************************

**Statistical outcomes of potential conceptual diagram 32.**


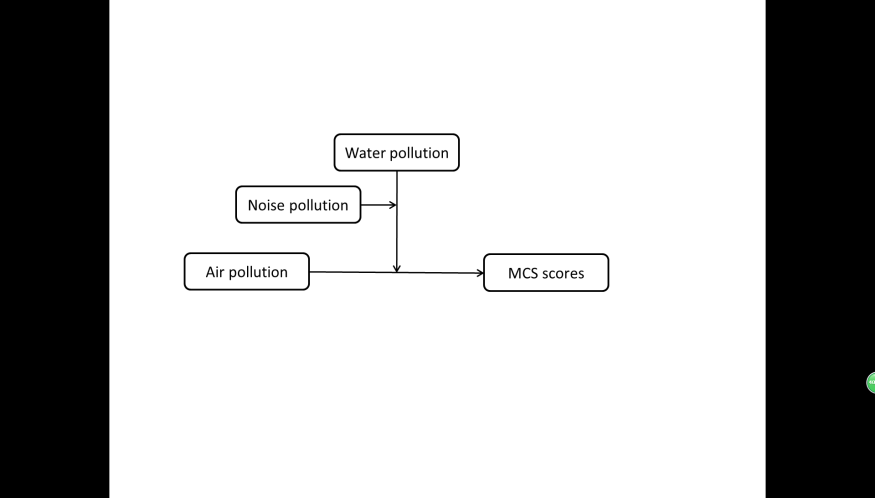


Figure 32. Conceptual diagram

Model = 3 Y = agg_ment X = air M = water W = noise

Statistical Controls:CONTROL= employment bmi hhdnum religion alcohg smokg

Sample size 3697

**************************************************************************

Outcome: agg_ment

Model Summary

R R-sq MSE F df1 df2 p

.15 .02 86.64 6.16 13.00 3683.00 .00

Model

coeff se t p LLCI ULCI

constant 42.59 1.21 35.16 .00 40.21 44.96

water -.87 .46 -1.88 .06 -1.78 .04

air -.51 .44 -1.14 .25 -1.38 .36

int_1 .43 .91 .48 .63 -1.35 2.21

noise -.50 .42 -1.17 .24 -1.33 .34

int_2 .89 .87 1.02 .31 -.82 2.60

int_3 -1.61 1.07 -1.51 .13 -3.70 .48

int_4 4.22 1.85 2.28 .02 .59 7.86

employment -.78 .34 -2.30 .02 -1.44 -.11

bmi .25 .05 5.33 .00 .16 .34

hhdnum -.07 .14 -.52 .60 -.35 .20

religion .51 .47 1.07 .29 -.42 1.44

alcohg .53 .37 1.44 .15 -.19 1.25

smokg 1.22 .35 3.46 .00 .53 1.92

Interactions:

int_1 air X water

int_2 air X noise

int_3 water X noise

int_4 air X water X noise

*************************************************************************

Conditional effect of X on Y at values of the moderator(s):

noise water Effect se t p LLCI ULCI

-.30 -.27 -.55 .70 -.80 .43 -1.92 .81

-.30 .73 -1.38 .90 -1.54 .12 -3.15 .38

.70 -.27 -.78 .79 -1.00 .32 -2.33 .76

.70 .73 2.60 1.24 2.10 .04 .18 5.03

Conditional effect of X*M interaction at values of W:

noise Effect se t p LLCI ULCI

-.30 -.83 1.13 -.73 .46 -3.06 1.39

.70 3.39 1.47 2.31 .02 .51 6.27

**************************************************************************

Data for visualizing conditional effect of X on Y

Paste text below into a SPSS syntax window and execute to produce plot.

DATA LIST FREE/air noise water agg_ment.

BEGIN DATA.

-.29 -.30 -.27 49.17

.71 -.30 -.27 48.61

-.29 -.30 .73 49.02

.71 -.30 .73 47.64

-.29 .70 -.27 49.17

.71 .70 -.27 48.38

-.29 .70 .73 46.18

.71 .70 .73 48.79

END DATA.

GRAPH/SCATTERPLOT=air WITH agg_ment BY water/PANEL ROWVAR=noise.

* Estimates are based on setting covariates to their sample means.

******************** ANALYSIS NOTES AND WARNINGS *************************

**Statistical outcomes of potential conceptual diagram 33.**


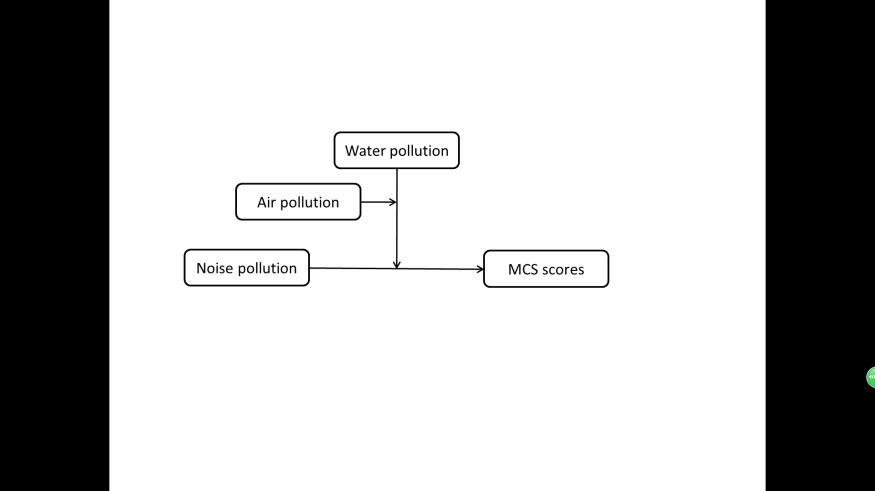


Figure 33. Conceptual diagram

Model = 3 Y = agg_ment X = noise M = water W = air

Statistical Controls:CONTROL= employment bmi hhdnum religion alcohg smokg

Sample size 3697

**************************************************************************

Outcome: agg_ment

Model Summary

R R-sq MSE F df1 df2 p

.15 .02 86.64 6.16 13.00 3683.00 .00

Model

coeff se t p LLCI ULCI

constant 42.59 1.21 35.16 .00 40.21 44.96

water -.87 .46 -1.88 .06 -1.78 .04

noise -.50 .42 -1.17 .24 -1.33 .34

int_1 -1.61 1.07 -1.51 .13 -3.70 .48

air -.51 .44 -1.14 .25 -1.38 .36

int_2 .89 .87 1.02 .31 -.82 2.60

int_3 .43 .91 .48 .63 -1.35 2.21

int_4 4.22 1.85 2.28 .02 .59 7.86

employment -.78 .34 -2.30 .02 -1.44 -.11

bmi .25 .05 5.33 .00 .16 .34

hhdnum -.07 .14 -.52 .60 -.35 .20

religion .51 .47 1.07 .29 -.42 1.44

alcohg .53 .37 1.44 .15 -.19 1.25

smokg 1.22 .35 3.46 .00 .53 1.92

Interactions:

int_1 noise X water

int_2 noise X air

int_3 water X air

int_4 noise X water X air

*************************************************************************

Conditional effect of X on Y at values of the moderator(s):

air water Effect se t p LLCI ULCI

-.29 -.27 .00 .54 .00 1.00 -1.05 1.05

-.29 .73 -2.84 1.31 -2.16 .03 -5.41 -.27

.71 -.27 -.23 .90 -.26 .80 -2.00 1.54

.71 .73 1.15 .79 1.46 .14 -.39 2.70

Conditional effect of X*M interaction at values of W:

air Effect se t p LLCI ULCI

-.29 -2.84 1.42 -2.00 .05 -5.62 -.06

.71 1.38 1.20 1.16 .25 -.96 3.73

**************************************************************************

Data for visualizing conditional effect of X on Y

Paste text below into a SPSS syntax window and execute to produce plot.

DATA LIST FREE/noise air water agg_ment.

BEGIN DATA.

-.30 -.29 -.27 49.17

.70 -.29 -.27 49.17

-.30 -.29 .73 49.02

.70 -.29 .73 46.18

-.30 .71 -.27 48.61

.70 .71 -.27 48.38

-.30 .71 .73 47.64

.70 .71 .73 48.79

END DATA.

GRAPH/SCATTERPLOT=noise WITH agg_ment BY water/PANEL ROWVAR=air.

* Estimates are based on setting covariates to their sample means.

**Statistical outcomes of potential conceptual diagram 34.**


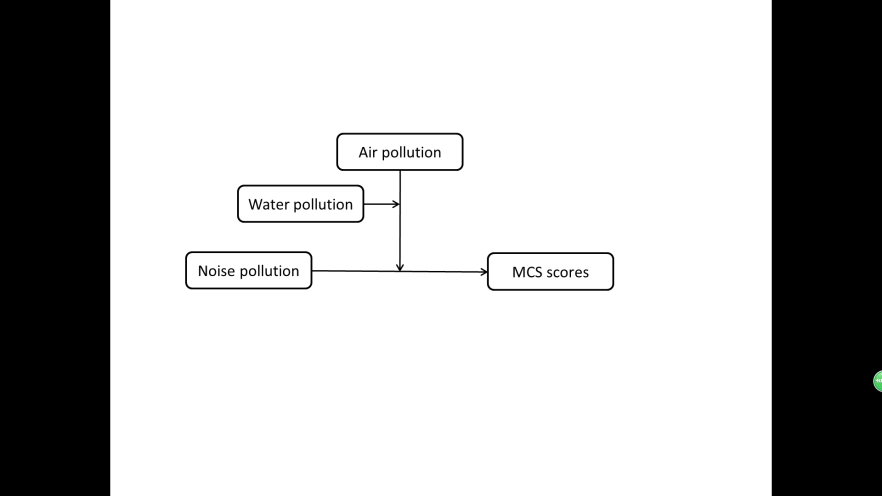


Figure 34. Conceptual diagram

Model = 3 Y = agg_ment X = noise M = air W = water

Statistical Controls:CONTROL= employment bmi hhdnum religion alcohg smokg

Sample size 3697

**************************************************************************

Outcome: agg_ment

Model Summary

R R-sq MSE F df1 df2 p

.15 .02 86.64 6.16 13.00 3683.00 .00

Model

coeff se t p LLCI ULCI

constant 42.59 1.21 35.16 .00 40.21 44.96

air -.51 .44 -1.14 .25 -1.38 .36

noise -.50 .42 -1.17 .24 -1.33 .34

int_1 .89 .87 1.02 .31 -.82 2.60

water -.87 .46 -1.88 .06 -1.78 .04

int_2 -1.61 1.07 -1.51 .13 -3.70 .48

int_3 .43 .91 .48 .63 -1.35 2.21

int_4 4.22 1.85 2.28 .02 .59 7.86

employment -.78 .34 -2.30 .02 -1.44 -.11

bmi .25 .05 5.33 .00 .16 .34

hhdnum -.07 .14 -.52 .60 -.35 .20

religion .51 .47 1.07 .29 -.42 1.44

alcohg .53 .37 1.44 .15 -.19 1.25

smokg 1.22 .35 3.46 .00 .53 1.92

Interactions:

int_1 noise X air

int_2 noise X water

int_3 air X water

int_4 noise X air X water

*************************************************************************

Conditional effect of X on Y at values of the moderator(s):

water air Effect se t p LLCI ULCI

-.27 -.29 .00 .54 .00 1.00 -1.05 1.05

-.27 .71 -.23 .90 -.26 .80 -2.00 1.54

.73 -.29 -2.84 1.31 -2.16 .03 -5.41 -.27

.73 .71 1.15 .79 1.46 .14 -.39 2.70

Conditional effect of X*M interaction at values of W:

water Effect se t p LLCI ULCI

-.27 -.23 1.05 -.22 .82 -2.29 1.83

.73 3.99 1.53 2.61 .01 .99 6.99

**************************************************************************

Data for visualizing conditional effect of X on Y

Paste text below into a SPSS syntax window and execute to produce plot.

DATA LIST FREE/noise water air agg_ment.

BEGIN DATA.

-.30 -.27 -.29 49.17

.70 -.27 -.29 49.17

-.30 -.27 .71 48.61

.70 -.27 .71 48.38

-.30 .73 -.29 49.02

.70 .73 -.29 46.18

-.30 .73 .71 47.64

.70 .73 .71 48.79

**Statistical outcomes of potential conceptual diagram 35.**


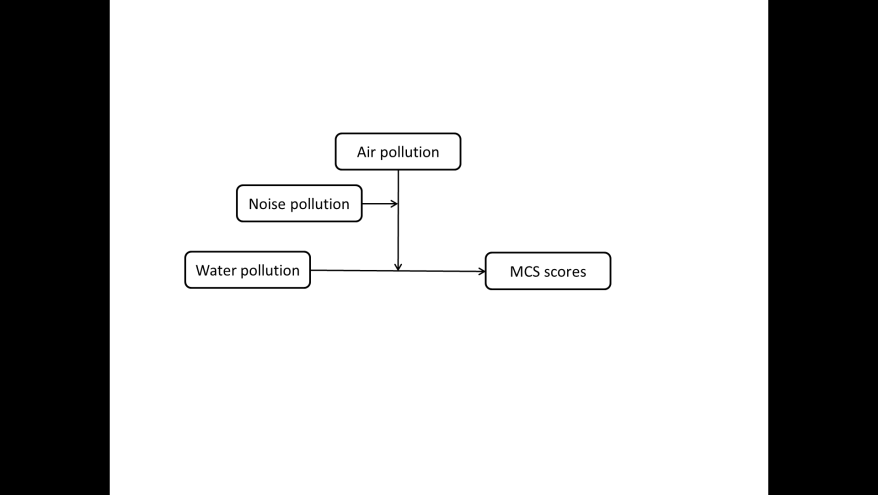


Figure 35. Conceptual diagram

Model = 3 Y = agg_ment X = water M = air W = noise

Statistical Controls:CONTROL= employment bmi hhdnum religion alcohg smokg

Sample size 3697

**************************************************************************

Outcome: agg_ment

Model Summary

R R-sq MSE F df1 df2 p

.15 .02 86.64 6.16 13.00 3683.00 .00

Model

coeff se t p LLCI ULCI

constant 42.59 1.21 35.16 .00 40.21 44.96

air -.51 .44 -1.14 .25 -1.38 .36

water -.87 .46 -1.88 .06 -1.78 .04

int_1 .43 .91 .48 .63 -1.35 2.21

noise -.50 .42 -1.17 .24 -1.33 .34

int_2 -1.61 1.07 -1.51 .13 -3.70 .48

int_3 .89 .87 1.02 .31 -.82 2.60

int_4 4.22 1.85 2.28 .02 .59 7.86

employment -.78 .34 -2.30 .02 -1.44 -.11

bmi .25 .05 5.33 .00 .16 .34

hhdnum -.07 .14 -.52 .60 -.35 .20

religion .51 .47 1.07 .29 -.42 1.44

alcohg .53 .37 1.44 .15 -.19 1.25

smokg 1.22 .35 3.46 .00 .53 1.92

Interactions:

int_1 water X air

int_2 water X noise

int_3 air X noise

int_4 water X air X noise

*************************************************************************

Conditional effect of X on Y at values of the moderator(s):

noise air Effect se t p LLCI ULCI

-.30 -.29 -.15 .65 -.23 .82 -1.42 1.12

-.30 .71 -.98 .93 -1.05 .29 -2.81 .85

.70 -.29 -2.99 1.26 -2.37 .02 -5.46 -.52

.70 .71 .40 .75 .54 .59 -1.07 1.88

Conditional effect of X*M interaction at values of W:

noise Effect se t p LLCI ULCI

-.30 -.83 1.13 -.73 .46 -3.06 1.39

.70 3.39 1.47 2.31 .02 .51 6.27

**************************************************************************

Data for visualizing conditional effect of X on Y

Paste text below into a SPSS syntax window and execute to produce plot.

DATA LIST FREE/water noise air agg_ment.

BEGIN DATA.

-.27 -.30 -.29 49.17

.73 -.30 -.29 49.02

-.27 -.30 .71 48.61

.73 -.30 .71 47.64

-.27 .70 -.29 49.17

.73 .70 -.29 46.18

-.27 .70 .71 48.38

.73 .70 .71 48.79

**Statistical outcomes of potential conceptual diagram 36.**


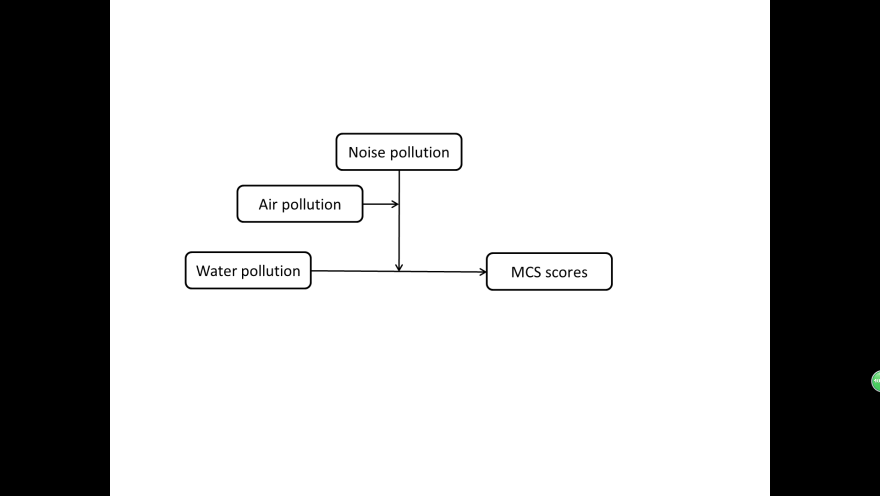


Figure 36. Conceptual diagram

Model = 3 Y = agg_ment X = water M = noise W = air

Statistical Controls:

CONTROL= employment bmi hhdnum religion alcohg smokg

Sample size 3697

**************************************************************************

Outcome: agg_ment

Model Summary

R R-sq MSE F df1 df2 p

.15 .02 86.64 6.16 13.00 3683.00 .00

Model

coeff se t p LLCI ULCI

constant 42.59 1.21 35.16 .00 40.21 44.96

noise -.50 .42 -1.17 .24 -1.33 .34

water -.87 .46 -1.88 .06 -1.78 .04

int_1 -1.61 1.07 -1.51 .13 -3.70 .48

air -.51 .44 -1.14 .25 -1.38 .36

int_2 .43 .91 .48 .63 -1.35 2.21

int_3 .89 .87 1.02 .31 -.82 2.60

int_4 4.22 1.85 2.28 .02 .59 7.86

employment -.78 .34 -2.30 .02 -1.44 -.11

bmi .25 .05 5.33 .00 .16 .34

hhdnum -.07 .14 -.52 .60 -.35 .20

religion .51 .47 1.07 .29 -.42 1.44

alcohg .53 .37 1.44 .15 -.19 1.25

smokg 1.22 .35 3.46 .00 .53 1.92

Interactions:

int_1 water X noise

int_2 water X air

int_3 noise X air

int_4 water X noise X air

*************************************************************************

Conditional effect of X on Y at values of the moderator(s):

air noise Effect se t p LLCI ULCI

-.29 -.30 -.15 .65 -.23 .82 -1.42 1.12

-.29 .70 -2.99 1.26 -2.37 .02 -5.46 -.52

.71 -.30 -.98 .93 -1.05 .29 -2.81 .85

.71 .70 .40 .75 .54 .59 -1.07 1.88

Conditional effect of X*M interaction at values of W:

air Effect se t p LLCI ULCI

-.29 -2.84 1.42 -2.00 .05 -5.62 -.06

.71 1.38 1.20 1.16 .25 -.96 3.73

**************************************************************************

Data for visualizing conditional effect of X on Y

Paste text below into a SPSS syntax window and execute to produce plot.

DATA LIST FREE/water air noise agg_ment.

BEGIN DATA.

-.27 -.29 -.30 49.17

.73 -.29 -.30 49.02

-.27 -.29 .70 49.17

.73 -.29 .70 46.18

-.27 .71 -.30 48.61

.73 .71 -.30 47.64

-.27 .71 .70 48.38

.73 .71 .70 48.79
